# Maslinic Acid-Enriched Diet Decreases Intestinal Tumorigenesis in Apc^Min/+^ Mice through Transcriptomic and Metabolomic Reprogramming

**DOI:** 10.1371/journal.pone.0059392

**Published:** 2013-03-18

**Authors:** Susana Sánchez-Tena, Fernando J. Reyes-Zurita, Santiago Díaz-Moralli, Maria Pilar Vinardell, Michelle Reed, Francisco García-García, Joaquín Dopazo, José A. Lupiáñez, Ulrich Günther, Marta Cascante

**Affiliations:** 1 Department of Biochemistry and Molecular Biology, Faculty of Biology, Universitat de Barcelona, Barcelona, Spain; 2 Institute of Biomedicine, Universitat de Barcelona and CSIC-Associated Unit, Barcelona, Spain; 3 Department of Biochemistry and Molecular Biology, University of Granada, Granada, Spain; 4 Department of Physiology, Faculty of Pharmacy, University of Barcelona, Barcelona, Spain; 5 Henry Wellcome Building for Biomolecular NMR Spectroscopy, CR UK Institute for Cancer Studies, University of Birmingham, Birmingham, United Kingdom; 6 Functional Genomics Node, National Institute of Bioinformatics, Centro de Investigación Pricipe Felipe, Valencia, Spain; 7 Department of Bioinformatics, Centro de Investigación Pricipe Felipe, Valencia, Spain; 8 Centro de Investigación Biomédica En Red de Enfermedades Raras, Valencia, Spain; University College London, United Kingdom

## Abstract

Chemoprevention is a pragmatic approach to reduce the risk of colorectal cancer, one of the leading causes of cancer-related death in western countries. In this regard, maslinic acid (MA), a pentacyclic triterpene extracted from wax-like coatings of olives, is known to inhibit proliferation and induce apoptosis in colon cancer cell lines without affecting normal intestinal cells. The present study evaluated the chemopreventive efficacy and associated mechanisms of maslinic acid treatment on spontaneous intestinal tumorigenesis in Apc^Min/+^ mice. Twenty-two mice were randomized into 2 groups: control group and MA group, fed with a maslinic acid–supplemented diet for six weeks. MA treatment reduced total intestinal polyp formation by 45% (P<0.01). Putative molecular mechanisms associated with suppressing intestinal polyposis in Apc^Min/+^ mice were investigated by comparing microarray expression profiles of MA-treated and control mice and by analyzing the serum metabolic profile using NMR techniques. The different expression phenotype induced by MA suggested that it exerts its chemopreventive action mainly by inhibiting cell-survival signaling and inflammation. These changes eventually induce G1-phase cell cycle arrest and apoptosis. Moreover, the metabolic changes induced by MA treatment were associated with a protective profile against intestinal tumorigenesis. These results show the efficacy and underlying mechanisms of MA against intestinal tumor development in the Apc^Min/+^ mice model, suggesting its chemopreventive potential against colorectal cancer.

## Introduction

Chemoprevention based on the use of bioactive plant compounds has emerged as a practical approach to decrease the risk of various cancers, including colorectal cancer, which is one of the most frequent malignancies and one of the leading causes of cancer-related death in western countries. Familial adenomatous polyposis (FAP), a hereditary colorectal cancer predisposition syndrome, is caused by a mutated adenomatous polyposis coli (*Apc*) gene. FAP patients develop numerous colonic adenomas progressing to colorectal cancer and small intestinal adenomas in most cases. Interestingly, the Apc^Min/+^ mouse, a common animal model of intestinal tumorigenesis, harbors a mutation in the same gene that causes FAP and, like FAP patients, develops large numbers of intestinal tumors at an early age [1]. Therefore, the Apc^Min/+^ mouse model is considered to be an analog of human intestinal tumorigenesis and is extensively used to study chemotherapeutic agents for humans.

Natural products have been exploited for treatment of human diseases for thousands of years. Maslinic acid (MA), a natural pentacyclic triterpene, is widely present in dietary plants, especially in olive fruit skins. This compound has attracted much interest due to its proven pharmacologic safety and its many biological activities, such as anti-viral [2] and antidiabetogenic [3] functions. More recently, some studies have shown that MA has anti-cancer capacity in different cell types, including melanoma [4], liver cancer [5], astrocytoma [6] and colon cancer. Specifically in colon malignancies, MA possesses potent differentiating and anti-proliferation properties, inducing cell-cycle arrest in the G0/G1 phase and apoptosis in colon cancer cells without affecting non-tumor cells [7]. However, because only a few, mainly *in vitro*, studies have aimed to characterize the mechanisms of action of olive components in colon cancer, further research is required.

Therefore, the main objective of the current study was to determine the efficacy of MA consumption in preventing spontaneous intestinal tumorigenesis in Apc^Min/+^ mice and to characterize the mechanisms by which MA executes its function.

## Materials and Methods

### Animals and Treatment

A total of 22 male 4-week-old Apc^Min/+^ mice were purchased from the Jackson Laboratories (Bar Harbor, ME) and maintained in the animal facility at the University of Barcelona. Animal care was strictly in accordance with the European Union Regulations. The experimental protocols were approved by the Experimental Animal Ethical Research Committee of the University of Barcelona in accordance with current regulations for animal care and use for experimental purposes. MA was obtained from olive pomace by using the method described by Garcia-Granados et al. [8]. The extract used was a white powder comprising 98% maslinic acid and 2% oleanolic acid. This extract is stable when stored at 4°C. After a 7-day acclimatization period receiving the standard diet (Teklad Global 18% Protein rodent diet), animals were randomly divided into two groups of 12 and 10 mice per group (Control and MA, respectively). Control mice were fed with the standard diet, and the MA-treated group was fed with the same diet supplemented with 100 mg of MA/kg feed in order to mimic the effective concentration inhibiting colon cancer cell growth [7]. Diets were purchased from Harlan Interfauna Iberica S.L (Barcelona, Spain). Animals were maintained for 12 h light/dark cycles, with free access to water and food. Throughout the 6-week treatment period, animals were observed for any signs of toxicity; body weights and food and water intake were recorded weekly. At the end of the 6 weeks, the animals were starved overnight and anesthetized with volatile isoflurane (ESTEVE, Barcelona, Spain) before blood samples were obtained by cardiac puncture. Finally, mice were killed by an overdose of anesthesia.

### Measurement of Intestinal Polyps

Apc^Min/+^ mice develop polyps in both the small and large intestine, with a greater incidence of intestinal adenomas observed in the former. Therefore, immediately after the mice were killed, the small intestine was excised from each mouse, cut longitudinally, and rinsed with phosphate-buffered saline solution (pH 7.4) to remove intestinal contents. Intestines were pinned flat on cardboard and then were fixed for 1 day in 4% neutral-buffered formalin solution (v/v; pH 7.4). Intestinal sections were stored at room temperature in 1% neutral buffered formalin solution (v/v) until further analysis. To facilitate tumor quantification and identification, the small intestine was divided into three equal-length sections: proximal, medial, and distal. Thereafter, the small-intestine sections were stained in phosphate-buffered saline solution (pH 7.4) containing 0.1% (v/v) methylene blue. By using a stereomicroscope and a measured grid, tumor number and dimensions were determined for each small-intestine section. The size of each intestine tumor was categorized as <1 mm, 1–1.9 mm, or ≥2 mm.

### RNA Isolation and Gene Profiling by Affymetrix Microarrays

The large intestine of each dead mouse was removed and placed on a plastic plate, which was kept at 4°C. After removal of the rectum, the colon was opened longitudinally with fine scissors, and mucus and feces were washed away. The colonic mucosal layer was incubated in Trizol (Invitrogen, Carlsbad, CA) for 3 min and scraped off of the muscle layer with the edge of a sterile glass slide. Cells were transferred into 800 µL Trizol, homogenized by pipetting, and stored at −80°C until RNA extraction. RNA was isolated by using a combination of two methods. First, total RNA was isolated by using the Trizol method according to the manufacturer’s protocol (Invitrogen, Carlsbad, CA). Subsequently, it was purified by using the RNeasy Mini kit and digesting it with DNase I (Qiagen, Germantown, MD) according to the manufacturer’s protocol. RNA pellets were resuspended in DEPC-treated, RNase-free water, and their purity and quantity were determined spectrophotometrically by using the NanoDrop ND-1000 (NanoDrop Technologies). RNA samples were considered suitable for further processing if their absorbance ratio 260/280 was higher than 1.9. Integrity was tested by using lab-on-a-chip technology on the BioAnalyzer 2100 (Agilent, Palo Alto, CA, USA). Samples were considered intact if they had an RNA integrity number (RIN) above 8. Affymetrix microarrays on the Mouse Genome 430 2.0 platforms were performed according to the protocols published by the manufacturer (Affymetrix). Five RNA samples chosen randomly from the control and the MA group were analyzed.

### Microarray Data Analyses

Data was standardized by using the Robust Multi-array Average method [9] and quantile normalization. Differential gene expression was assessed using the *limma* [10] package from Bioconductor. Multiple testing adjustment of p-values was carried out as described by Benjamini and Hochberg [11]. Biochemical pathway analysis was conducted using Kyoto Encyclopedia of Genes and Genomes (KEGG) Mapper, a collection of KEGG mapping tools for KEGG pathway mapping. The Search&Color Pathway tool was used to overlay gene expression results from microarrays onto biochemical pathways found in KEGG. Gene expression levels were denoted by color codes displayed on the pathway by gene symbol boxes. Different shapes and pattern boxes were used to represent induced and suppressed gene expression. Enrichment analysis was based on MetaCore, an integrated knowledge database and software suite for pathway analysis of experimental data and gene lists. Enrichment analysis consisted of matching gene IDs of possible targets for the “common”, “similar”, and “unique” sets with gene IDs in functional ontologies in MetaCore. The probability of a random intersection between a set of IDs and the size of target list with ontology entities was estimated by the p-value of hypergeometric intersection. A lower p-value indicates higher relevance of the entity to the dataset, which shows in a higher rating for the entity. The use of the False Discovery Rate (adjusted p-value) allowed processes with doubtful significance in the experiment to be rejected and ensures that findings are not contaminated with false positives.

### RT Real-Time PCR

The cDNA was synthesized in a total volume of 20 µL by mixing 1 µg of total RNA, 125 ng of random hexamers (Roche), 0.01 M dithiothreitol (Invitrogen), 20 units of RNAsin (Promega), 0.5 mM dNTPs (Bioline), 200 units of M-MLV reverse transcriptase (Invitrogen), and 4 µL 5× First-Strand Buffer (375 mM KCl, 15 mM MgCl_2_, 250 mM Tris-HCl, pH 8.3) (Invitrogen). The reaction mixture was incubated at 37°C for 60 min. The cDNA product was used for subsequent real-time PCR amplification. The mRNA levels of the selected genes were determined in an ABI Prism 7000 Sequence Detection System (Applied Biosystems) by using 9 µL of the cDNA mixture and 11 µL of the specific primers in Master mix (all from Applied Biosystems). β2 microglobulin (B2M) RNA was used as an endogenous control. The reaction was performed following the manufacturers recommendations. Fold-changes in gene expression were calculated by using the standard ΔΔCt method. Experiments were carried out twice using four samples each time for each condition.

### Serum Sampling and NMR Metabolic Analysis

Blood samples were obtained by cardiopuncture of anesthetized mice, and serum samples were obtained by centrifuging blood at 600 g at 4°C for 10 min. Macromolecules were removed from the serum samples by using the ultrafiltration method described by Günther et al. [12]. Briefly, NanoSep 3 K Omega centrifugal devices were prepared by washing them 10 times with 0.5 mL water +0.75 g/L sodium azide at 1100 g and 30°C for 1 h. Prior to use, the mouse samples were stored at −80°C. When needed, samples were thawed, filtered, and then spun at 9000 g at 4°C for 45 min. Then, 150 µL of the filtrate was diluted to obtain a 600-µL NMR sample in buffer (60****mmol/L sodium phosphate, 10 mmol/L sodium azide, 0.5 mmol/L TMSP, 10% D_2_O, pH 7.0). The samples were analyzed using a Bruker 500 spectrometer operating at 500.18 MHz with a 5 mm triple resonance probe at 25°C. One-dimensional ^1^H NMR spectra were obtained by using 128 transients, 16 steady-state scans, a spectral width of 6009 Hz, 8192 pairs data points, and a 4.3 s recycling time. Excitation sculpting was used for water suppression. The data were processed in NMRLab [13]. An exponential line-broadening function of 0.3 Hz was applied, and the dataset was zero-filled to 16384 data points prior to Fourier transformation. Spectra were phase-corrected manually and referenced to TMSP (at 0 ppm). To compare peak volumes, the total area of each spectrum, excluding the regions containing the residual water signal and the TMSP signal, was normalized to 1. The peaks were identified and quantified using the Chenomx NMR Suite with associated libraries (version 4.5; Chenomx Inc., Edmonton, Canada).

## Results

### MA Inhibits Intestinal Tumorigenesis in APC^Min/+^ Mice

**Figure 1:**
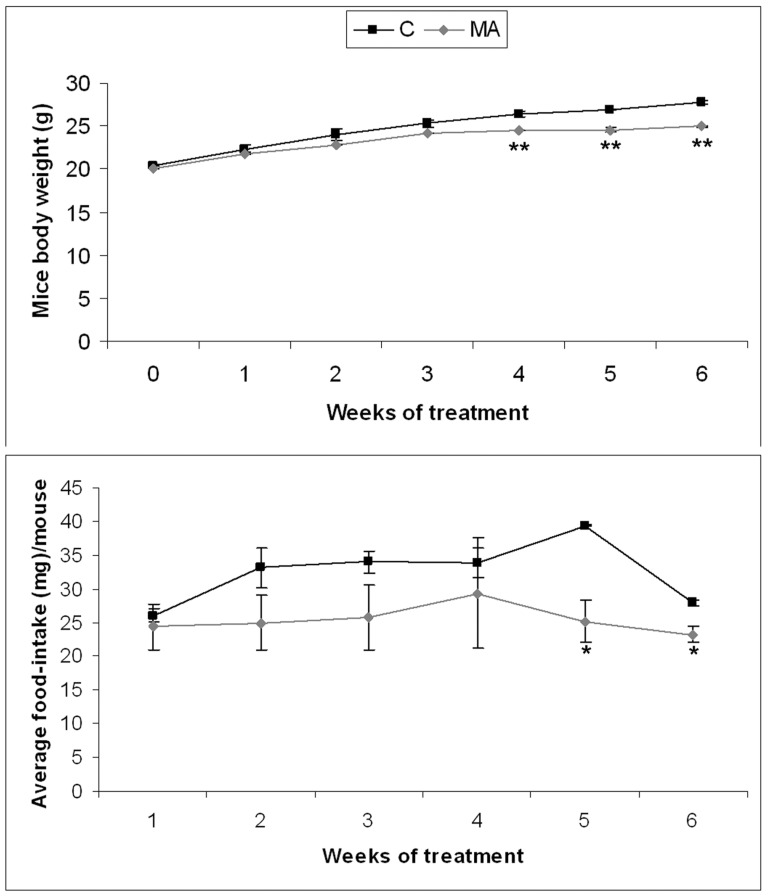
Body weight and diet consumption monitoring. A) Effects of MA treatment on body weight. B) Effects of MA feeding in food intake. Data represented as mean ± SEM (* *, p<0.01).

During the experiment, all mice were monitored for body weight and diet consumption. For the last three weeks, Apc^Min/+^ mice fed with MA showed significantly lower body weight gains than did controls (Figure 1A). Moreover, MA-treated mice showed a reduced food intake for the last two weeks (Figure 1B). However, none of the animals fed with MA produced any sign of distress or any gross changes in any organ, including liver, lung, and kidney.

**Figure 2:**
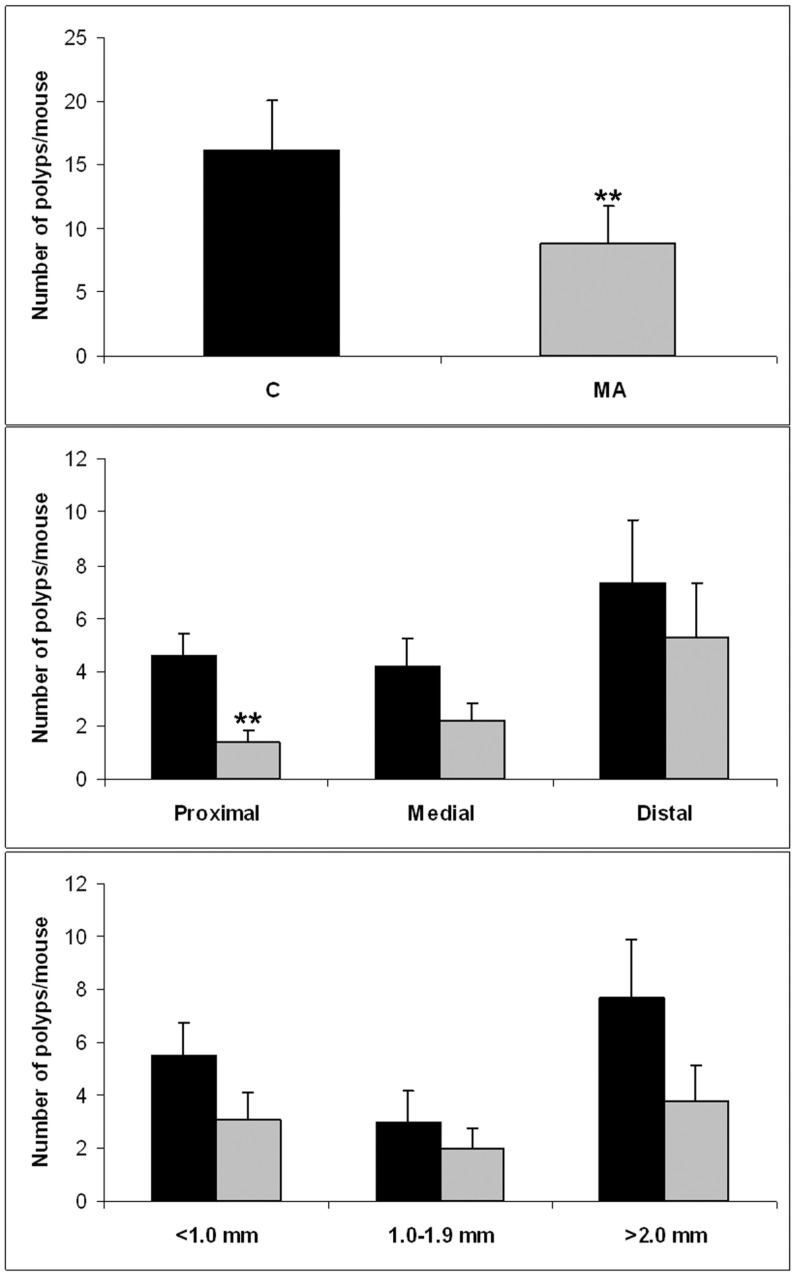
MA feeding inhibits intestinal polyposis in APC^Min/+^ mice. A) Total number of polyps/mouse in the small intestine of Apc^Min/+^ mice. B) Number of polyps/mouse in proximal, medial and distal sections. C) Number of polyps/mouse shown by polyp size distribution (<1 mm diameter polyps, 1–2 mm and >2 mm). Data represented as mean ± SEM (* *, p<0.01).

As shown in Figure 2A, MA prevented spontaneous intestinal polyposis in Apc^Min/+^ mice. Dietary feeding with MA at 100 mg/kg of feed significantly (P<0.01) suppressed intestinal polyp formation by about 45% (9 tumors per mouse) when compared with the control diet group (16 tumors per mouse). The most important MA chemopreventive effect was observed on proximal polyps (69%), followed by medial (4%) and distal polyps (28%) (Figure 2B). In size distribution analysis of polyps, MA reduced the occurrence or growth of <1 mm diameter polyps by 44%, of 1–2 mm diameter polyps by 33%, and of >2 mm diameter polyps by 50% (Figure 2C).

### Gene Expression Profile Induced by MA

To elucidate the underlying mechanisms by which MA inhibits intestinal tumorigenesis in Apc^Min/+^ mice, we determined the transcriptional profile of the Apc^Min/+^ mice’s colonic mucosa by performing cDNA microarray analysis after MA feeding.

In the present study, we analyzed the expression profile of 45101 genes by performing whole mouse genome cDNA microarrays. MA supplementation changed the expression of 2375 genes (p-value <0.05), with an absolute fold-change of 1.5 or more. Of these 2375 differentially expressed genes, 193 were upregulated, and 2182 were downregulated (Table S1).

**Figure 3:**
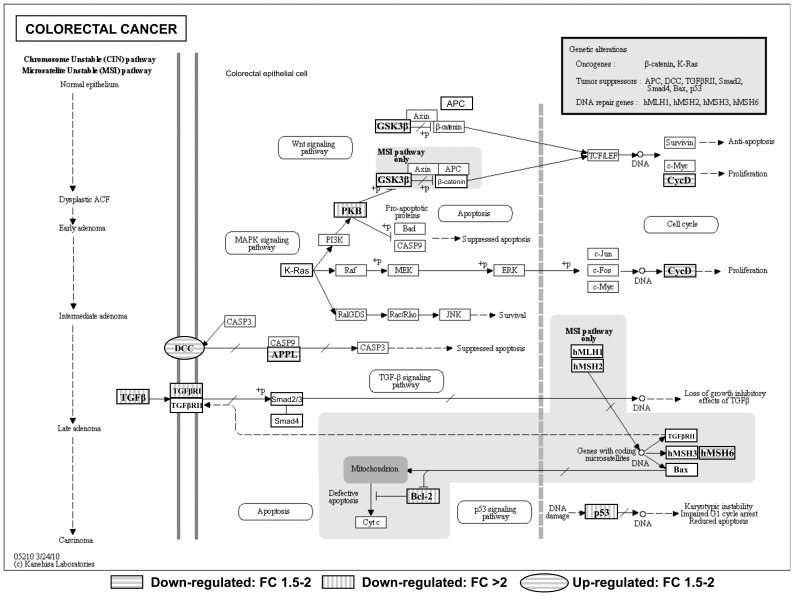
Adaptation of KEGG colorectal cancer pathway using KEGG Mapper. Circular pathway members were significantly up-regulated and rectangular members were found to be down-regulated in the intestinal mucosa of Apc^Min/+^ mice treated with MA. Horizontal lines indicate a fold change (FC) of between 1.5 and 2 and vertical lines a FC of more than 2.

First, the list of differentially expressed genes between non-treated and MA-treated mice was subjected to a KEGG molecular pathway analysis using the KEGG Mapper tool to identify possible enrichment of genes with specific biological activities. Figure 3 depicts the KEGG colorectal cancer pathway using KEGG Mapper and shows that MA downregulated glycogen synthase kinase 3β (*Gsk3b*), a protein involved in Wnt/β-catenin signaling that is affected in Apc^Min/+^ mice. Interestingly, MA also inhibited Cyclin D *(Ccnd1)* (Figure 3), a gene expressed after the transcriptional activation of β-catenin.

Moreover, MA treatment downregulated the expression of the *Akt1* gene, which codes for the protein AKT (protein kinase B, PKB) (Figure 3), a serine/threonine kinase critical in controlling cell survival, insulin signaling, angiogenesis, and tumor formation; the *Tpr53* gene (Figure 3), encoding protein p53, which regulates cell cycle, apoptosis, senescence, metabolism, and DNA repair; the *Msh6* gene (Figure 3), involved in the post-replicative DNA mismatch repair system (MMR) and the *Tgfb1* gene and its receptor (*Tgfb1r1*) (Figure 3).

On the other hand, MA caused upregulation of deleted in colorectal carcinoma (*Dcc)* gene (Figure 3), encoding the pro-apoptotic protein DCC. However, MA also downregulated DIP13α (*Appl1*), a mediator of the DCC apoptotic pathway (Figure 3). Furthermore, MA reduced the expression of the anti-apoptotic protein Bcl-2 (Figure 3).

Pathway analysis performed using KEGG Mapper was complemented with an independent analysis by MetaCore to obtain a p-value for each pathway. According to the GeneGO Maps Folder in Metacore, the maps containing genes corresponding to cytoskeleton remodeling, transcription, cell cycle, cell adhesion, immune response, apoptosis, and survival in normal and pathologic TGF-β-mediated regulation of cell proliferation were the most significantly modulated (Table 1). In addition to the aforementioned cell-cycle-associated genes, Metacore analysis identified downregulation of *Cdk4*, *Cdk6*, *Btrc*, *Junb*, and *Ppp2r4* (Table 1, cell cycle). On the other hand, apart from the apoptosis-related genes already mentioned, Metacore analysis revealed the downregulation of the anti-apoptotic gene *Bcl2l1* (Bcl-XL) (Table 1, apoptosis and survival). Moreover, diverse genes involved in signal transduction pathways that avoid apoptosis have been shown to be modulated in MA-treated mice. Concretely, MA downregulated *Shc1*, *Grb2*, *Sos1*, *Rps6ka2*, *Ywhae*, *Ywhag*, *Prkar2b,* and *Prkaca* gene expression.

**Table 1:** Pathways modified in the colon mucosa of Apc^Min/+^ mice by MA treatment as found in Metacore.

GeneGO Maps/Modulated pathways	$p-value	‡Significant/total genes	Modulated genes
*Cytoskeleton remodeling*			
TGF, WNT and cytoskeletal remodeling (↓)	3,57E−09	23/111	*Ncl, Tgfb1, Tgfbr1, Wnt5a, Fzd7, Dvl1, Dock1, Akt1, Gsk3b, Map3k7, Mapk14, Limk2, Ppard, Trp53, Ccnd1, Cfl1, Actn1, Arpc4, Sos1,Grb2, Pxn, Tln1, Shc1*
Cytoskeleton remodeling (↓)	4,91E−07	16/102	*Ncl, Tgfb1, Tgfbr1, Dock1, Gsk3b, Map3k7, Mapk14, Limk2, Cfl1, Actn1, Arpc4, Sos1,Grb2, Pxn, Tln1, Shc1*
*Transcription*			
CREB pathway (↓)	1,18E−07	16/44	*Akt1, Mapk14, Ccnd1, Sos1, Grb2, Shc1, Clca2, Camk2g, Gprc5a, Prkcb, Prkar2b, Rps6ka2, Cdo1, Prkaca, Fbxw5, Fbxw11*
*Cell cycle*			
Regulation of G1/S transition (part 1) (↓)	1,61E−07	11/38	*Cdk4, Cdk6, Junb, Btrc, Ppp2r4, Anapc1, Tgfb1, Tgfbr1, Gsk3b, Ccnd1, Ccnd2*
*Cell adhesión*			
Chemokines and adhesion (↓)	3,55E−07	19/100	*Dock1, Akt1, Gsk3b, Limk2, Cfl1, Actn1, Arpc4, Sos1,Grb2, Pxn, Tln1, Shc1, Thbs1, Cd44, Cd47, Itga3, Msn, Flot2, Eif4g1*
*Immune response*			
Signaling pathway mediated by IL-6 and IL-1 (↓)	3,56E−06	9/27	*Sos1,Grb2, Shc1, Il6st, Jak1, Ikbkap, Nfkbie, Irak1, Cebpb*
IL-15 signaling (↓)	2,19E−06	12/64	*Akt1, Mapk14, Sos1,Grb2, Shc1, Il2rg, Adam17, Nfkbie, Prkce, Ets1, Bcl2, Bcl2l1*
MIF - the neuroendocrine-macrophage connector (↑)	1,92E−04	3/46	*Plcb2, Pla2g4c, Itpr2*
PIP3 signaling in B lymphocytes (↑)	1,34E−04	5/42	*Plcb2, Pik3r1, Itpr2, Foxo3, Igh-6*
*Apoptosis and survival*			
BAD phosphorylation (↓)	4,21E−06	11/42	*Bcl2, Bcl2l1, Sos1, Grb2, Shc2, Rps6ka2, Ywhae, Ywhag, Ppm1g, Prkar2b, Prkaca*
*Normal and pathological TGF-beta-mediated regulation of cell proliferation*			
Normal and pathological TGF-beta-mediatedregulation of cell proliferation (↓)	2,71E−06	10/33	*Tgfb1, Tgfbr1, Gsk3b, Mapk14, Trp53, Ccnd1,Sos1,Grb2, Shc1, Map2k6*

More significantly modulated pathways in Metacore using genes with FC>1.5 and adjusted p-value<0.01. ↑/↓, activation/inhibition of the biological process by MA;

$ p-value that corresponds to the GeneGO Map/Pathway.

‡ Ratio between the number of significantly modulated genes by MA and the total number of genes per GenenGO Map/Pathway in Metacore.

### Validation of Microarray Data by RT-PCR

**Figure 4:**
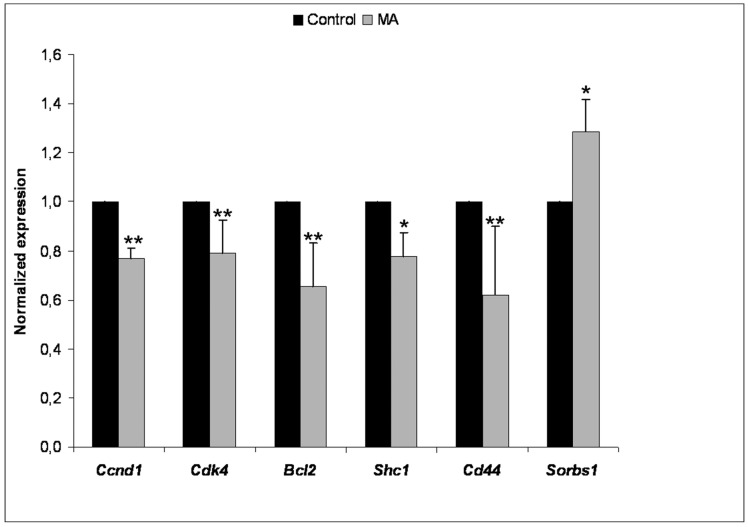
Validation of genes that were differentially expressed in the colon mucosa of Apc^Min/+^ mice after MA treatment by RT-PCR. Mean ± SD are shown. *, p<0.05; * *, **p<0.01, versus the untreated condition. n = 8/group.

The changes in mRNA expression observed in the microarrays for *Ccnd1*, *Cdk4, Bcl2, Shc1, Cd44* and *Sorbs1* were validated by performing RT real-time PCR assays (Figure 4). These targets were selected for RT real-time PCR analysis on the basis of their significant participation in the chemopreventive effects produced in Apc^Min/+^ mice by MA supplementation.

### Metabolic Profile of Blood Serum Induced by MA

^1^H NMR spectroscopy detected a wide range of metabolites in Apc^Min/+^ mice blood serum. Upon analyzing the spectra, several metabolites were seen to vary between MA-treated and control groups. Whereas glucose and 3-hydroxybutyrate were clearly different between the two groups, some metabolites, such as acetoacetate, acetate, acetone, lactate, valine, alanine, leucine, lysine and creatine, followed an imperfect trend with sample dependent variations (Table 2). Quantification and comparison of ^1^H NMR results for well-resolved peaks showed that MA supplementation gave 3-hydroxybutyrate levels of 125±12% in the MA group compared to the control group whereas it reduced the levels of glucose to 89±9% of that of the control group (Table 2). Moreover, other metabolites, including citrate, pyruvate, glutamine, phenylalanine, tyrosine, isoleucine, urea and allantoin, were clearly identified but did not show differences between the MA and control groups (Table 2).

**Table 2:** List of metabolites identified for ^1^H NMR data by Chenomx database in Apc^Min/+^ mice serum.

Serum samples from maslinic acid-treated mice were:				
Perfect trends	AUC Control	AUC MA	‡% MA/Control	$p-value
↓ Glucose	3.930e+09±1.531e+08	3.504e+09±2.353e+08	89±9	0,0229
↑ 3-Hydroxybutyrate	3.168e+08±1.182e+07	3.960e+08±2.206e+07	125±12	0,0007
**Imperfect trends**				
↑ Acetoacetate				
↑ Acetate				
↑ Acetone				
↓ Lactate				
↓ Valine				
↓ Alanine				
↓ Leucine				
↓ Lysine				
↓ Creatine				
**No pattern**				
Citrate				
Pyruvate				
Glutamine				
Phenylalanine				
Tyrosine				
Isoleucine				
Urea				
Allantoin				

↑/↓ Higher/Lower in MA-fed group when compared with the control diet group.

‡ Ratio between the area under the curve (AUC) in MA and the AUC in controls for the corresponding metabolite.

$ p-value relative to difference between MA and control.

## Discussion

MA supplementation inhibits spontaneous intestinal polyposis without producing any sign of distress or toxicity in APC^ Min/+^ mice. MA-treated mice showed a loss of weight (Figure 1A) that, at least partly, could be attributed to the reduced food intake (Figure 1B). In turn, the decrease in food intake might be related to a satiety effect or differences in energy metabolism produced by MA [14].

MA treatment significantly reduced total intestinal polyp formation in Apc^Min/+^ mice (Figure 2A). However, this effect was statistically nonsignificant, probably due to fewer polyps and high variability, when polyps were classified by size or zone, except for polyps in the proximal small intestine (Figure 2B & C). MA showed differential efficacy suppressing intestinal polyp formation depending on small-intestine segment (Figure 2B). This is in agreement with previous evidence that some dietary and pharmaceutical compounds provide cancer protection only in parts of the small intestine [15]. These effects could be related to several physiologic conditions through the gastrointestinal tract, such as pH, expression pattern of several enzymes, microbiota, and concentration due to intestinal content. All these conditions can modify the chemical structure of a chemopreventive agent and influence its final metabolism and, consequently, its anticancer effect. For example, resveratrol is almost completely conjugated upon oral administration, and the most bioactive metabolites are its glucuronide and sulfate derivatives [16], [17]. The inhibitory efficacy depending on polyp size was more homogeneous, suggesting that MA inhibits both the appearance and development of intestinal polyps (Figure 2C).

Comparison of microarray expression profiles of MA-treated and control mice revealed that MA downregulates the expression of the *Gsk3b* (GSK3β) gene (Figure 3). As mentioned above, Apc^Min/+^ mice contain a mutation in APC that, together with Axin and GSK3β, operates by activating β-catenin degradation in Wnt/β-catenin signaling pathway. Therefore, the mutation of the *Apc* gene present in the Apc^Min/+^ mouse produces a cytosolic accumulation and an increase in nuclear translocation of β-catenin. In the nucleus, β-catenin interacts with the transcription factor T cell factor/lymphoid enhancer factor (TCF/LEF), leading to an increase in the expression of survival genes, including *c-Myc*, *Cyclin D1*, and *Cyclooxygenase-2* (*Cox-2*) [18]. However, GSK3β has also been linked to a prosurvival signal in a Wnt/β-catenin-independent manner. In this regard, GSK3β is constitutively activated in colon cancer cells, where it is implicated in tumorigenesis and cancer progression. Accordingly, the downregulation of GSK3β inhibits proliferation and enhances apoptotic cell death in chronic lymphocytic leukemia B cells, renal cancer cells, pancreatic cancer cells, and colon cancer cells [19], [20]. These results may indicate that MA confers a protective effect by inhibiting GSK3β.

Overexpression of *Akt* is an early event in colorectal carcinogenesis [21], thus the lower expression of *Akt* in MA-treated mice may be related to the inhibition of intestinal polyp growth in Apc^Min/+^ mice. Another common clinicopathologic feature of colorectal carcinoma is the presence of mutations in p53 (*Tpr53*). Regarding APC^Min/+^ mice, p53 inactivation has been reported to have little effect on the incidence or the progression and apoptosis of adenomas [22]. Nevertheless, inhibition of p53 in mice treated with MA indicates that the death process is p53-independent. Furthermore, advanced colorectal adenomas usually present changes in transforming growth factor beta (TGFβ) signaling. Generally, cancerous cells increase their production of TGFβ, which acts on the secreting cancerous cells themselves and on surrounding cells regulating cell growth, differentiation, and apoptosis [23]. Thus, reduction of TGFβ signaling induced by MA through the downregulation of *Tgfb1* and *Tgfb1r1* may be involved in the inhibition of tumorigenesis in Apc^Min/+^ mice. Also in the advanced stages of colorectal pathogenesis, deleted in colorectal carcinoma (*Dcc)* gene expression appears to be lost or markedly reduced. This gene encodes a netrin 1 receptor that functions as a tumor suppressor via its ability to trigger tumor cell apoptosis [24]. MA caused upregulation of DCC, indicating a pro-apoptotic effect. However, a mediator of the DCC apoptotic effect, DIP13α (*Appl1*), was downregulated by MA treatment. DIP13α interacts with a region on the DCC cytoplasmic domain that is required for the induction of apoptosis [25]. However, DIP13α also binds many other proteins, including RAB5A, AKT2, PI3KCA, and adiponectin receptors to regulate cell proliferation and adiponectin and insulin signaling. Given that little is known about DIP13α, its inhibition by MA could indicate a beginning of MA-resistance in Apc^Min/+^ mice, antagonizing DCC apoptotic activation but also modulating other DIP13α biological functions.

Moreover, expression analysis revealed that the induction of apoptosis by MA is based on the downregulation of the anti-apoptotic genes *Bcl-2* and *Bcl2l1* (Bcl-XL) (Table 1, apoptosis and survival). Therefore, this downregulation by MA acts as a pro-apoptotic stimulus. This finding is in agreement with that of a recent study where western blotting analysis showed that the treatment of the HT29 human colon adenocarcinoma cell line with MA induced the repression of Bcl2 [26]. Besides, different signal transduction pathways that save cells from apoptosis were inhibited by MA. For instance, MA downregulated epidermal growth factor receptor (EGFR) signaling, which is related to mitogenesis and tumorigenesis. After EGFR activation, a trimeric complex between tyrosine phosphorylated Shc, Grb2, and Sos is formed and this, in turn, triggers downstream mitogenic signaling [27]. MA exerted this action by downregulating *Shc1*, *Grb2*, and *Sos1* gene expression. Furthermore, MA treatment reduced the expression of the *Rps6ka2* gene, coding for the protein p90Rsk, a downstream mediator of the mitogen-activated protein kinase (MAPK) pathway, which has been reported to inhibit apoptosis via the stimulation of binding of Bad to 14-3-3 and the inactivation of Bad-mediated cell death [28]. Interestingly, MA also triggered this apoptotic action by inhibiting the expression of *Ywhae* and *Ywhag*, which code for different members of the family of 14-3-3 proteins, and thus reducing Bad sequestration and increasing Bad-induced apoptosis via the mitochondrial death pathway [29]. In addition, MA reduced the expression of *Prkar2b* and *Prkaca* coding for the regulatory subunit type II-beta of the cAMP-dependent protein kinase (PKA RII-beta) and the catalytic subunit alpha of the cAMP-dependent protein kinase (PKA C-alpha), respectively. PKA is a serine/threonine kinase that is activated by cyclic adenosine monophosphate (cAMP). Effects of PKA on apoptosis are likely to be largely dependent on the cell type and the mechanisms by which apoptosis is induced [30]. In the case of Apc^Min/+^ mice, treatment with a PKA antagonist, Rp-8-Br-cAMPS, reduces tumor promotion and β-catenin levels, nuclear translocation, and expression of some of its target genes, such as *c-Myc* and *cyclooxygenase-2* (*Cox-2*) [31].

On the other hand, MA inhibited the expression of some genes related to cell cycle (Table 1, cell cycle). Cell cycle progression is highly controlled by a complex network of signaling pathways that eventually converge to regulate the activity of cyclin/cyclin-dependent kinase (CDK) complexes. There are different members of the CDK family, and each CDK is dependent on a particular cyclin partner [32]. In this regard, MA downregulated the gene expression of Cyclin D *(Ccnd1)*, which drives the G1 phase progression. In agreement with this result, the results of other studies have related MA antitumor activity to an inhibition of Cyclin D1 expression [33]. Moreover, MA reduced the expression of Cyclin D partners during G1 phase, Cdk4 and Cdk6, inhibiting the G1 cyclin-CDK complexes and leading to G1-phase cell-cycle arrest. Specific and timely proteolysis of cell-cycle regulators by the ubiquitin-proteasome system represents an important mechanism that ensures proper progression through the cell cycle in a unidirectional and irreversible manner. Furthermore, in cancer cells, deregulation or suppression of the proteasome is supposed to induce uncontrolled proteolysis and is linked to having a more sensitive profile to drugs than that of normal cells [34]. MA inhibited the ubiquitin ligase SKP1–CUL1–F-box-protein (SCF) complex by downregulating the *Btrc* gene. βTrCP protein pertaining to the F-box family is the substrate-recognition component of the SCF ubiquitin ligase complex, which mediates the ubiquitination and subsequent proteasomal degradation of target proteins involved in cell-cycle checkpoints, protein translation, cell growth, and survival. Interestingly, βTrCP plays an important role allowing G1/S transition and also mediates the degradation of the pro-apoptotic protein BimEL to promote cell survival [35] and has been reported to be overexpressed in colorectal tumors [36]. Hence, its downregulation by MA could be related to cell cycle arrest and subsequent inhibition of spontaneous polyposis in Apc^Min/+^. Additionally, MA modulates other cell-cycle regulatory proteins. For example, MA suppresses the expression of the gene encoding the oncogenic protein JunB, which is an essential component of the activating protein-1 (AP-1) transcription factor that is involved in the control of cell growth, differentiation, inflammation, and neoplastic transformation. It is noteworthy that a recent study demonstrated that the chemopreventive effects of MA in Raji cells depends on the inhibition of nuclear factor-κB (NF-κB) and the activation of Activator protein (AP-1) [37]. Another protein controlling cell growth and division that was downregulated in Apc^Min/+^ mice after treatment with MA was a regulatory subunit of protein phosphatase 2A (PP2A) (*Ppp2r4*). This protein has been described to dephosphorylate β-catenin, acting as a positive regulator of Wnt signaling [38], [39]. Moreover, *Ppp2r4* function is essential for cell survival [38], [39]. Therefore, MA’s downregulation of the gene encoding this protein could be involved in its antitumor effect.

Additionally, evidence is accumulating to suggest that proteins involved in regulating actin cytoskeleton and cell adhesion also participate in mitogenesis, either as unique transducers of growth signals or as monitors of anti-apoptotic conditions, or both [40], [41]. In this regard, MA downregulated several genes related to cytoskeleton remodeling and cell adhesion such as *Cd44* (Table 1, cytoskeleton remodeling and cell adhesion). A recent study using short hairpin RNA against CD44 to silence its expression in SW620 colon cancer cells showed that reduced expression of the protein inhibited cell proliferation, migration, and invasion. In agreement with our results, reduced expression of CD44 induced the same effects that we describe for MA, cell cycle arrest in the G1 phase and apoptosis via the downregulation of Bcl-2 and Bcl-xL, and also via the upregulation of BAX [42].

Regarding DNA repair system, MA inhibited the expression of the *Msh6* gene (Figure 3). DNA repair is associated with the prevention of mutagenesis and cancer but can also be associated with the detection and repair of mismatches derived from chemically induced DNA damage with genotoxic agents. In this regard, the utility of genotoxic drugs is often limited by the enhanced ability of cancer cells to repair their DNA. Therefore, attenuation of the DNA repair system sensitizes tumor cells to DNA-damaging agents [43]. Notably, MA has been reported to interfere with DNA integrity in HT29 cells [26]; hence, it could be acting, at least in part, as a genotoxic agent. In this case, *Msh6* downregulation could trigger DNA damage and posterior apoptosis. Anyway, inhibition of only one DNA repair system would hardly affect final repairing activity due to the functional redundancy of MMR proteins.

Finally, MA also downregulated immune system–related genes (Table 1, immune response). Inflammation and immune system responses have controversial effects in cancer, either by preventing and inhibiting tumor development or, when inflammation becomes chronic, by promoting the growth and progression of cancer [44]. In this regard, chronic inflammation plays a decisive role in the development and sustenance of intestinal adenomatous polyps in the Apc^Min/+^ mice [45]. Accordingly, MA has been implicated in anti-inflammatory and immune-attenuating actions via suppression of NFkB [46]. Therefore, in this case, the downregulation of immune system responses by MA may reduce tumor growth. Regarding also immune function, it is noteworthy that MA inhibits the expression of *Cxcr4* gene (Table S1), encoding for chemokine (C-X-C motif) receptor 4, which allows HIV infection [47]. Hence, this result may explain the anti-HIV activity of MA [48].

On the other hand, ^1^H NMR spectroscopy results can be explained by some of the genetic modulations induced by MA. The decrease in serum glucose concentration in Apc^Min/+^ mice treated with MA could be a consequence of the upregulation of the c-Cbl–associated protein (CAP) encoded by *Sorbs1*. CAP plays a critical role in insulin-regulated GLUT4 translocation [49] and hence, its activation by MA promotes glucose cellular uptake. Moreover, low glucose levels in mice serum can be due to a glycogen accumulation triggered by MA treatment. First, glycogen reservoirs are regulated by the aforementioned GSK3β. This protein, apart from its role in Wnt and pro-survival signaling, is able to phosphorylate and inhibit glycogen synthase activity, impairing glycogen synthesis. Thus, inhibiting GSK3β by MA implies an activation of glycogen accumulation. Second, MA also reduced *Phka1* expression. Because the *Phka1* gene encodes PHK protein, which activates glycogen phosphorylase and leads to the conversion of glycogen into glucose-1-phosphate, by downregulating PHK MA inhibits glycogen degradation. In addition to these transcriptional modifications, MA has been described as a potent direct inhibitor of glycogen phosphorylase, thus triggering glycogen reservoir accumulation [50]. A significantly increase in the ketone body 3-hydroxybutyrate level was also found in serum from MA-treated mice. The decrease in serum glucose may contribute to the increase in serum ketone body concentrations because, although ketone body synthesis occurs normally under all conditions, its formation increases as glucose availability drops. To support the elevated ketone body synthesis in MA-treated mice, high fatty acid oxidation is necessary for the production of acetyl-CoA used as substrate. In mice treated with MA, fatty acid degradation is activated by the upregulation of *Cpt1*, which encodes carnitine palmitoyltransferase I (CPT I), the mitochondrial gateway for fatty acid entry into the matrix and, thus, the main controller of fatty acid oxidation. It is noteworthy that this observation may be involved in the loss of weight detected in Apc^Min/+^ mice after MA treatment. Moreover, our results are in agreement with previous studies describing antihyperlipidemic [51] and antihyperglycemic [14] activities for MA. Given that accumulating evidence suggests that obesity [52] and hyperglycemia [53] are associated with increased risk of colorectal cancer, the metabolic changes induced by MA treatment are potentiating its chemoprotective effect in Apc^Min/+^ mice.

Taken together, our data show that MA is a nontoxic agent that effectively inhibits intestinal polyposis in genetically predisposed Apc^Min/+^ mice. The cancer chemopreventive effects of MA are mainly related to the modulation of cancer progression–related genes, suggesting an induction of a G1-phase cell-cycle arrest and activation of apoptosis by a p53-independent mechanism. Moreover, the expression of genes related to energy metabolism is altered by MA to support a protective metabolic profile. In summary, our findings provide the first *in vivo* evidence that MA is a promising nutraceutical for colon cancer prevention.

## Supporting Information

Table S1 **Differentially expressed genes by MA treatment in the colon mucosa of APC^Min/+^ mice.** List of differentially expressed genes assessed using the limma package from Bioconductor (Fold change>1.5 and adjusted p-value<0.05).
(PDF)
