# Supplementary material for: Maslinic Acid-Enriched Diet Decreases Intestinal Tumorigenesis in ApcMin/+ Mice through Transcriptomic and Metabolomic Reprogramming
Source: PLoS One. 2013 Mar 18;8(3):e59392. doi: 10.1371/journal.pone.0059392 (PMC3601079; doi:10.1371/journal.pone.0059392)
Supplement: Table S1 — Differentially expressed genes by MA treatment in the colon mucosa of APCMin/+ mice. List of differentially expressed genes assessed using the limma package from Bioconductor (Fold change>1.5 and adjusted p-value<0.05). (PDF) [file pone.0059392.s001.pdf]

Table S1. Differentially expressed genes by MA treatment in the colon mucosa of APC$^{Min/+}$ mice

Affimetrix ID	Gene Symbol	Gene description	Fold change	Adjusted p-value
1437438_x_at	Pnliprp2	**pancreatic lipase-related protein 2**	**5,8**	0,009521
1447802_x_at	AV099323	**expressed sequence AV099323**	**3,8**	0,000000
1437812_x_at	Ganab	**alpha glucosidase 2 alpha neutral subunit**	**3,6**	0,000000
1447007_at	1700008I05Rik	**RIKEN cDNA 1700008I05 gene**	**2,6**	0,000000
1447882_x_at	Ddx54	**DEAD (Asp-Glu-Ala-Asp) box polypeptide 54**	**2,5**	0,000000
1450875_at	Gpr37	**G protein-coupled receptor 37**	**2,5**	0,000000
1441935_at	Ankra2	**ankyrin repeat, family A (RFXANK-like), 2**	**2,4**	0,000000
1440180_x_at	Zbtb3	**zinc finger and BTB domain containing 3**	**2,3**	0,000000
1440383_at	Dclre1b	**DNA cross-link repair 1B, PSO2 homolog (S. cerevisiae)**	**2,3**	0,000001
1420132_s_at	Pttg1ip	**pituitary tumor-transforming 1 interacting protein**	**2,1**	0,000003
1447902_at	1810013A23Rik	**RIKEN cDNA 1810013A23 gene**	**2,1**	0,000000
1455792_x_at	Ndn	**necdin**	**2,1**	0,000002
1439296_at	Prickle3	**prickle homolog 3 (Drosophila)**	**2,1**	0,000000
1452538_at	Igh-6	**immunoglobulin heavy chain 6 (heavy chain of IgM)**	**2,0**	0,000001
1418913_at	Bhmt2	**betaine-homocysteine methyltransferase 2**	**2,0**	0,000005
1419911_at	Coro1c	**coronin, actin binding protein 1C**	**2,0**	0,000002
1439224_at			**2,0**	0,001574
1451600_s_at	EG13909	**predicted gene, EG13909**	**2,0**	0,001412
1459047_x_at			**1,9**	0,000004
1436383_at	Cplx2	**complexin 2**	**1,9**	0,000000
1456120_at	Secisbp2l	**SECIS binding protein 2-like**	**1,9**	0,002064
1417197_at	Wwc2	**WW, C2 and coiled-coil domain containing 2**	**1,9**	0,000000
1439124_at	Wdr91	**WD repeat domain 91**	**1,9**	0,000001
1456868_at			**1,9**	0,000002
1440159_at	1700010B09Rik	**RIKEN cDNA 1700010B09 gene**	**1,9**	0,000317
1420175_at	Tax1bp1	**Tax1 (human T-cell leukemia virus type I) binding protein 1**	**1,9**	0,000134
1422425_at	Sprr2k	**small proline-rich protein 2K**	**1,9**	0,004129
1458626_at	Nos1	**nitric oxide synthase 1, neuronal**	**1,9**	0,000009
1422973_a_at	Thrsp	**thyroid hormone responsive SPOT14 homolog (Rattus)**	**1,9**	0,000001
1439144_at	Cwf19l1	**CWF19-like 1, cell cycle control (S. pombe)**	**1,9**	0,000002
1425824_a_at	Pcsk4	**proprotein convertase subtilisin/kexin type 4**	**1,8**	0,000003
1435906_x_at	Gbp2	**guanylate binding protein 2**	**1,8**	0,003678
1438956_x_at	Pim3	**proviral integration site 3**	**1,8**	0,000032
1459115_at			**1,8**	0,000000
1422162_at	Dcc	**deleted in colorectal carcinoma**	**1,8**	0,000002
1452481_at	Plcb2	**phospholipase C, beta 2**	**1,8**	0,000007
1441160_at	D330050G23Rik	**RIKEN cDNA D330050G23 gene**	**1,8**	0,000000
1419349_a_at	Cyp2d9	**cytochrome P450, family 2, subfamily d, polypeptide 9**	**1,8**	0,006830
1419975_at	Scp2	**sterol carrier protein 2, liver**	**1,8**	0,000381

1443152_at	Ptcd3	**pentatricopeptide repeat domain 3**	**1,8**	0,000058
1447892_at			**1,8**	0,000034
1429983_at	2010002M09Rik	**RIKEN cDNA 2010002M09 gene**	**1,8**	0,000010
1458250_at	Fam13c	**family with sequence similarity 13, member C**	**1,8**	0,000002
1450260_at	Grpr	**gastrin releasing peptide receptor**	**1,8**	0,008580
1423804_a_at	Idi1	**isopentenyl-diphosphate delta isomerase**	**1,8**	0,005388
1439834_at	2400009B08Rik	**RIKEN cDNA 2400009B08 gene**	**1,8**	0,000001
1455457_at	Cyp2c54	**cytochrome P450, family 2, subfamily c, polypeptide 54**	**1,7**	0,000046
1456003_a_at	Slc1a4	**solute carrier family 1 (glutamate/neutral amino acid transporter), member 4**	**1,7**	0,000006
1459243_at	100042016	**predicted gene, 100042016**	**1,7**	0,000258
1440642_at	D630042P16Rik	**RIKEN cDNA D630042P16 gene**	**1,7**	0,000005
1443827_x_at	Fam20c	**family with sequence similarity 20, member C**	**1,7**	0,000069
1438615_x_at	2010317E24Rik	**RIKEN cDNA 2010317E24 gene**	**1,7**	0,000000
1439404_x_at	Zfx	**zinc finger protein X-linked**	**1,7**	0,000025
1441840_x_at	Sbno2	**strawberry notch homolog 2 (Drosophila)**	**1,7**	0,000002
1442027_at	Nbeal1	**neurobeachin like 1**	**1,7**	0,000084
1451635_at	AB056442	**cDNA sequence AB056442**	**1,7**	0,000016
1436991_x_at	Gsn	**gelsolin**	**1,7**	0,000197
1432731_at	5830437K03Rik	**RIKEN cDNA 5830437K03 gene**	**1,7**	0,000216
1426520_at	Btg4	**B-cell translocation gene 4**	**1,7**	0,000015
1416023_at	Fabp3	**fatty acid binding protein 3, muscle and heart**	**1,7**	0,000011
1443799_at			**1,7**	0,000078
1436750_a_at	Oxct1	**3-oxoacid CoA transferase 1**	**1,7**	0,000589
1437671_x_at	Prss23	**protease, serine, 23**	**1,7**	0,001541
1459788_at	Gpr107	**G protein-coupled receptor 107**	**1,7**	0,002566
1436737_a_at	Sorbs1	**sorbin and SH3 domain containing 1**	**1,7**	0,000611
1439822_at			**1,7**	0,000002
1423544_at	Ptpn5	**protein tyrosine phosphatase, non-receptor type 5**	**1,7**	0,001058
1423635_at	Bmp2	**bone morphogenetic protein 2**	**1,7**	0,002040
1417461_at	Cap1	**CAP, adenylate cyclase-associated protein 1 (yeast)**	**1,7**	0,004914
1451547_at	Iyd	**iodotyrosine deiodinase**	**1,7**	0,008053
1460055_at			**1,7**	0,000055
1418623_at	Rab2a	**RAB2A, member RAS oncogene family**	**1,7**	0,000017
1444254_at	Tns4	**tensin 4**	**1,7**	0,000004
1439887_at			**1,7**	0,000619
1456701_at	B230208H17Rik	**RIKEN cDNA B230208H17 gene**	**1,7**	0,000001
1440824_at			**1,7**	0,000001
1455226_at	Spnb1	**spectrin beta 1**	**1,7**	0,000066
1448301_s_at	Serpinb1a	**serine (or cysteine) peptidase inhibitor, clade B, member 1a**	**1,7**	0,000317
1454789_x_at	Prpf6	**PRP6 pre-mRNA splicing factor 6 homolog (yeast)**	**1,7**	0,000003
1431189_a_at	Fahd2a	**fumarylacetoacetate hydrolase domain containing 2A**	**1,7**	0,000000

1441009_at	4732491K20Rik	**RIKEN cDNA 4732491K20 gene**	**1,7**	0,000148
1458335_x_at	Urm1	**ubiquitin related modifier 1 homolog (S. cerevisiae)**	**1,7**	0,000029
1436099_at	AI836003	**expressed sequence AI836003**	**1,7**	0,000036
1418069_at	Apoc2	**apolipoprotein C-II**	**1,7**	0,000867
1422533_at	Cyp51	**cytochrome P450, family 51**	**1,7**	0,003016
1455912_x_at	Unc45a	**unc-45 homolog A (C. elegans)**	**1,7**	0,000000
1449687_at	D10Ertd610e	**DNA segment, Chr 10, ERATO Doi 610, expressed**	**1,6**	0,001949
1424014_at	2900092E17Rik	**RIKEN cDNA 2900092E17 gene**	**1,6**	0,000007
1429935_at	Lcn12	**lipocalin 12**	**1,6**	0,000005
1419972_at	Slc35a5	**solute carrier family 35, member A5**	**1,6**	0,000001
1445125_at	D8Ertd107e	**DNA segment, Chr 8, ERATO Doi 107, expressed**	**1,6**	0,000027
1416854_at	Slc34a2	**solute carrier family 34 (sodium phosphate), member 2**	**1,6**	0,015000
1446978_at			**1,6**	0,000011
1437140_at	4930412F15Rik	**RIKEN cDNA 4930412F15 gene**	**1,6**	0,000027
1436155_at	Nmnat2	**nicotinamide nucleotide adenylyltransferase 2**	**1,6**	0,000116
1427267_at	Tnrc18	**trinucleotide repeat containing 18**	**1,6**	0,000029
1438156_x_at	Cpt1a	**carnitine palmitoyltransferase 1a, liver**	**1,6**	0,001922
1455033_at	Fam102b	**family with sequence similarity 102, member B**	**1,6**	0,000012
1418113_at	Cyp2d10	**cytochrome P450, family 2, subfamily d, polypeptide 10**	**1,6**	0,001799
1437342_x_at	Pttg1ip	**pituitary tumor-transforming 1 interacting protein**	**1,6**	0,000007
1438588_at	Plagl1	**pleiomorphic adenoma gene-like 1**	**1,6**	0,000017
1418602_at	Cdh15	**cadherin 15**	**1,6**	0,000025
1441026_at	Parp4	**poly (ADP-ribose) polymerase family, member 4**	**1,6**	0,005460
1431688_at	LOC73899	**hypothetical LOC73899**	**1,6**	0,001015
1425514_at	Pik3r1	**phosphatidylinositol 3-kinase, regulatory subunit, polypeptide 1 (p85 alpha)**	**1,6**	0,000034
1456569_x_at	Gsn	**gelsolin**	**1,6**	0,000037
1425848_a_at	Dusp26	**dual specificity phosphatase 26 (putative)**	**1,6**	0,000089
1447227_at			**1,6**	0,000474
1439451_x_at	Gpr172b	**G protein-coupled receptor 172B**	**1,6**	0,000119
1445708_x_at	3110021A11Rik	**RIKEN cDNA 3110021A11 gene**	**1,6**	0,000029
1422320_x_at	Phxr5	**per-hexamer repeat gene 5**	**1,6**	0,000026
1427780_at	Defb35	**defensin beta 35**	**1,6**	0,000003
1459658_at	Mcm5	**minichromosome maintenance deficient 5, cell division cycle 46 (S. cerevisiae)**	**1,6**	0,000849
1423037_at	Aplnr	**apelin receptor**	**1,6**	0,000295
1452460_at	Ankrd26	**ankyrin repeat domain 26**	**1,6**	0,000190
1419918_at	Tmed7	**transmembrane emp24 protein transport domain containing 7**	**1,6**	0,000864
1456397_at	Cdh4	**cadherin 4**	**1,6**	0,000576
1445556_at			**1,6**	0,000049
1450361_at	Prop1	**paired like homeodomain factor 1**	**1,6**	0,000003
1417485_at	Ibsp	**integrin binding sialoprotein**	**1,6**	0,000083
1445566_at			**1,6**	0,000047

1439427_at	Cldn9	**claudin 9**	**1,6**	0,000003
1424386_at	Reep2	**receptor accessory protein 2**	**1,6**	0,000003
1433681_x_at	Capn3	**calpain 3**	**1,6**	0,000343
1433855_at	Abat	**4-aminobutyrate aminotransferase**	**1,6**	0,000174
1441910_x_at	Ccne1	**cyclin E1**	**1,6**	0,000003
1427970_at	Zfp689	**zinc finger protein 689**	**1,6**	0,000043
1422904_at	Fmo2	**flavin containing monooxygenase 2**	**1,6**	0,011499
1424722_at	1300017J02Rik	**RIKEN cDNA 1300017J02 gene**	**1,6**	0,000350
1435084_at	C730049O14Rik	**RIKEN cDNA C730049O14 gene**	**1,6**	0,001418
1429427_s_at	Tcf7l2	**transcription factor 7-like 2, T-cell specific, HMG-box**	**1,6**	0,003920
1419439_at	Stk22s1	**serine/threonine kinase 22 substrate 1**	**1,6**	0,000014
1440144_x_at	C330046E03	**hypothetical protein C330046E03**	**1,6**	0,000014
1445352_at			**1,6**	0,000045
1456844_at	Camk2d	**calcium/calmodulin-dependent protein kinase II, delta**	**1,6**	0,002838
1445662_x_at			**1,6**	0,000003
1436408_at	Rprml	**reprimo-like**	**1,6**	0,000887
1434831_a_at	Foxo3	**forkhead box O3**	**1,6**	0,000767
1444662_at	Pla2g4c	**phospholipase A2, group IVC (cytosolic, calcium-independent)**	**1,6**	0,000069
1424610_at	Trub2	**TruB pseudouridine (psi) synthase homolog 2 (E. coli)**	**1,6**	0,000275
1434426_at	Ncapd3	**non-SMC condensin II complex, subunit D3**	**1,6**	0,000974
1435964_a_at	Taok3	**TAO kinase 3**	**1,6**	0,000768
1418580_at	Rtp4	**receptor transporter protein 4**	**1,6**	0,005278
1443792_at	Tsga14	**testis specific gene A14**	**1,6**	0,000189
1437171_x_at	Gsn	**gelsolin**	**1,6**	0,000778
1433888_at	Atp2b2	**ATPase, Ca++ transporting, plasma membrane 2**	**1,6**	0,000003
1450255_at	Cdgap	**CDC42 GTPase-activating protein**	**1,6**	0,000141
1442241_at			**-1,6**	0,006507
1428234_at	Cpsf6	**cleavage and polyadenylation specific factor 6**	**-1,6**	0,002954
1447062_at			**-1,6**	0,000054
1434507_at	Npepl1	**aminopeptidase-like 1**	**-1,6**	0,000041
1434807_s_at	Mtx3	**metaxin 3**	**-1,6**	0,000388
1416835_s_at	Amd1	**S-adenosylmethionine decarboxylase 1**	**-1,6**	0,001681
1426232_at	BC024479	**cDNA sequence BC024479**	**-1,6**	0,008209
1433430_s_at	Cdc23	**CDC23 (cell division cycle 23, yeast, homolog)**	**-1,6**	0,000190
1441362_at			**-1,6**	0,000106
1451182_s_at	Ankrd54	**ankyrin repeat domain 54**	**-1,6**	0,000038
1456327_at			**-1,6**	0,005953
1428689_at	Tysnd1	**trypsin domain containing 1**	**-1,6**	0,000005
1449186_at	Bag4	**BCL2-associated athanogene 4**	**-1,6**	0,000052
1440715_s_at	Cdkn2aipnl	**CDKN2A interacting protein N-terminal like**	**-1,6**	0,005335
1418340_at	Fcer1g	**Fc receptor, IgE, high affinity I, gamma polypeptide**	**-1,6**	0,000389

1437410_at	Aldh2	**aldehyde dehydrogenase 2, mitochondrial**	**-1,6**	0,003684
1451794_at	Tmcc3	**transmembrane and coiled coil domains 3**	**-1,6**	0,005110
1427580_a_at	Rian	**RNA imprinted and accumulated in nucleus**	**-1,6**	0,002773
1439819_at	AU015263	**expressed sequence AU015263**	**-1,6**	0,008601
1430144_at	Avl9	**AVL9 homolog (S. cerevisiase)**	**-1,6**	0,004688
1442989_at			**-1,6**	0,000046
1443331_at			**-1,6**	0,000068
1418528_a_at	Dad1	**defender against cell death 1**	**-1,6**	0,001564
1452676_a_at	Pnpt1	**polyribonucleotide nucleotidyltransferase 1**	**-1,6**	0,012741
1420142_s_at	Pa2g4	**proliferation-associated 2G4**	**-1,6**	0,000783
1433174_a_at	5430440L12Rik	**RIKEN cDNA 5430440L12 gene**	**-1,6**	0,000295
1455863_at	Spata5l1	**spermatogenesis associated 5-like 1**	**-1,6**	0,000953
1417716_at	Got2	**glutamate oxaloacetate transaminase 2, mitochondrial**	**-1,6**	0,000002
1428707_at	Ptms	**parathymosin**	**-1,6**	0,000003
1423795_at	Sfpq	**splicing factor proline/glutamine rich (polypyrimidine tract binding protein associated)**	**-1,6**	0,003932
1454842_a_at	B3galnt2	**UDP-GalNAc:betaGlcNAc beta 1,3-galactosaminyltransferase, polypeptide 2**	**-1,6**	0,009871
1439797_at	Ppard	**peroxisome proliferator activator receptor delta**	**-1,6**	0,001178
1460678_at	Klhdc2	**kelch domain containing 2**	**-1,6**	0,000825
1429725_at	Zfhx3	**zinc finger homeobox 3**	**-1,6**	0,000181
1434493_at	1810022K09Rik	**RIKEN cDNA 1810022K09 gene**	**-1,6**	0,010362
1451127_at	AW146242	**expressed sequence AW146242**	**-1,6**	0,000286
1425204_s_at	Ddx19a	**DEAD (Asp-Glu-Ala-Asp) box polypeptide 19a**	**-1,6**	0,000078
1429180_at	Gmpr2	**guanosine monophosphate reductase 2**	**-1,6**	0,010788
1425551_at	Hip1r	**huntingtin interacting protein 1 related**	**-1,6**	0,000366
1444478_at	Appl1	**adaptor protein, phosphotyrosine interaction, PH domain and leucine zipper containing 1**	**-1,6**	0,002613
1422779_at	Smpd3	**sphingomyelin phosphodiesterase 3, neutral**	**-1,6**	0,000134
1418596_at	Fgfr4	**fibroblast growth factor receptor 4**	**-1,6**	0,000096
1416487_a_at	Yap1	**yes-associated protein 1**	**-1,6**	0,000094
1456386_at			**-1,6**	0,000602
1418253_a_at	Hspa4l	**heat shock protein 4 like**	**-1,6**	0,000243
1430415_at	Phf6	**PHD finger protein 6**	**-1,6**	0,001624
1444459_at			**-1,6**	0,002404
1458031_at			**-1,6**	0,000008
1431374_at	6330407A03Rik	**RIKEN cDNA 6330407A03 gene**	**-1,6**	0,000642
1448140_at	Ciapin1	**cytokine induced apoptosis inhibitor 1**	**-1,6**	0,000431
1428429_at	Rgmb	**RGM domain family, member B**	**-1,6**	0,000709
1440234_at	1810012P15Rik	**RIKEN cDNA 1810012P15 gene**	**-1,6**	0,000031
1425315_at	Dock7	**dedicator of cytokinesis 7**	**-1,6**	0,000952
1446447_at			**-1,6**	0,000056
1424923_at	Serpina3g	**serine (or cysteine) peptidase inhibitor, clade A, member 3G**	**-1,6**	0,013360
1449157_at	Nr2c1	**nuclear receptor subfamily 2, group C, member 1**	**-1,6**	0,000048

1448668_a_at	Irak1	**interleukin-1 receptor-associated kinase 1**	**-1,6**	0,000017
1423617_at	Pdf	**peptide deformylase (mitochondrial)**	**-1,6**	0,000105
1424197_s_at	Fance	**Fanconi anemia, complementation group E**	**-1,6**	0,000018
1435325_at	Usp46	**ubiquitin specific peptidase 46**	**-1,6**	0,000334
1456250_x_at	Tgfbi	**transforming growth factor, beta induced**	**-1,6**	0,001768
1441112_at	LOC667118	**similar to Zinc finger BED domain containing protein 4**	**-1,6**	0,006783
1443649_at			**-1,6**	0,001631
1428749_at	Dmxl2	**Dmx-like 2**	**-1,6**	0,001329
1453251_at	Dhx30	**DEAH (Asp-Glu-Ala-His) box polypeptide 30**	**-1,6**	0,001845
1443628_at			**-1,6**	0,000866
1426025_s_at	Laptm5	**lysosomal-associated protein transmembrane 5**	**-1,6**	0,000056
1423053_at	Arf4	**ADP-ribosylation factor 4**	**-1,6**	0,000010
1432384_a_at	Mettl6	**methyltransferase like 6**	**-1,6**	0,002797
1439201_at	Usp14	**ubiquitin specific peptidase 14**	**-1,6**	0,000053
1440068_at			**-1,6**	0,000192
1417311_at	Crip2	**cysteine rich protein 2**	**-1,6**	0,004015
1451091_at	Txndc5	**thioredoxin domain containing 5**	**-1,6**	0,000150
1458812_at			**-1,6**	0,000288
1438725_at	Med13	**mediator complex subunit 13**	**-1,6**	0,000222
1448585_at	Gtf2h4	**general transcription factor II H, polypeptide 4**	**-1,6**	0,000156
1429368_at	Lrig3	**leucine-rich repeats and immunoglobulin-like domains 3**	**-1,6**	0,000424
1440690_at			**-1,6**	0,001323
1418440_at	Col8a1	**collagen, type VIII, alpha 1**	**-1,6**	0,006082
1452381_at	Creb3l2	**cAMP responsive element binding protein 3-like 2**	**-1,6**	0,009036
1453228_at	Stx11	**syntaxin 11**	**-1,6**	0,000271
1456927_at	Mast2	**microtubule associated serine/threonine kinase 2**	**-1,6**	0,004584
1456933_at			**-1,6**	0,003023
1439622_at	Rassf4	**Ras association (RalGDS/AF-6) domain family member 4**	**-1,6**	0,000859
1429504_at	Rnpc3	**RNA-binding region (RNP1, RRM) containing 3**	**-1,6**	0,011502
1457641_at			**-1,6**	0,000869
1415888_at	Hdgf	**hepatoma-derived growth factor**	**-1,6**	0,000281
1420817_at	Ywhag	**tyrosine 3-monooxygenase/tryptophan 5-monooxygenase activation protein, gamma polypeptide**	**-1,6**	0,000073
1441033_at	Tmtc2	**transmembrane and tetratricopeptide repeat containing 2**	**-1,6**	0,000172
1452182_at	Galnt2	**UDP-N-acetyl-alpha-D-galactosamine:polypeptide N-acetylgalactosaminyltransferase 2**	**-1,6**	0,000001
1423345_at	Degs1	**degenerative spermatocyte homolog 1 (Drosophila)**	**-1,6**	0,000035
1426575_at	Sgms1	**sphingomyelin synthase 1**	**-1,6**	0,000448
1442511_at	Ipo7	**importin 7**	**-1,6**	0,015435
1430996_at	Etnk1	**ethanolamine kinase 1**	**-1,6**	0,001020
1451391_at	2700050L05Rik	**RIKEN cDNA 2700050L05 gene**	**-1,6**	0,000963
1452869_at	Prpf38b	**PRP38 pre-mRNA processing factor 38 (yeast) domain containing B**	**-1,6**	0,002673
1430108_at	9030622M22Rik	**RIKEN cDNA 9030622M22 gene**	**-1,6**	0,000041

1439974_at	Fkbp15	**FK506 binding protein 15**	**-1,6**	0,003129
1432207_a_at	Toe1	**target of EGR1, member 1 (nuclear)**	**-1,6**	0,000064
1423982_at	Fusip1	**FUS interacting protein (serine-arginine rich) 1**	**-1,6**	0,005725
1458969_at	AU019559	**expressed sequence AU019559**	**-1,6**	0,000659
1450264_a_at	Chka	**choline kinase alpha**	**-1,6**	0,000606
1423321_at	Myadm	**myeloid-associated differentiation marker**	**-1,6**	0,000014
1418925_at	Celsr1	**cadherin, EGF LAG seven-pass G-type receptor 1 (flamingo homolog, Drosophila)**	**-1,6**	0,000053
1438442_at	5730470L24Rik	**RIKEN cDNA 5730470L24 gene**	**-1,6**	0,000385
1458737_at	C77097	**expressed sequence C77097**	**-1,6**	0,004714
1424232_a_at	Ftsjd1	**FtsJ methyltransferase domain containing 1**	**-1,6**	0,000011
1448794_s_at	Dnajc2	**DnaJ (Hsp40) homolog, subfamily C, member 2**	**-1,6**	0,006114
1438454_at	B430203M17Rik	**RIKEN cDNA B430203M17 gene**	**-1,6**	0,001666
1429586_at	4930558N01Rik	**RIKEN cDNA 4930558N01 gene**	**-1,6**	0,000029
1443156_at			**-1,6**	0,002082
1442494_at	Ubr2	**ubiquitin protein ligase E3 component n-recognin 2**	**-1,6**	0,002266
1418222_at	2610024G14Rik	**RIKEN cDNA 2610024G14 gene**	**-1,6**	0,000299
1441253_at			**-1,6**	0,013109
1448500_a_at	Lime1	**Lck interacting transmembrane adaptor 1**	**-1,6**	0,008299
1443068_at	D130084N16Rik	**RIKEN cDNA D130084N16 gene**	**-1,6**	0,004081
1452020_a_at	Siva1	**SIVA1, apoptosis-inducing factor**	**-1,6**	0,001373
1417602_at	Per2	**period homolog 2 (Drosophila)**	**-1,6**	0,000205
1429359_s_at	Rbpms	**RNA binding protein gene with multiple splicing**	**-1,6**	0,015601
1415689_s_at	Zkscan3	**zinc finger with KRAB and SCAN domains 3**	**-1,6**	0,000827
1439787_at	P2rx7	**purinergic receptor P2X, ligand-gated ion channel, 7**	**-1,6**	0,000070
1425424_at	MGC7817	**hypothetical protein LOC620031**	**-1,6**	0,000408
1450480_a_at	Grk6	**G protein-coupled receptor kinase 6**	**-1,6**	0,000083
1458602_at	Bbx	**bobby sox homolog (Drosophila)**	**-1,6**	0,000106
1439953_at	Pmm2	**phosphomannomutase 2**	**-1,6**	0,000711
1425348_a_at	Srprb	**signal recognition particle receptor, B subunit**	**-1,6**	0,000077
1417807_at	Ufsp1	**UFM1-specific peptidase 1**	**-1,6**	0,000070
1460548_a_at	Eral1	**Era (G-protein)-like 1 (E. coli)**	**-1,6**	0,000297
1456413_at	Pde4dip	**phosphodiesterase 4D interacting protein (myomegalin)**	**-1,6**	0,002884
1456476_at	Atxn2l	**ataxin 2-like**	**-1,6**	0,000106
1456897_at			**-1,6**	0,000874
1429963_at	Mapk6	**mitogen-activated protein kinase 6**	**-1,6**	0,000619
1453051_at	Zkscan1	**zinc finger with KRAB and SCAN domains 1**	**-1,6**	0,000001
1440635_at	Palld	**palladin, cytoskeletal associated protein**	**-1,6**	0,002672
1418475_at	Scnn1b	**sodium channel, nonvoltage-gated 1 beta**	**-1,6**	0,003407
1430992_s_at	Cisd2	**CDGSH iron sulfur domain 2**	**-1,6**	0,000044
1451591_a_at	Efnb1	**ephrin B1**	**-1,6**	0,000594
1428100_at	Sfrs1	**splicing factor, arginine/serine-rich 1 (ASF/SF2)**	**-1,6**	0,001974

1448691_at	Ubqln4	**ubiquilin 4**	**-1,6**	0,000001
1443630_at			**-1,6**	0,000308
1447465_at			**-1,6**	0,000011
1426764_at	Oaz2	**ornithine decarboxylase antizyme 2**	**-1,6**	0,000100
1455135_at	Ccdc9	**coiled-coil domain containing 9**	**-1,6**	0,000093
1443162_at			**-1,6**	0,000350
1419247_at	Rgs2	**regulator of G-protein signaling 2**	**-1,6**	0,000247
1440009_at	Olfr78	**olfactory receptor 78**	**-1,6**	0,010247
1420866_at	Zfp161	**zinc finger protein 161**	**-1,6**	0,000075
1460366_at	Eml3	**echinoderm microtubule associated protein like 3**	**-1,6**	0,000046
1426744_at	Srebf2	**sterol regulatory element binding factor 2**	**-1,6**	0,000002
1451927_a_at	Mapk14	**mitogen-activated protein kinase 14**	**-1,6**	0,004047
1436305_at	Rnf217	**ring finger protein 217**	**-1,6**	0,000080
1443148_at			**-1,6**	0,001245
1436665_a_at	Ltbp4	**latent transforming growth factor beta binding protein 4**	**-1,6**	0,001262
1425118_at	Spire2	**spire homolog 2 (Drosophila)**	**-1,6**	0,001311
1451667_at	Fam20b	**family with sequence similarity 20, member B**	**-1,6**	0,003868
1423879_at	D030056L22Rik	**RIKEN cDNA D030056L22 gene**	**-1,6**	0,000213
1455208_at	Pex19	**peroxisomal biogenesis factor 19**	**-1,6**	0,000053
1435923_at	Ado	**2-aminoethanethiol (cysteamine) dioxygenase**	**-1,6**	0,000536
1423450_a_at	Hs3st1	**heparan sulfate (glucosamine) 3-O-sulfotransferase 1**	**-1,6**	0,000054
1417254_at	Spata5	**spermatogenesis associated 5**	**-1,6**	0,000965
1455439_a_at	Lgals1	**lectin, galactose binding, soluble 1**	**-1,6**	0,007017
1419403_at	BC017612	**cDNA sequence BC017612**	**-1,6**	0,000869
1425845_a_at	Shoc2	**soc-2 (suppressor of clear) homolog (C. elegans)**	**-1,6**	0,000008
1416657_at	Akt1	**thymoma viral proto-oncogene 1**	**-1,6**	0,000003
1426790_at	Ssrp1	**structure specific recognition protein 1**	**-1,6**	0,000492
1434282_at	Ibtk	**inhibitor of Bruton agammaglobulinemia tyrosine kinase**	**-1,6**	0,000075
1442704_at			**-1,6**	0,001341
1434133_s_at	Wdr42a	**WD repeat domain 42A**	**-1,6**	0,000029
1430175_at	4930588G05Rik	**RIKEN cDNA 4930588G05 gene**	**-1,6**	0,001061
1451538_at	Sox9	**SRY-box containing gene 9**	**-1,6**	0,001053
1435916_at	Zfp84	**zinc finger protein 84**	**-1,6**	0,002291
1433506_at	Lrrc8d	**leucine rich repeat containing 8D**	**-1,6**	0,000309
1451099_at	Mbc2	**membrane bound C2 domain containing protein**	**-1,6**	0,000074
1443593_at			**-1,6**	0,001363
1434404_at	Fam73a	**family with sequence similarity 73, member A**	**-1,6**	0,005473
1449122_at	Ubxn2b	**UBX domain protein 2B**	**-1,6**	0,001017
1427417_at	Scml4	**sex comb on midleg-like 4 (Drosophila)**	**-1,6**	0,001056
1454743_at	Nup205	**nucleoporin 205**	**-1,6**	0,004080
1445885_at	Ube2d2	**ubiquitin-conjugating enzyme E2D 2**	**-1,6**	0,001509

1459648_at			**-1,6**	0,000043
1422793_at	Pafah1b2	**platelet-activating factor acetylhydrolase, isoform 1b, alpha2 subunit**	**-1,6**	0,000651
1458205_at			**-1,6**	0,001652
1428174_x_at	Khsrp	**KH-type splicing regulatory protein**	**-1,6**	0,000011
1451644_a_at	H2-gs10	**MHC class I like protein GS10**	**-1,6**	0,004917
1451514_at	Usp43	**ubiquitin specific peptidase 43**	**-1,6**	0,000005
1421784_a_at	Efna4	**ephrin A4**	**-1,6**	0,014561
1422551_at	Zkscan3	**zinc finger with KRAB and SCAN domains 3**	**-1,6**	0,000124
1447575_at			**-1,6**	0,000038
1426945_at	Ipo5	**importin 5**	**-1,6**	0,000623
1458974_at			**-1,6**	0,000017
1429464_at	Prkaa2	**protein kinase, AMP-activated, alpha 2 catalytic subunit**	**-1,6**	0,005457
1428168_at	Mpzl1	**myelin protein zero-like 1**	**-1,6**	0,001206
1416548_at	Slc35b4	**solute carrier family 35, member B4**	**-1,6**	0,000021
1458290_at			**-1,6**	0,002360
1456209_x_at			**-1,6**	0,001079
1437491_at	Bicd2	**bicaudal D homolog 2 (Drosophila)**	**-1,6**	0,000012
1427153_at	Bckdhb	**branched chain ketoacid dehydrogenase E1, beta polypeptide**	**-1,6**	0,008209
1418284_at	Vps72	**vacuolar protein sorting 72 (yeast)**	**-1,6**	0,000001
1422532_at	Xpc	**xeroderma pigmentosum, complementation group C**	**-1,6**	0,000014
1441732_at			**-1,6**	0,009311
1448623_at	Tmem123	**transmembrane protein 123**	**-1,6**	0,006826
1430515_s_at	Aasdhppt	**aminoadipate-semialdehyde dehydrogenase-phosphopantetheinyl transferase**	**-1,6**	0,006239
1457334_at	C130057M05Rik	**RIKEN cDNA C130057M05 gene**	**-1,6**	0,000352
1424078_s_at	Pex6	**peroxisomal biogenesis factor 6**	**-1,6**	0,000184
1449504_at	Kpna1	**karyopherin (importin) alpha 1**	**-1,6**	0,000323
1434680_at	Plekhg3	**pleckstrin homology domain containing, family G (with RhoGef domain) member 3**	**-1,6**	0,000003
1450519_a_at	Prkaca	**protein kinase, cAMP dependent, catalytic, alpha**	**-1,6**	0,000064
1427476_a_at	Trim32	**tripartite motif-containing 32**	**-1,6**	0,000190
1443905_at			**-1,6**	0,002877
1429846_at	9030411K21Rik	**RIKEN cDNA 9030411K21 gene**	**-1,6**	0,000719
1426781_at	Tyw1	**tRNA-yW synthesizing protein 1 homolog (S. cerevisiae)**	**-1,6**	0,000242
1421908_a_at	Tcf12	**transcription factor 12**	**-1,6**	0,000532
1444827_at			**-1,6**	0,001417
1455065_x_at	Gnpda1	**glucosamine-6-phosphate deaminase 1**	**-1,6**	0,001509
1417551_at	Cln3	**ceroid lipofuscinosis, neuronal 3, juvenile (Batten, Spielmeyer-Vogt disease)**	**-1,6**	0,000472
1448392_at	Sparc	**secreted acidic cysteine rich glycoprotein**	**-1,6**	0,012991
1419072_at	Gstm7	**glutathione S-transferase, mu 7**	**-1,6**	0,000164
1448446_at	Deaf1	**deformed epidermal autoregulatory factor 1 (Drosophila)**	**-1,6**	0,000021
1449523_at	Bcl7c	**B-cell CLL/lymphoma 7C**	**-1,6**	0,000001
1418118_at	Slc22a1	**solute carrier family 22 (organic cation transporter), member 1**	**-1,6**	0,000162

1426518_at	Tubgcp5	**tubulin, gamma complex associated protein 5**	**-1,6**	0,000229
1417753_at	Pkd2	**polycystic kidney disease 2**	**-1,6**	0,002062
1426548_a_at	Atpbd4	**ATP binding domain 4**	**-1,6**	0,012039
1416075_at	Sav1	**salvador homolog 1 (Drosophila)**	**-1,6**	0,000002
1436007_a_at	Thumpd1	**THUMP domain containing 1**	**-1,6**	0,001071
1438419_at	Rbm16	**RNA binding motif protein 16**	**-1,6**	0,001261
1449947_s_at	Zfhx3	**zinc finger homeobox 3**	**-1,6**	0,013826
1452831_s_at	Ppat	**phosphoribosyl pyrophosphate amidotransferase**	**-1,6**	0,013337
1421052_a_at	Sms	**spermine synthase**	**-1,6**	0,000663
1452099_at	AA408296	**expressed sequence AA408296**	**-1,6**	0,001646
1444705_at			**-1,6**	0,000240
1417606_a_at	Calr	**calreticulin**	**-1,6**	0,000014
1424201_a_at	Seh1l	**SEH1-like (S. cerevisiae**	**-1,6**	0,000140
1422993_s_at	Refbp2	**RNA and export factor binding protein 2**	**-1,6**	0,009750
1416235_at	Lrrc59	**leucine rich repeat containing 59**	**-1,6**	0,000001
1452191_at	Prcp	**prolylcarboxypeptidase (angiotensinase C)**	**-1,6**	0,000050
1440408_at	B830008J18Rik	**RIKEN cDNA B830008J18 gene**	**-1,6**	0,001675
1455987_at	Sec61a1	**Sec61 alpha 1 subunit (S. cerevisiae)**	**-1,6**	0,000751
1444530_at			**-1,6**	0,000558
1423765_at	Athl1	**ATH1, acid trehalase-like 1 (yeast)**	**-1,6**	0,000069
1449117_at	Jund	**Jun proto-oncogene related gene d**	**-1,6**	0,000422
1451168_a_at	Arhgdia	**Rho GDP dissociation inhibitor (GDI) alpha**	**-1,6**	0,000044
1460692_at	Ehmt2	**euchromatic histone lysine N-methyltransferase 2**	**-1,6**	0,000002
1452045_at	Zfp281	**zinc finger protein 281**	**-1,6**	0,003080
1424660_s_at	Crtc2	**CREB regulated transcription coactivator 2**	**-1,6**	0,000003
1446929_at	D130062J21Rik	**RIKEN cDNA D130062J21 gene**	**-1,6**	0,004314
1439349_at	Sbno2	**strawberry notch homolog 2 (Drosophila)**	**-1,6**	0,000049
1451360_at	Ergic2	**ERGIC and golgi 2**	**-1,6**	0,000078
1439097_at			**-1,6**	0,000917
1416240_at	Psmb7	**proteasome (prosome, macropain) subunit, beta type 7**	**-1,6**	0,000219
1422493_at	Cpox	**coproporphyrinogen oxidase**	**-1,6**	0,000047
1448620_at	Fcgr3	**Fc receptor, IgG, low affinity III**	**-1,6**	0,012327
1457062_at	1700081L11Rik	**RIKEN cDNA 1700081L11 gene**	**-1,6**	0,000270
1416397_at	Mesdc1	**mesoderm development candidate 1**	**-1,6**	0,000055
1421832_at	Twsg1	**twisted gastrulation homolog 1 (Drosophila)**	**-1,6**	0,000081
1455841_s_at	Grwd1	**glutamate-rich WD repeat containing 1**	**-1,6**	0,000290
1451221_at	BC018507	**cDNA sequence BC018507**	**-1,6**	0,000980
1418644_a_at	Stk11	**serine/threonine kinase 11**	**-1,6**	0,000007
1426627_at	Map3k7	**mitogen-activated protein kinase kinase kinase 7**	**-1,6**	0,000332
1443109_at			**-1,6**	0,003648
1425592_at	Tnpo2	**transportin 2 (importin 3, karyopherin beta 2b)**	**-1,6**	0,000047

1427490_at	Abcb7	**ATP-binding cassette, sub-family B (MDR/TAP), member 7**	**-1,6**	0,001867
1435514_at	Lztfl1	**leucine zipper transcription factor-like 1**	**-1,6**	0,000522
1444185_at			**-1,6**	0,000027
1457154_at			**-1,6**	0,004512
1450570_a_at	Cd19	**CD19 antigen**	**-1,6**	0,002726
1446860_at			**-1,6**	0,000716
1450269_a_at	Pfkl	**phosphofructokinase, liver, B-type**	**-1,6**	0,001577
1429984_at	5730455O13Rik	**RIKEN cDNA 5730455O13 gene**	**-1,6**	0,000069
1457500_at	Depdc5	**DEP domain containing 5**	**-1,6**	0,008561
1438270_at	AI846148	**expressed sequence AI846148**	**-1,6**	0,000090
1459237_at			**-1,6**	0,001505
1426118_a_at	Tomm40	**translocase of outer mitochondrial membrane 40 homolog (yeast)**	**-1,6**	0,000000
1416637_at	Slc4a2	**solute carrier family 4 (anion exchanger), member 2**	**-1,6**	0,001438
1445830_at	Ctnnd1	**catenin (cadherin associated protein), delta 1**	**-1,6**	0,000297
1456973_at			**-1,6**	0,006071
1456775_at	Ints8	**integrator complex subunit 8**	**-1,6**	0,000013
1417740_at	Cdc37l1	**cell division cycle 37 homolog (S. cerevisiae)-like 1**	**-1,6**	0,000014
1427987_at	Safb2	**scaffold attachment factor B2**	**-1,6**	0,000025
1435965_at	Cnot3	**CCR4-NOT transcription complex, subunit 3**	**-1,6**	0,000443
1419330_a_at	Gpa33	**glycoprotein A33 (transmembrane)**	**-1,6**	0,000202
1418763_at	Nit2	**nitrilase family, member 2**	**-1,6**	0,001825
1447240_at			**-1,6**	0,000594
1428881_at	Klc1	**kinesin light chain 1**	**-1,6**	0,000741
1457834_at	Yy1	**YY1 transcription factor**	**-1,6**	0,000111
1419054_a_at	Ptpn21	**protein tyrosine phosphatase, non-receptor type 21**	**-1,6**	0,000012
1436039_at	Cmah	**cytidine monophospho-N-acetylneuraminic acid hydroxylase**	**-1,6**	0,004274
1449976_a_at	Gpr35	**G protein-coupled receptor 35**	**-1,6**	0,001441
1436660_at	Rrbp1	**ribosome binding protein 1**	**-1,6**	0,000069
1416750_at	Sigmar1	**sigma non-opioid intracellular receptor 1**	**-1,6**	0,000003
1450015_x_at	Sgpp1	**sphingosine-1-phosphate phosphatase 1**	**-1,6**	0,000157
1452989_at	2900009J20Rik	**RIKEN cDNA 2900009J20 gene**	**-1,6**	0,000006
1434558_at	Wdr47	**WD repeat domain 47**	**-1,6**	0,000427
1438762_at			**-1,6**	0,001358
1426574_a_at	Add3	**adducin 3 (gamma)**	**-1,6**	0,000523
1423358_at	Ece2	**endothelin converting enzyme 2**	**-1,6**	0,000045
1453993_a_at	Bnip2	**BCL2/adenovirus E1B interacting protein 2**	**-1,6**	0,003541
1420917_at	Prpf40a	**PRP40 pre-mRNA processing factor 40 homolog A (yeast)**	**-1,6**	0,003315
1423446_at	Dapk3	**death-associated protein kinase 3**	**-1,6**	0,000111
1436372_a_at	Pdxdc1	**pyridoxal-dependent decarboxylase domain containing 1**	**-1,6**	0,000000
1433804_at	Jak1	**Janus kinase 1**	**-1,6**	0,001153
1437532_at	Rnf216	**ring finger protein 216**	**-1,6**	0,000014

1425660_at	Btbd3	**BTB (POZ) domain containing 3**	**-1,6**	0,000098
1458934_at	D5Ertd505e	**DNA segment, Chr 5, ERATO Doi 505 , expressed**	**-1,6**	0,000130
1452500_at			**-1,6**	0,000413
1418565_at	Serbp1	**serpine1 mRNA binding protein 1**	**-1,6**	0,000069
1421889_a_at	Aplp2	**amyloid beta (A4) precursor-like protein 2**	**-1,6**	0,000002
1416032_at	Tmem109	**transmembrane protein 109**	**-1,6**	0,000005
1420930_s_at	Ctnnal1	**catenin (cadherin associated protein), alpha-like 1**	**-1,6**	0,000221
1424056_at	Usp48	**ubiquitin specific peptidase 48**	**-1,6**	0,000033
1424982_a_at	2700078E11Rik	**RIKEN cDNA 2700078E11 gene**	**-1,6**	0,000015
1416589_at	Sparc	**secreted acidic cysteine rich glycoprotein**	**-1,6**	0,003182
1420684_at	Acox3	**acyl-Coenzyme A oxidase 3, pristanoyl**	**-1,6**	0,000980
1450869_at	Fgf1	**fibroblast growth factor 1**	**-1,6**	0,004437
1457970_at	Actr1a	**ARP1 actin-related protein 1 homolog A, centractin alpha (yeast)**	**-1,6**	0,000068
1460724_at	Ap2a1	**adaptor protein complex AP-2, alpha 1 subunit**	**-1,6**	0,000011
1447016_at	Tbc1d1	**TBC1 domain family, member 1**	**-1,6**	0,011490
1430103_at	9030607L20Rik	**RIKEN cDNA 9030607L20 gene**	**-1,6**	0,000139
1429847_a_at	4833418A01Rik	**RIKEN cDNA 4833418A01 gene**	**-1,6**	0,000131
1419550_a_at	Stk39	**serine/threonine kinase 39, STE20/SPS1 homolog (yeast)**	**-1,6**	0,000124
1438900_at	Sacm1l	**SAC1 (suppressor of actin mutations 1, homolog)-like (S. cerevisiae)**	**-1,6**	0,000024
1419573_a_at	Lgals1	**lectin, galactose binding, soluble 1**	**-1,6**	0,004722
1417568_at	Ncald	**neurocalcin delta**	**-1,6**	0,000054
1417295_at	Mta1	**metastasis associated 1**	**-1,6**	0,000002
1457304_at	D13Ertd787e	**DNA segment, Chr 13, ERATO Doi 787, expressed**	**-1,6**	0,000663
1425680_a_at	Btrc	**beta-transducin repeat containing protein**	**-1,6**	0,001617
1415899_at	Junb	**Jun-B oncogene**	**-1,6**	0,000475
1448604_at	Uck2	**uridine-cytidine kinase 2**	**-1,6**	0,000137
1431752_a_at	Urm1	**ubiquitin related modifier 1 homolog (S. cerevisiae)**	**-1,6**	0,000000
1437476_at	Rrm2b	**ribonucleotide reductase M2 B (TP53 inducible)**	**-1,6**	0,000047
1440305_at			**-1,6**	0,006857
1418982_at	Cebpa	**CCAAT/enhancer binding protein (C/EBP), alpha**	**-1,6**	0,000543
1426293_at	Zfp790	**zinc finger protein 790**	**-1,6**	0,000058
1448398_s_at	Rpl22	**ribosomal protein L22**	**-1,6**	0,000077
1416172_at	Pes1	**pescadillo homolog 1, containing BRCT domain (zebrafish)**	**-1,6**	0,002955
1456880_at			**-1,6**	0,000453
1456103_at	Pml	**promyelocytic leukemia**	**-1,6**	0,000070
1446735_at	Itsn2	**intersectin 2**	**-1,6**	0,000339
1452826_s_at	Fbxl20	**F-box and leucine-rich repeat protein 20**	**-1,6**	0,000029
1439639_at			**-1,6**	0,000795
1430161_at	Dlst	**dihydrolipoamide S-succinyltransferase (E2 component of 2-oxo-glutarate complex)**	**-1,6**	0,000007
1441707_at	Psma3	**proteasome (prosome, macropain) subunit, alpha type 3**	**-1,6**	0,001559
1426384_a_at	Ywhae	**tyrosine 3-monooxygenase/tryptophan 5-monooxygenase activation protein, epsilon polypeptide**	**-1,6**	0,000002

1457489_at			**-1,6**	0,000753
1442622_at			**-1,6**	0,000348
1436917_s_at	D2Bwg1335e	**DNA segment, Chr 2, Brigham & Women's Genetics 1335 expressed**	**-1,6**	0,014286
1453589_a_at	6820431F20Rik	**RIKEN cDNA 6820431F20 gene**	**-1,6**	0,002311
1453380_a_at	Xrcc6bp1	**XRCC6 binding protein 1**	**-1,6**	0,000067
1458508_at	Matr3	**matrin 3**	**-1,6**	0,000577
1445438_at	Ddhd1	**DDHD domain containing 1**	**-1,6**	0,000413
1423045_at	Ncbp2	**nuclear cap binding protein subunit 2**	**-1,6**	0,005012
1431680_a_at	Ptprk	**protein tyrosine phosphatase, receptor type, K**	**-1,6**	0,000127
1425650_at	Tle4	**transducin-like enhancer of split 4, homolog of Drosophila E(spl)**	**-1,6**	0,000057
1416386_a_at	M6pr	**mannose-6-phosphate receptor, cation dependent**	**-1,6**	0,000004
1444409_at	Rph3al	**rabphilin 3A-like (without C2 domains)**	**-1,6**	0,005737
1433932_x_at	C030046I01Rik	**RIKEN cDNA C030046I01 gene**	**-1,6**	0,000314
1442163_at			**-1,6**	0,000232
1439342_at	Clpx	**caseinolytic peptidase X (E.coli)**	**-1,6**	0,004189
1452406_x_at	Erdr1	**erythroid differentiation regulator 1**	**-1,6**	0,010460
1441351_at			**-1,6**	0,000444
1441372_at	5930405F01Rik	**RIKEN cDNA 5930405F01 gene**	**-1,6**	0,007282
1430538_at	2210013O21Rik	**RIKEN cDNA 2210013O21 gene**	**-1,6**	0,000028
1425919_at	Ndufa12	**NADH dehydrogenase (ubiquinone) 1 alpha subcomplex, 12**	**-1,6**	0,001849
1422719_s_at	Nup50	**nucleoporin 50**	**-1,6**	0,000147
1424614_at	Frag1	**FGF receptor activating protein 1**	**-1,6**	0,000005
1425805_a_at	Usp12	**ubiquitin specific peptidase 12**	**-1,6**	0,000330
1459144_at			**-1,6**	0,000573
1417187_at	Ube2k	**ubiquitin-conjugating enzyme E2K (UBC1 homolog, yeast)**	**-1,6**	0,000090
1442244_at	Inadl	**InaD-like (Drosophila)**	**-1,6**	0,000061
1423229_at	Inpp5e	**inositol polyphosphate-5-phosphatase E**	**-1,6**	0,000005
1418133_at	Bcl3	**B-cell leukemia/lymphoma 3**	**-1,6**	0,000163
1439125_at			**-1,6**	0,000315
1447973_at	Trip11	**thyroid hormone receptor interactor 11**	**-1,6**	0,000046
1443896_at	Tbc1d5	**TBC1 domain family, member 5**	**-1,6**	0,000817
1439467_at			**-1,6**	0,003673
1435695_a_at	Ggct	**gamma-glutamyl cyclotransferase**	**-1,6**	0,000102
1456388_at	Atp11a	**ATPase, class VI, type 11A**	**-1,6**	0,000083
1455591_at	Zfp618	**zinc fingerprotein 618**	**-1,6**	0,004499
1425326_at	Acly	**ATP citrate lyase**	**-1,6**	0,000080
1448569_at	Mlec	**malectin**	**-1,6**	0,000018
1441769_at			**-1,6**	0,000676
1443535_at	Rabgap1	**RAB GTPase activating protein 1**	**-1,6**	0,000641
1438345_at			**-1,6**	0,000016
1434196_at	Dnaja4	**DnaJ (Hsp40) homolog, subfamily A, member 4**	**-1,6**	0,002299

1435353_a_at	Sfi1	**Sfi1 homolog, spindle assembly associated (yeast)**	**-1,6**	0,000370
1434499_a_at	Ldhb	**lactate dehydrogenase B**	**-1,6**	0,000298
1415770_at	Wdr6	**WD repeat domain 6**	**-1,6**	0,001511
1448883_at	Lgmn	**legumain**	**-1,6**	0,000034
1417188_s_at	Ube2k	**ubiquitin-conjugating enzyme E2K (UBC1 homolog, yeast)**	**-1,6**	0,001320
1426365_at	2810403A07Rik	**RIKEN cDNA 2810403A07 gene**	**-1,6**	0,001233
1428656_at	Rnasen	**ribonuclease III, nuclear**	**-1,6**	0,000014
1423741_at	Rbm10	**RNA binding motif protein 10**	**-1,6**	0,000059
1456865_x_at	Rrs1	**RRS1 ribosome biogenesis regulator homolog (S. cerevisiae)**	**-1,6**	0,003150
1444343_at	A130064L14Rik	**RIKEN cDNA A130064L14 gene**	**-1,6**	0,003213
1437182_at	Dido1	**death inducer-obliterator 1**	**-1,6**	0,001909
1423584_at	Polr2b	**polymerase (RNA) II (DNA directed) polypeptide B**	**-1,6**	0,009277
1432262_at	Fam63a	**family with sequence similarity 63, member A**	**-1,6**	0,000079
1421148_a_at	Tial1	**Tia1 cytotoxic granule-associated RNA binding protein-like 1**	**-1,6**	0,000095
1460304_a_at	Ubtf	**upstream binding transcription factor, RNA polymerase I**	**-1,6**	0,000021
1435242_at	Pds5b	**PDS5, regulator of cohesion maintenance, homolog B (S. cerevisiae)**	**-1,6**	0,001820
1437184_at	Guf1	**GUF1 GTPase homolog (S. cerevisiae)**	**-1,6**	0,005107
1436779_at	Cybb	**cytochrome b-245, beta polypeptide**	**-1,6**	0,000232
1442731_at	Pds5a	**PDS5, regulator of cohesion maintenance, homolog A (S. cerevisiae)**	**-1,6**	0,004188
1450407_a_at	Anp32a	**acidic (leucine-rich) nuclear phosphoprotein 32 family, member A**	**-1,6**	0,001097
1450677_at	Chek1	**checkpoint kinase 1 homolog (S. pombe)**	**-1,6**	0,009464
1456706_at	4833441D16Rik	**RIKEN cDNA 4833441D16 gene**	**-1,6**	0,000346
1444458_at			**-1,6**	0,002502
1426631_at	Pus7	**pseudouridylate synthase 7 homolog (S. cerevisiae)**	**-1,6**	0,001195
1444728_at			**-1,6**	0,008412
1451347_at	Ino80e	**INO80 complex subunit E**	**-1,6**	0,000042
1447683_x_at	Mettl1	**methyltransferase like 1**	**-1,6**	0,000725
1430942_at	8430437O03Rik	**RIKEN cDNA 8430437O03 gene**	**-1,6**	0,000774
1417949_at	Ilf2	**interleukin enhancer binding factor 2**	**-1,6**	0,001094
1458292_at	Psma1	**proteasome (prosome, macropain) subunit, alpha type 1**	**-1,6**	0,001097
1431425_a_at	Rprd2	**regulation of nuclear pre-mRNA domain containing 2**	**-1,6**	0,000358
1452688_at	Prpf39	**PRP39 pre-mRNA processing factor 39 homolog (yeast)**	**-1,6**	0,006016
1441498_at			**-1,6**	0,002558
1425944_a_at	Rad51l3	**RAD51-like 3 (S. cerevisiae)**	**-1,6**	0,000028
1424603_at	Sumf1	**sulfatase modifying factor 1**	**-1,6**	0,013069
1460077_at	Ttc3	**tetratricopeptide repeat domain 3**	**-1,6**	0,002865
1428046_a_at	Zfx	**zinc finger protein X-linked**	**-1,6**	0,002966
1426053_a_at	Xpr1	**xenotropic and polytropic retrovirus receptor 1**	**-1,6**	0,000149
1431042_at	Paqr8	**progestin and adipoQ receptor family member VIII**	**-1,6**	0,007594
1435904_at	Eif2c3	**eukaryotic translation initiation factor 2C, 3**	**-1,6**	0,000064
1452055_at	Ctdsp1	**CTD (carboxy-terminal domain, RNA polymerase II, polypeptide A) small phosphatase 1**	**-1,6**	0,000014

1449046_a_at	Josd2	**Josephin domain containing 2**	**-1,6**	0,000006
1442178_at			**-1,6**	0,000687
1460486_at	Rabgap1	**RAB GTPase activating protein 1**	**-1,6**	0,000638
1444858_at			**-1,6**	0,000082
1457281_at	Dnajc21	**DnaJ (Hsp40) homolog, subfamily C, member 21**	**-1,6**	0,000036
1428924_at	Mocs3	**molybdenum cofactor synthesis 3**	**-1,6**	0,000010
1440894_at	Tmtc3	**transmembrane and tetratricopeptide repeat containing 3**	**-1,6**	0,000030
1458706_at			**-1,6**	0,001636
1436420_a_at	Ipo4	**importin 4**	**-1,6**	0,000992
1432910_at	Btbd7	**BTB (POZ) domain containing 7**	**-1,6**	0,000320
1443216_at			**-1,6**	0,001614
1430702_at	5830427D03Rik	**RIKEN cDNA 5830427D03 gene**	**-1,6**	0,000345
1456668_at			**-1,6**	0,000370
1434660_at	Alkbh1	**alkB, alkylation repair homolog 1 (E. coli)**	**-1,6**	0,009354
1417289_at	Plekha2	**pleckstrin homology domain-containing, family A (phosphoinositide binding specific) member 2**	**-1,6**	0,000003
1457480_at			**-1,6**	0,000711
1446736_at			**-1,6**	0,002671
1449582_at	Cdx1	**caudal type homeo box 1**	**-1,6**	0,000055
1438138_a_at	Pex6	**peroxisomal biogenesis factor 6**	**-1,6**	0,000029
1459884_at	Cox7c	**cytochrome c oxidase, subunit VIIc**	**-1,6**	0,006762
1434979_at	4933403F05Rik	**RIKEN cDNA 4933403F05 gene**	**-1,6**	0,000145
1437129_at	E330018D03Rik	**RIKEN cDNA E330018D03 gene**	**-1,6**	0,000635
1425114_at	Rbbp6	**retinoblastoma binding protein 6**	**-1,6**	0,002342
1429061_at	1810063B05Rik	**RIKEN cDNA 1810063B05 gene**	**-1,6**	0,000121
1455334_at	D330038O06Rik	**RIKEN cDNA D330038O06 gene**	**-1,6**	0,004763
1417848_at	Zfp704	**zinc finger protein 704**	**-1,6**	0,001610
1428720_s_at	2010309G21Rik	**RIKEN cDNA 2010309G21 gene**	**-1,6**	0,001547
1449608_a_at			**-1,6**	0,000524
1432472_a_at	Mccc2	**methylcrotonoyl-Coenzyme A carboxylase 2 (beta)**	**-1,6**	0,000141
1453304_s_at	Ly6e	**lymphocyte antigen 6 complex, locus E**	**-1,6**	0,000924
1448170_at	Siah2	**seven in absentia 2**	**-1,6**	0,000132
1442407_at			**-1,6**	0,000169
1456255_at	AI314180	**expressed sequence AI314180**	**-1,6**	0,008396
1456689_at	Rnf10	**ring finger protein 10**	**-1,6**	0,000344
1433931_at	C030046I01Rik	**RIKEN cDNA C030046I01 gene**	**-1,6**	0,000143
1439920_at			**-1,6**	0,000003
1417604_at	Camk1	**calcium/calmodulin-dependent protein kinase I**	**-1,6**	0,000003
1433769_at	Als2cl	**ALS2 C-terminal like**	**-1,6**	0,000604
1447099_at			**-1,6**	0,002973
1432352_at	Ccny	**cyclin Y**	**-1,6**	0,000935
1415972_at	Marcks	**myristoylated alanine rich protein kinase C substrate**	**-1,6**	0,000076

1419132_at	Tlr2	**toll-like receptor 2**	**-1,6**	0,002427
1451480_at	E2f4	**E2F transcription factor 4**	**-1,6**	0,000011
1430514_a_at	Cd99	**CD99 antigen**	**-1,6**	0,000026
1424033_at	Sfrs7	**splicing factor, arginine/serine-rich 7**	**-1,6**	0,000804
1423479_at	Nol11	**nucleolar protein 11**	**-1,6**	0,000682
1437916_at			**-1,6**	0,000810
1443773_at	Ylpm1	**YLP motif containing 1**	**-1,6**	0,000663
1441829_s_at	Akap10	**A kinase (PRKA) anchor protein 10**	**-1,6**	0,000009
1442235_at	Plagl2	**pleiomorphic adenoma gene-like 2**	**-1,6**	0,008748
1420682_at	Chrnb1	**cholinergic receptor, nicotinic, beta polypeptide 1 (muscle)**	**-1,6**	0,004715
1419191_at	Hipk3	**homeodomain interacting protein kinase 3**	**-1,6**	0,000053
1444224_at			**-1,6**	0,003078
1442445_at	2610027H17Rik	**RIKEN cDNA 2610027H17 gene**	**-1,6**	0,000919
1450678_at	Itgb2	**integrin beta 2**	**-1,6**	0,000404
1425964_x_at	Hspb1	**heat shock protein 1**	**-1,6**	0,000514
1455657_at	Smg1	**SMG1 homolog, phosphatidylinositol 3-kinase-related kinase (C. elegans)**	**-1,6**	0,000372
1437492_at	Mkx	**mohawk homeobox**	**-1,6**	0,000978
1441823_at	Zmiz1	**zinc finger, MIZ-type containing 1**	**-1,6**	0,001297
1422491_a_at	Bnip2	**BCL2/adenovirus E1B interacting protein 2**	**-1,6**	0,002041
1436509_at	Mlec	**malectin**	**-1,6**	0,000017
1416190_a_at	Sec61a1	**Sec61 alpha 1 subunit (S. cerevisiae)**	**-1,6**	0,000001
1438879_at			**-1,6**	0,001736
1459009_at			**-1,6**	0,001212
1417213_a_at	Rbm6	**RNA binding motif protein 6**	**-1,6**	0,000001
1430774_at	A430106A12Rik	**RIKEN cDNA A430106A12 gene**	**-1,6**	0,000049
1453524_at	Kif5b	**kinesin family member 5B**	**-1,6**	0,001314
1439139_at	D2Ertd640e	**DNA segment, Chr 2, ERATO Doi 640, expressed**	**-1,6**	0,000107
1418526_at	Fusip1	**FUS interacting protein (serine-arginine rich) 1**	**-1,6**	0,000291
1428949_at	Xpot	**exportin, tRNA (nuclear export receptor for tRNAs)**	**-1,6**	0,000021
1418860_a_at	Letmd1	**LETM1 domain containing 1**	**-1,6**	0,000007
1428698_at	Dpp8	**dipeptidylpeptidase 8**	**-1,6**	0,000001
1452463_x_at	Igk	**immunoglobulin kappa chain complex**	**-1,6**	0,008891
1417971_at	Nrm	**nurim (nuclear envelope membrane protein)**	**-1,6**	0,000070
1440287_at			**-1,6**	0,000211
1417500_a_at	Tgm2	**transglutaminase 2, C polypeptide**	**-1,6**	0,001019
1441547_at			**-1,6**	0,008346
1435426_s_at	Pisd	**phosphatidylserine decarboxylase**	**-1,6**	0,004280
1424776_a_at	Slc25a28	**solute carrier family 25, member 28**	**-1,6**	0,000045
1450916_at	Stau2	**staufen (RNA binding protein) homolog 2 (Drosophila)**	**-1,6**	0,000004
1435271_at	Irf3	**interferon regulatory factor 3**	**-1,6**	0,002310
1421895_at	Eif2s3x	**eukaryotic translation initiation factor 2, subunit 3, structural gene X-linked**	**-1,6**	0,000067

1452036_a_at	Tmpo	**thymopoietin**	**-1,6**	0,003940
1440671_at	A130012E19Rik	**RIKEN cDNA A130012E19 gene**	**-1,6**	0,001119
1421819_a_at	Set	**SET translocation**	**-1,6**	0,006512
1438674_a_at	Sfrs8	**splicing factor, arginine/serine-rich 8**	**-1,6**	0,000122
1452280_at	Farp1	**FERM, RhoGEF (Arhgef) and pleckstrin domain protein 1 (chondrocyte-derived)**	**-1,6**	0,000698
1445545_at	Tmem77	**transmembrane protein 77**	**-1,6**	0,000037
1442834_at			**-1,6**	0,002241
1455472_at	A630071D13Rik	**RIKEN cDNA A630071D13 gene**	**-1,6**	0,002588
1442911_at	Riok2	**RIO kinase 2 (yeast)**	**-1,6**	0,000389
1442338_at			**-1,6**	0,000364
1423966_at	Cd99l2	**CD99 antigen-like 2**	**-1,6**	0,000003
1451465_at	Ubl7	**ubiquitin-like 7 (bone marrow stromal cell-derived)**	**-1,6**	0,000012
1416019_at	Dr1	**down-regulator of transcription 1**	**-1,6**	0,000157
1451518_at	Zfp709	**zinc finger protein 709**	**-1,6**	0,000061
1446548_at			**-1,6**	0,000903
1435561_at	Erf	**Ets2 repressor factor**	**-1,6**	0,000001
1429897_a_at	D16Ertd472e	**DNA segment, Chr 16, ERATO Doi 472, expressed**	**-1,6**	0,003804
1426756_at	Galnt2	**UDP-N-acetyl-alpha-D-galactosamine:polypeptide N-acetylgalactosaminyltransferase 2**	**-1,6**	0,000010
1428506_at	Atic	**5-aminoimidazole-4-carboxamide ribonucleotide formyltransferase/IMP cyclohydrolase**	**-1,6**	0,000054
1459856_at			**-1,6**	0,003912
1434436_at	Morc4	**microrchidia 4**	**-1,6**	0,003481
1418230_a_at	Lims1	**LIM and senescent cell antigen-like domains 1**	**-1,6**	0,000188
1453852_at	Ddx50	**DEAD (Asp-Glu-Ala-Asp) box polypeptide 50**	**-1,6**	0,000630
1442971_at	Baz2b	**bromodomain adjacent to zinc finger domain, 2B**	**-1,6**	0,000586
1416389_a_at	Rcbtb2	**regulator of chromosome condensation (RCC1) and BTB (POZ) domain containing protein 2**	**-1,6**	0,000143
1418901_at	Cebpb	**CCAAT/enhancer binding protein (C/EBP), beta**	**-1,6**	0,008045
1429207_at	5730408K05Rik	**RIKEN cDNA 5730408K05 gene**	**-1,6**	0,003856
1431026_at	Nat12	**N-acetyltransferase 12**	**-1,6**	0,000034
1431196_at	Atp2c1	**ATPase, Ca++-sequestering**	**-1,6**	0,000022
1421260_a_at	Srm	**spermidine synthase**	**-1,6**	0,004647
1431287_at	Pcm1	**pericentriolar material 1**	**-1,6**	0,005889
1460035_at	Phb2	**prohibitin 2**	**-1,6**	0,000018
1450076_at	4933411K20Rik	**RIKEN cDNA 4933411K20 gene**	**-1,6**	0,000185
1452187_at	Rbm5	**RNA binding motif protein 5**	**-1,6**	0,000363
1452433_at			**-1,6**	0,000121
1443880_at	Zbtb39	**zinc finger and BTB domain containing 39**	**-1,6**	0,000003
1416536_at	Mum1	**melanoma associated antigen (mutated) 1**	**-1,6**	0,000021
1430135_at	Dnase2a	**deoxyribonuclease II alpha**	**-1,6**	0,004152
1428129_at	Lman1	**lectin, mannose-binding, 1**	**-1,6**	0,000003
1452024_a_at	Ldb1	**LIM domain binding 1**	**-1,6**	0,000059
1448103_s_at	Nono	**non-POU-domain-containing, octamer binding protein**	**-1,6**	0,001794

1437449_at	Rsad1	**radical S-adenosyl methionine domain containing 1**	**-1,6**	0,000210
1457673_at	6820431F20Rik	**RIKEN cDNA 6820431F20 gene**	**-1,6**	0,002976
1438887_a_at	Gmcl1	**germ cell-less homolog 1 (Drosophila)**	**-1,6**	0,000146
1424290_at	Osgin2	**oxidative stress induced growth inhibitor family member 2**	**-1,6**	0,000019
1449037_at	Crem	**cAMP responsive element modulator**	**-1,6**	0,000214
1453247_at	Zfp618	**zinc fingerprotein 618**	**-1,6**	0,005570
1448013_at	Usp24	**ubiquitin specific peptidase 24**	**-1,6**	0,000150
1439302_at	Uba6	**ubiquitin-like modifier activating enzyme 6**	**-1,6**	0,000322
1438072_at			**-1,6**	0,000934
1455336_at	Thap2	**THAP domain containing, apoptosis associated protein 2**	**-1,6**	0,001068
1448307_at	Psmg1	**proteasome (prosome, macropain) assembly chaperone 1**	**-1,6**	0,000476
1440847_at	Mtss1	**metastasis suppressor 1**	**-1,6**	0,001205
1448230_at	Usp10	**ubiquitin specific peptidase 10**	**-1,6**	0,000478
1457802_at	B930012P20Rik	**RIKEN cDNA B930012P20 gene**	**-1,6**	0,000492
1449044_at	Eef1e1	**eukaryotic translation elongation factor 1 epsilon 1**	**-1,6**	0,005174
1427040_at	Mdfic	**MyoD family inhibitor domain containing**	**-1,6**	0,003302
1448274_at	C1qbp	**complement component 1, q subcomponent binding protein**	**-1,6**	0,000608
1422718_at	Ap3s2	**adaptor-related protein complex 3, sigma 2 subunit**	**-1,6**	0,000007
1437067_at	Phtf2	**putative homeodomain transcription factor 2**	**-1,6**	0,007632
1455496_at	Pfas	**phosphoribosylformylglycinamidine synthase (FGAR amidotransferase)**	**-1,6**	0,000077
1448667_x_at	Tob2	**transducer of ERBB2, 2**	**-1,6**	0,000190
1436422_at	BC026590	**cDNA sequence BC026590**	**-1,6**	0,000047
1444856_at			**-1,6**	0,002741
1452426_x_at			**-1,6**	0,007162
1429110_a_at	Nsun4	**NOL1/NOP2/Sun domain family, member 4**	**-1,6**	0,000818
1442023_at	A530030E21Rik	**RIKEN cDNA A530030E21 gene**	**-1,6**	0,013231
1445267_at			**-1,6**	0,000258
1451154_a_at	Cugbp2	**CUG triplet repeat, RNA binding protein 2**	**-1,6**	0,007877
1452916_at	Wbp7	**WW domain binding protein 7**	**-1,6**	0,000014
1449861_at	Nek4	**NIMA (never in mitosis gene a)-related expressed kinase 4**	**-1,6**	0,000002
1435037_at	Perld1	**per1-like domain containing 1**	**-1,6**	0,001369
1456648_at			**-1,6**	0,007836
1422145_at	Mgat3	**mannoside acetylglucosaminyltransferase 3**	**-1,6**	0,007613
1452799_at	Fggy	**FGGY carbohydrate kinase domain containing**	**-1,6**	0,000360
1421337_at	SMUSG000000535	**predicted gene, ENSMUSG00000053512**	**-1,6**	0,000013
1417948_s_at	Ilf2	**interleukin enhancer binding factor 2**	**-1,6**	0,003070
1444744_at			**-1,6**	0,000034
1428187_at	Cd47	**CD47 antigen (Rh-related antigen, integrin-associated signal transducer)**	**-1,7**	0,000338
1442078_at			**-1,7**	0,000050
1425940_a_at	Ssbp3	**single-stranded DNA binding protein 3**	**-1,7**	0,000165
1415900_a_at	Kit	**kit oncogene**	**-1,7**	0,000522

1439276_at	Adar	**adenosine deaminase, RNA-specific**	**-1,7**	0,001449
1423334_at	Ergic1	**endoplasmic reticulum-golgi intermediate compartment (ERGIC) 1**	**-1,7**	0,000022
1416158_at	Nr2f2	**nuclear receptor subfamily 2, group F, member 2**	**-1,7**	0,000004
1415761_at	Mrpl52	**mitochondrial ribosomal protein L52**	**-1,7**	0,000361
1436177_at	Plekha2	**pleckstrin homology domain-containing, family A (phosphoinositide binding specific) member 2**	**-1,7**	0,000003
1452402_at			**-1,7**	0,001166
1426888_at	Ehmt2	**euchromatic histone lysine N-methyltransferase 2**	**-1,7**	0,000284
1428466_at	Chd3	**chromodomain helicase DNA binding protein 3**	**-1,7**	0,000126
1442126_at			**-1,7**	0,000568
1424055_at	Ncoa5	**nuclear receptor coactivator 5**	**-1,7**	0,000003
1453736_s_at	B230219D22Rik	**RIKEN cDNA B230219D22 gene**	**-1,7**	0,000064
1446703_at			**-1,7**	0,000403
1440268_at	Trim41	**tripartite motif-containing 41**	**-1,7**	0,000079
1415956_a_at	Pctk1	**PCTAIRE-motif protein kinase 1**	**-1,7**	0,000001
1418274_at	Nutf2	**nuclear transport factor 2**	**-1,7**	0,001755
1435328_at	Cyhr1	**cysteine and histidine rich 1**	**-1,7**	0,000001
1422954_at	Zfp60	**zinc finger protein 60**	**-1,7**	0,000873
1422167_at	Sema5a	**sema domain, seven thrombospondin repeats (type 1 and type 1-like), transmembrane domain (TM) and sl**	**-1,7**	0,000024
1455095_at	Hist2h2be	**histone cluster 2, H2be**	**-1,7**	0,000017
1443546_at			**-1,7**	0,002365
1449939_s_at	Dlk1	**delta-like 1 homolog (Drosophila)**	**-1,7**	0,010905
1422492_at	Cpox	**coproporphyrinogen oxidase**	**-1,7**	0,000038
1447156_at			**-1,7**	0,002139
1442580_at	A530020G20Rik	**RIKEN cDNA A530020G20 gene**	**-1,7**	0,000550
1434054_at	Sirt7	**sirtuin 7 (silent mating type information regulation 2, homolog) 7 (S. cerevisiae)**	**-1,7**	0,015511
1432901_at	Etl4	**enhancer trap locus 4**	**-1,7**	0,000591
1435319_at	Ip6k2	**inositol hexaphosphate kinase 2**	**-1,7**	0,000004
1459187_at			**-1,7**	0,003124
1441404_at			**-1,7**	0,000697
1457510_at			**-1,7**	0,000739
1415773_at	Ncl	**nucleolin**	**-1,7**	0,004947
1441415_at			**-1,7**	0,000301
1418128_at	Adcy6	**adenylate cyclase 6**	**-1,7**	0,000062
1426850_a_at	Map2k6	**mitogen-activated protein kinase kinase 6**	**-1,7**	0,000446
1443551_at	Atp2a2	**ATPase, Ca++ transporting, cardiac muscle, slow twitch 2**	**-1,7**	0,004350
1438326_at	Trmt6	**tRNA methyltransferase 6 homolog (S. cerevisiae)**	**-1,7**	0,003491
1450400_at	Tgs1	**trimethylguanosine synthase homolog (S. cerevisiae)**	**-1,7**	0,000408
1426988_at	Klhdc5	**kelch domain containing 5**	**-1,7**	0,000307
1419295_at	Creb3l1	**cAMP responsive element binding protein 3-like 1**	**-1,7**	0,004428
1439209_at	Tcf12	**transcription factor 12**	**-1,7**	0,000963
1423522_at	Npm3	**nucleoplasmin 3**	**-1,7**	0,000952

1428301_at	SMUSG000000687	**predicted gene, ENSMUSG00000068790**	**-1,7**	0,001189
1425087_at	2310003F16Rik	**RIKEN cDNA 2310003F16 gene**	**-1,7**	0,001249
1452764_at	Socs6	**suppressor of cytokine signaling 6**	**-1,7**	0,001346
1434067_at	AI662270	**expressed sequence AI662270**	**-1,7**	0,002259
1418915_at	Mmachc	**methylmalonic aciduria cblC type, with homocystinuria**	**-1,7**	0,000066
1424622_at	Hsf1	**heat shock factor 1**	**-1,7**	0,000009
1440892_at			**-1,7**	0,012066
1445966_at			**-1,7**	0,000375
1421955_a_at	Nedd4	**neural precursor cell expressed, developmentally down-regulated 4**	**-1,7**	0,000079
1444001_at			**-1,7**	0,000862
1425373_a_at	Psmg2	**proteasome (prosome, macropain) assembly chaperone 2**	**-1,7**	0,000655
1420894_at	Tgfbr1	**transforming growth factor, beta receptor I**	**-1,7**	0,000027
1441274_at			**-1,7**	0,000484
1422680_at	Ctr9	**Ctr9, Paf1/RNA polymerase II complex component, homolog (S. cerevisiae)**	**-1,7**	0,000013
1438941_x_at	Ampd2	**adenosine monophosphate deaminase 2 (isoform L)**	**-1,7**	0,002520
1425227_a_at	Atp6v0a1	**ATPase, H+ transporting, lysosomal V0 subunit A1**	**-1,7**	0,000210
1420887_a_at	Bcl2l1	**BCL2-like 1**	**-1,7**	0,000349
1415682_at	Xpo7	**exportin 7**	**-1,7**	0,000007
1435136_at	Whsc1	**Wolf-Hirschhorn syndrome candidate 1 (human)**	**-1,7**	0,009310
1423084_at	B3galt2	**UDP-Gal:betaGlcNAc beta 1,3-galactosyltransferase, polypeptide 2**	**-1,7**	0,003250
1442216_at			**-1,7**	0,000038
1429961_at	1700021C14Rik	**RIKEN cDNA 1700021C14 gene**	**-1,7**	0,001116
1437965_at	Heatr1	**HEAT repeat containing 1**	**-1,7**	0,000474
1429344_at	9,13E+15	**hypothetical 9130022E09**	**-1,7**	0,003841
1457479_at			**-1,7**	0,003516
1437756_at	Gimap9	**GTPase, IMAP family member 9**	**-1,7**	0,000628
1423726_at	Vat1	**vesicle amine transport protein 1 homolog (T californica)**	**-1,7**	0,000017
1427983_at	Zfp280c	**zinc finger protein 280C**	**-1,7**	0,008125
1424423_at	Lenep	**lens epithelial protein**	**-1,7**	0,000010
1431960_at	Wwox	**WW domain-containing oxidoreductase**	**-1,7**	0,000012
1443365_at	Htr4	**5 hydroxytryptamine (serotonin) receptor 4**	**-1,7**	0,000076
1415872_at	Hnrnph1	**heterogeneous nuclear ribonucleoprotein H1**	**-1,7**	0,008223
1428414_at	Ccny	**cyclin Y**	**-1,7**	0,010028
1417619_at	Gadd45gip1	**growth arrest and DNA-damage-inducible, gamma interacting protein 1**	**-1,7**	0,000158
1451026_at	Ftsj3	**FtsJ homolog 3 (E. coli)**	**-1,7**	0,004494
1428354_at	Foxk2	**forkhead box K2**	**-1,7**	0,000019
1460555_at	Fam65b	**family with sequence similarity 65, member B**	**-1,7**	0,002909
1441960_x_at	5730494M16Rik	**RIKEN cDNA 5730494M16 gene**	**-1,7**	0,005162
1438076_at	Rpl30	**ribosomal protein L30**	**-1,7**	0,003279
1445963_at			**-1,7**	0,000007
1438683_at	Wasf2	**WAS protein family, member 2**	**-1,7**	0,000113

1448348_at	Caprin1	**cell cycle associated protein 1**	**-1,7**	0,000028
1459961_a_at	Stat3	**signal transducer and activator of transcription 3**	**-1,7**	0,000047
1442006_at			**-1,7**	0,000001
1418980_a_at	Cnp	**2',3'-cyclic nucleotide 3' phosphodiesterase**	**-1,7**	0,000002
1419477_at	Clec2d	**C-type lectin domain family 2, member d**	**-1,7**	0,005793
1451849_a_at	Lmnb2	**lamin B2**	**-1,7**	0,000493
1415957_a_at	Rrp1	**ribosomal RNA processing 1 homolog (S. cerevisiae)**	**-1,7**	0,000001
1427884_at	Col3a1	**collagen, type III, alpha 1**	**-1,7**	0,015212
1437359_at	Rnps1	**ribonucleic acid binding protein S1**	**-1,7**	0,002052
1437789_at	Birc6	**baculoviral IAP repeat-containing 6**	**-1,7**	0,001125
1452508_x_at	Ptms	**parathymosin**	**-1,7**	0,000115
1423048_a_at	Tollip	**toll interacting protein**	**-1,7**	0,001738
1418527_a_at	Fusip1	**FUS interacting protein (serine-arginine rich) 1**	**-1,7**	0,001000
1428158_at	Akt1s1	**AKT1 substrate 1 (proline-rich)**	**-1,7**	0,000002
1459143_at	Chchd3	**coiled-coil-helix-coiled-coil-helix domain containing 3**	**-1,7**	0,000021
1439933_at	B430316J06Rik	**RIKEN cDNA B430316J06 gene**	**-1,7**	0,003674
1450246_at	Fut2	**fucosyltransferase 2**	**-1,7**	0,004580
1459196_at			**-1,7**	0,001851
1445919_at	AA409261	**expressed sequence AA409261**	**-1,7**	0,000207
1439148_a_at	Pfkl	**phosphofructokinase, liver, B-type**	**-1,7**	0,001722
1455092_at	LOC100043601	**hypothetical protein LOC100043601**	**-1,7**	0,002534
1430971_a_at	Aqr	**aquarius**	**-1,7**	0,000005
1430089_at	5830469G19Rik	**RIKEN cDNA 5830469G19 gene**	**-1,7**	0,000043
1416014_at	Abce1	**ATP-binding cassette, sub-family E (OABP), member 1**	**-1,7**	0,002350
1438800_at	Nagk	**N-acetylglucosamine kinase**	**-1,7**	0,000159
1448123_s_at	Tgfbi	**transforming growth factor, beta induced**	**-1,7**	0,002235
1426897_at	Rcc2	**regulator of chromosome condensation 2**	**-1,7**	0,013166
1418851_at	Trim39	**tripartite motif-containing 39**	**-1,7**	0,000439
1440736_at	AI131651	**expressed sequence AI131651**	**-1,7**	0,000018
1454823_at	Wdr37	**WD repeat domain 37**	**-1,7**	0,000004
1444760_at	Ncor1	**nuclear receptor co-repressor 1**	**-1,7**	0,001322
1438353_at			**-1,7**	0,000356
1441141_at	Amn1	**antagonist of mitotic exit network 1 homolog (S. cerevisiae)**	**-1,7**	0,001109
1439093_at			**-1,7**	0,001304
1424333_at	Rg9mtd1	**RNA (guanine-9-) methyltransferase domain containing 1**	**-1,7**	0,000903
1424874_a_at	Ptbp1	**polypyrimidine tract binding protein 1**	**-1,7**	0,000003
1458460_at			**-1,7**	0,000516
1439126_at	1110007A13Rik	**RIKEN cDNA 1110007A13 gene**	**-1,7**	0,001467
1430152_at	Eps15	**epidermal growth factor receptor pathway substrate 15**	**-1,7**	0,000344
1417420_at	Ccnd1	**cyclin D1**	**-1,7**	0,001049
1447166_at			**-1,7**	0,007471

1447491_at			**-1,7**	0,000108
1458056_at	Sfrs12	**splicing factor, arginine/serine-rich 12**	**-1,7**	0,006472
1450082_s_at	Etv5	**ets variant gene 5**	**-1,7**	0,000807
1451687_a_at	Hnf1b	**HNF1 homeobox B**	**-1,7**	0,000007
1452247_at	Fxr1	**fragile X mental retardation gene 1, autosomal homolog**	**-1,7**	0,001184
1452974_at	Nol8	**nucleolar protein 8**	**-1,7**	0,006437
1423909_at	Tmem176a	**transmembrane protein 176A**	**-1,7**	0,001463
1435018_at	5930434B04Rik	**RIKEN cDNA 5930434B04 gene**	**-1,7**	0,000001
1446982_at			**-1,7**	0,000029
1418296_at	Fxyd5	**FXYD domain-containing ion transport regulator 5**	**-1,7**	0,000210
1453556_x_at	Cd99	**CD99 antigen**	**-1,7**	0,000026
1433972_at	Camta1	**calmodulin binding transcription activator 1**	**-1,7**	0,002059
1441477_at	Calu	**calumenin**	**-1,7**	0,000018
1433621_at	Wdr41	**WD repeat domain 41**	**-1,7**	0,000005
1457588_at	C76213	**expressed sequence C76213**	**-1,7**	0,001053
1441624_at	Sorbs2	**sorbin and SH3 domain containing 2**	**-1,7**	0,010746
1449155_at	Polr3g	**polymerase (RNA) III (DNA directed) polypeptide G**	**-1,7**	0,000203
1416355_at	Rbmx	**RNA binding motif protein, X chromosome**	**-1,7**	0,000245
1460642_at	Traf4	**TNF receptor associated factor 4**	**-1,7**	0,000018
1459897_a_at	Sbsn	**suprabasin**	**-1,7**	0,000142
1439159_at	Rere	**arginine glutamic acid dipeptide (RE) repeats**	**-1,7**	0,007589
1426873_s_at	Jup	**junction plakoglobin**	**-1,7**	0,000423
1432360_a_at	Tmtc4	**transmembrane and tetratricopeptide repeat containing 4**	**-1,7**	0,001587
1419964_s_at	Hdgf	**hepatoma-derived growth factor**	**-1,7**	0,000111
1446345_at			**-1,7**	0,000394
1438065_at	Rprd1a	**regulation of nuclear pre-mRNA domain containing 1A**	**-1,7**	0,001320
1417988_at	Resp18	**regulated endocrine-specific protein 18**	**-1,7**	0,000506
1425544_at	Plekha5	**pleckstrin homology domain containing, family A member 5**	**-1,7**	0,000576
1426051_a_at	Cenpb	**centromere protein B**	**-1,7**	0,000004
1446172_at			**-1,7**	0,001986
1435036_at	Aspg	**asparaginase homolog (S. cerevisiae)**	**-1,7**	0,003065
1441487_at	Trim2	**tripartite motif-containing 2**	**-1,7**	0,000155
1427439_s_at	Prmt5	**protein arginine N-methyltransferase 5**	**-1,7**	0,000072
1428636_at	Steap2	**six transmembrane epithelial antigen of prostate 2**	**-1,7**	0,000409
1419460_at	Rpp14	**ribonuclease P 14 subunit (human)**	**-1,7**	0,002587
1450124_a_at	Atp2a3	**ATPase, Ca++ transporting, ubiquitous**	**-1,7**	0,000133
1418280_at	Klf6	**Kruppel-like factor 6**	**-1,7**	0,004401
1450873_at	Gtpbp4	**GTP binding protein 4**	**-1,7**	0,006135
1423271_at	Gjb2	**gap junction protein, beta 2**	**-1,7**	0,001044
1426085_a_at	Pxn	**paxillin**	**-1,7**	0,000200
1429096_at	2810455D13Rik	**RIKEN cDNA 2810455D13 gene**	**-1,7**	0,000106

1446090_at			-1,7	0,000273
1422735_at	Foxq1	forkhead box Q1	-1,7	0,007640
1438257_at			-1,7	0,000012
1429900_at	5330406M23Rik	RIKEN cDNA 5330406M23 gene	-1,7	0,001830
1426550_at	Sidt1	SID1 transmembrane family, member 1	-1,7	0,000009
1426958_at	Rps9	ribosomal protein S9	-1,7	0,012268
1442139_at			-1,7	0,001937
1453077_a_at	Snapc3	small nuclear RNA activating complex, polypeptide 3	-1,7	0,007419
1441117_at			-1,7	0,002135
1423333_at	Ergic1	endoplasmic reticulum-golgi intermediate compartment (ERGIC) 1	-1,7	0,000001
1446475_at			-1,7	0,000295
1456661_at			-1,7	0,000075
1429058_at	Tmem107	transmembrane protein 107	-1,7	0,000004
1427193_at	Brd8	bromodomain containing 8	-1,7	0,000346
1424620_at	Nop16	NOP16 nucleolar protein homolog (yeast)	-1,7	0,000467
1460633_at	Prpf19	PRP19/PSO4 pre-mRNA processing factor 19 homolog (S. cerevisiae)	-1,7	0,000073
1419647_a_at	Ier3	immediate early response 3	-1,7	0,006508
1430417_s_at	Neurl4	neuralized homolog 4 (Drosophila)	-1,7	0,000018
1417852_x_at	Clca1	chloride channel calcium activated 1	-1,7	0,000018
1436145_at			-1,7	0,000264
1460351_at	S100a11	S100 calcium binding protein A11 (calgizzarin)	-1,7	0,006176
1430780_a_at	Pmm1	phosphomannomutase 1	-1,7	0,001289
1421839_at	Abca1	ATP-binding cassette, sub-family A (ABC1), member 1	-1,7	0,007149
1438915_at	6720401G13Rik	RIKEN cDNA 6720401G13 gene	-1,7	0,003665
1440295_at	1110057K04Rik	RIKEN cDNA 1110057K04 gene	-1,7	0,000103
1439871_at			-1,7	0,000572
1428233_at	Cpsf6	cleavage and polyadenylation specific factor 6	-1,7	0,000193
1440543_at	Heatr5a	HEAT repeat containing 5A	-1,7	0,000063
1458393_at	Srr	serine racemase	-1,7	0,000043
1428404_at	2410025L10Rik	RIKEN cDNA 2410025L10 gene	-1,7	0,000006
1429545_at	Ube2i	ubiquitin-conjugating enzyme E2I	-1,7	0,001341
1456296_at	5830418K08Rik	RIKEN cDNA 5830418K08 gene	-1,7	0,005295
1430038_at	Gphn	gephyrin	-1,7	0,000065
1452498_at			-1,7	0,000130
1456040_at	Sf3b2	splicing factor 3b, subunit 2	-1,7	0,000000
1435134_at	Aadacl1	arylacetamide deacetylase-like 1	-1,7	0,015496
1455945_at	Zfp58	zinc finger protein 58	-1,7	0,000326
1449011_at	Slc12a7	solute carrier family 12, member 7	-1,7	0,000043
1457806_at	B830028B13Rik	RIKEN cDNA B830028B13 gene	-1,7	0,002936
1416915_at	Msh6	mutS homolog 6 (E. coli)	-1,7	0,000625
1431430_s_at	Trim59	tripartite motif-containing 59	-1,7	0,000525

1451109_a_at	Nedd4	**neural precursor cell expressed, developmentally down-regulated 4**	**-1,7**	0,000149
1449653_at			**-1,7**	0,000039
1422743_at	Phka1	**phosphorylase kinase alpha 1**	**-1,7**	0,000134
1448815_at	Ogg1	**8-oxoguanine DNA-glycosylase 1**	**-1,7**	0,000093
1437219_at			**-1,7**	0,000443
1432978_at	9030607L02Rik	**RIKEN cDNA 9030607L02 gene**	**-1,7**	0,000052
1426297_at	Tcfe2a	**transcription factor E2a**	**-1,7**	0,007518
1445933_at			**-1,7**	0,000078
1440717_at	AA407881	**expressed sequence AA407881**	**-1,7**	0,000016
1417927_at	Ddx19a	**DEAD (Asp-Glu-Ala-Asp) box polypeptide 19a**	**-1,7**	0,000001
1450927_at	Lztr1	**leucine-zipper-like transcriptional regulator, 1**	**-1,7**	0,000064
1448592_at	Crtap	**cartilage associated protein**	**-1,7**	0,000021
1456952_at			**-1,7**	0,000321
1440954_at			**-1,7**	0,000204
1435550_at	C430014K11Rik	**RIKEN cDNA C430014K11 gene**	**-1,7**	0,000001
1454699_at	Sesn1	**sestrin 1**	**-1,7**	0,000530
1441195_at	6030487A22Rik	**RIKEN cDNA 6030487A22 gene**	**-1,7**	0,000672
1440905_at	Hs2st1	**heparan sulfate 2-O-sulfotransferase 1**	**-1,7**	0,002719
1430045_at	Tsnax	**translin-associated factor X**	**-1,7**	0,000373
1458079_at	Usp40	**ubiquitin specific peptidase 40**	**-1,7**	0,000039
1446071_at	Steap2	**six transmembrane epithelial antigen of prostate 2**	**-1,7**	0,001433
1431102_at	Cep350	**centrosomal protein 350**	**-1,7**	0,000047
1427079_at	Mapre3	**microtubule-associated protein, RP/EB family, member 3**	**-1,7**	0,002856
1427680_a_at	Nfib	**nuclear factor I/B**	**-1,7**	0,001155
1435078_at	Tanc2	**tetratricopeptide repeat, ankyrin repeat and coiled-coil containing 2**	**-1,7**	0,005879
1441718_at			**-1,7**	0,001455
1451249_at	Trmt1	**TRM1 tRNA methyltransferase 1 homolog (S. cerevisiae)**	**-1,7**	0,000013
1443114_at	Zmym4	**zinc finger, MYM-type 4**	**-1,7**	0,000923
1446472_at	Ahctf1	**AT hook containing transcription factor 1**	**-1,7**	0,000070
1418181_at	Ptp4a3	**protein tyrosine phosphatase 4a3**	**-1,7**	0,000980
1455627_at	Col8a1	**collagen, type VIII, alpha 1**	**-1,7**	0,009892
1456377_x_at	Limd2	**LIM domain containing 2**	**-1,7**	0,014421
1440637_at			**-1,7**	0,000150
1446164_at	Tgs1	**trimethylguanosine synthase homolog (S. cerevisiae)**	**-1,7**	0,001164
1418374_at	Fxyd3	**FXYD domain-containing ion transport regulator 3**	**-1,7**	0,001537
1438165_x_at	Vat1	**vesicle amine transport protein 1 homolog (T californica)**	**-1,7**	0,001023
1448277_at	Pold2	**polymerase (DNA directed), delta 2, regulatory subunit**	**-1,7**	0,000034
1435244_at	Vav2	**vav 2 oncogene**	**-1,7**	0,012505
1459537_at			**-1,7**	0,001081
1455606_at	N4bp1	**NEDD4 binding protein 1**	**-1,7**	0,000022
1421298_a_at	Hipk1	**homeodomain interacting protein kinase 1**	**-1,7**	0,000677

1436034_at	Cep68	**centrosomal protein 68**	**-1,7**	0,002122
1452175_at	1810026J23Rik	**RIKEN cDNA 1810026J23 gene**	**-1,7**	0,000007
1421271_at	Sh3rf1	**SH3 domain containing ring finger 1**	**-1,7**	0,000411
1459973_x_at	Dpp4	**dipeptidylpeptidase 4**	**-1,7**	0,000383
1424222_s_at	Rad23b	**RAD23b homolog (S. cerevisiae)**	**-1,7**	0,000001
1416283_at	Gart	**phosphoribosylglycinamide formyltransferase**	**-1,7**	0,001354
1451456_at	6430706D22Rik	**RIKEN cDNA 6430706D22 gene**	**-1,7**	0,000274
1419186_a_at	St8sia4	**ST8 alpha-N-acetyl-neuraminide alpha-2,8-sialyltransferase 4**	**-1,7**	0,007986
1419270_a_at	Dut	**deoxyuridine triphosphatase**	**-1,7**	0,007152
1434707_at	Sbf1	**SET binding factor 1**	**-1,7**	0,000001
1423493_a_at	Nfix	**nuclear factor I/X**	**-1,7**	0,000001
1438816_at	Ahctf1	**AT hook containing transcription factor 1**	**-1,7**	0,000768
1443526_at			**-1,7**	0,000935
1418991_at	Bak1	**BCL2-antagonist/killer 1**	**-1,7**	0,000075
1421033_a_at	Tcerg1	**transcription elongation regulator 1 (CA150)**	**-1,7**	0,000539
1453212_at	Zfp383	**zinc finger protein 383**	**-1,7**	0,003130
1440028_at			**-1,7**	0,010176
1429739_a_at	Patz1	**POZ (BTB) and AT hook containing zinc finger 1**	**-1,7**	0,010323
1441185_at	Msi2	**Musashi homolog 2 (Drosophila)**	**-1,7**	0,000569
1427353_at	Clasp1	**CLIP associating protein 1**	**-1,7**	0,005378
1416846_a_at	Pdzrn3	**PDZ domain containing RING finger 3**	**-1,7**	0,000443
1460253_at	Cmtm7	**CKLF-like MARVEL transmembrane domain containing 7**	**-1,7**	0,000210
1428873_a_at	Msl1	**male-specific lethal 1 homolog (Drosophila)**	**-1,7**	0,000003
1436539_at	Clmn	**calmin**	**-1,7**	0,000145
1451574_at	Bcl9	**B-cell CLL/lymphoma 9**	**-1,7**	0,000006
1457392_at			**-1,7**	0,000160
1425329_a_at	Cyb5r3	**cytochrome b5 reductase 3**	**-1,7**	0,000016
1424082_at	Tbc1d13	**TBC1 domain family, member 13**	**-1,7**	0,000005
1459358_at			**-1,7**	0,000020
1421809_at	Dgcr2	**DiGeorge syndrome critical region gene 2**	**-1,7**	0,000052
1443238_at			**-1,7**	0,000170
1428383_a_at	2310021P13Rik	**RIKEN cDNA 2310021P13 gene**	**-1,7**	0,000000
1450012_x_at	Ywhag	**tyrosine 3-monooxygenase/tryptophan 5-monooxygenase activation protein, gamma polypeptide**	**-1,7**	0,000088
1459344_at	9630019E01Rik	**RIKEN cDNA 9630019E01 gene**	**-1,7**	0,001207
1444466_at	Ncald	**neurocalcin delta**	**-1,7**	0,000095
1439895_at	AU021025	**expressed sequence AU021025**	**-1,7**	0,000098
1451377_a_at	Aaas	**achalasia, adrenocortical insufficiency, alacrimia**	**-1,7**	0,000231
1420836_at	Slc25a30	**solute carrier family 25, member 30**	**-1,7**	0,000534
1438433_at	Whamm	**WAS protein homolog associated with actin, golgi membranes and microtubules**	**-1,7**	0,000022
1416237_at	Mpzl2	**myelin protein zero-like 2**	**-1,7**	0,000003
1421038_a_at	Kcnn4	**potassium intermediate/small conductance calcium-activated channel, subfamily N, member 4**	**-1,7**	0,000054

1419033_at	Papola	**poly (A) polymerase alpha**	**-1,7**	0,002305
1421574_at	Rap2a	**RAS related protein 2a**	**-1,7**	0,000094
1434209_at	Prmt6	**protein arginine N-methyltransferase 6**	**-1,7**	0,000003
1447024_at			**-1,7**	0,001172
1426403_at	Actr1b	**ARP1 actin-related protein 1 homolog B, centractin beta (yeast)**	**-1,7**	0,000001
1440622_at			**-1,7**	0,000179
1442052_at			**-1,7**	0,000733
1422799_at	Bat2	**HLA-B associated transcript 2**	**-1,7**	0,000010
1425206_a_at	Ube3a	**ubiquitin protein ligase E3A**	**-1,7**	0,007761
1433266_at	2810416A17Rik	**RIKEN cDNA 2810416A17 gene**	**-1,7**	0,014343
1425483_at	LOC100044677	**similar to thymus high mobility group box protein TOX**	**-1,7**	0,001173
1434360_s_at	Ptprg	**protein tyrosine phosphatase, receptor type, G**	**-1,7**	0,000097
1448656_at	Cacnb3	**calcium channel, voltage-dependent, beta 3 subunit**	**-1,7**	0,000014
1450537_at	Mid2	**midline 2**	**-1,7**	0,001323
1459670_at			**-1,7**	0,000571
1453193_s_at	Kif12	**kinesin family member 12**	**-1,7**	0,008514
1441642_at			**-1,7**	0,000272
1423847_at	Ncapd2	**non-SMC condensin I complex, subunit D2**	**-1,7**	0,001182
1440317_at	C130068B02Rik	**RIKEN cDNA C130068B02 gene**	**-1,7**	0,000933
1430075_at	Sf3b3	**splicing factor 3b, subunit 3**	**-1,7**	0,001748
1417786_a_at	Rgs19	**regulator of G-protein signaling 19**	**-1,7**	0,003598
1436518_at	Usp46	**ubiquitin specific peptidase 46**	**-1,7**	0,000069
1438862_at	A630005I04Rik	**RIKEN cDNA A630005I04 gene**	**-1,7**	0,000571
1416050_a_at	Scarb1	**scavenger receptor class B, member 1**	**-1,7**	0,000022
1448541_at	Klc1	**kinesin light chain 1**	**-1,7**	0,000057
1424866_at	Usp43	**ubiquitin specific peptidase 43**	**-1,7**	0,000000
1422088_at	Mycl1	**v-myc myelocytomatosis viral oncogene homolog 1, lung carcinoma derived (avian)**	**-1,7**	0,000254
1437797_at	Atp2a2	**ATPase, Ca++ transporting, cardiac muscle, slow twitch 2**	**-1,7**	0,000623
1415869_a_at	Trim28	**tripartite motif-containing 28**	**-1,7**	0,000077
1436320_at			**-1,7**	0,000000
1458159_at			**-1,7**	0,003186
1423543_at	Swap70	**SWA-70 protein**	**-1,7**	0,000094
1423952_a_at	Krt7	**keratin 7**	**-1,7**	0,002401
1449116_a_at	Dtymk	**deoxythymidylate kinase**	**-1,7**	0,010033
1448101_s_at	Trim27	**tripartite motif-containing 27**	**-1,7**	0,000000
1445598_at			**-1,7**	0,003769
1423520_at	Lmnb1	**lamin B1**	**-1,7**	0,000144
1426532_at	Zmynd11	**zinc finger, MYND domain containing 11**	**-1,7**	0,000009
1452629_at	Safb2	**scaffold attachment factor B2**	**-1,7**	0,000006
1430043_at	Ttc19	**tetratricopeptide repeat domain 19**	**-1,7**	0,000090
1433647_s_at	Rhobtb3	**Rho-related BTB domain containing 3**	**-1,7**	0,004853

1457466_at	AA409368	**expressed sequence AA409368**	**-1,7**	0,009349
1460164_at	Spin1	**spindlin 1**	**-1,7**	0,000006
1443991_at	Dock1	**dedicator of cytokinesis 1**	**-1,7**	0,000055
1428672_at	Snrpf	**small nuclear ribonucleoprotein polypeptide F**	**-1,7**	0,004049
1439127_at	AI314180	**expressed sequence AI314180**	**-1,7**	0,003002
1460237_at	Trim8	**tripartite motif protein 8**	**-1,7**	0,000002
1438001_x_at	Reep5	**receptor accessory protein 5**	**-1,7**	0,000000
1426296_at	Rad52	**RAD52 homolog (S. cerevisiae)**	**-1,7**	0,000040
1416853_at	Ncdn	**neurochondrin**	**-1,7**	0,000004
1445152_at			**-1,7**	0,001997
1457189_at			**-1,7**	0,003540
1436791_at	Wnt5a	**wingless-related MMTV integration site 5A**	**-1,7**	0,004496
1441020_at			**-1,7**	0,006760
1426513_at	Rbm28	**RNA binding motif protein 28**	**-1,7**	0,000349
1448138_at	Ppp2r4	**protein phosphatase 2A, regulatory subunit B (PR 53)**	**-1,7**	0,000001
1427831_s_at	Zfp260	**zinc finger protein 260**	**-1,7**	0,001617
1446926_at	Pycard	**PYD and CARD domain containing**	**-1,7**	0,000026
1455087_at	D7Ertd715e	**DNA segment, Chr 7, ERATO Doi 715, expressed**	**-1,7**	0,012384
1451987_at	Arrb2	**arrestin, beta 2**	**-1,7**	0,000023
1417542_at	Rps6ka2	**ribosomal protein S6 kinase, polypeptide 2**	**-1,7**	0,000226
1449110_at	Rhob	**ras homolog gene family, member B**	**-1,7**	0,000016
1452400_a_at	Hoxa11as	**HOXA11 antisense RNA (non-protein coding)**	**-1,7**	0,000540
1442824_at	8030497I03Rik	**RIKEN cDNA 8030497I03 gene**	**-1,7**	0,001292
1442959_at	Birc6	**baculoviral IAP repeat-containing 6**	**-1,7**	0,000926
1426385_x_at	Ywhae	**tyrosine 3-monooxygenase/tryptophan 5-monooxygenase activation protein, epsilon polypeptide**	**-1,7**	0,000000
1448788_at	Cd200	**CD200 antigen**	**-1,7**	0,000036
1416124_at	Ccnd2	**cyclin D2**	**-1,7**	0,001347
1425932_a_at	Cugbp1	**CUG triplet repeat, RNA binding protein 1**	**-1,7**	0,000005
1452772_at	Tnks2	**tankyrase, TRF1-interacting ankyrin-related ADP-ribose polymerase 2**	**-1,7**	0,000196
1429065_at	1200009F10Rik	**RIKEN cDNA 1200009F10 gene**	**-1,7**	0,000538
1424570_at	Ddx46	**DEAD (Asp-Glu-Ala-Asp) box polypeptide 46**	**-1,7**	0,000068
1440896_at			**-1,7**	0,001443
1452430_s_at	Sfrs1	**splicing factor, arginine/serine-rich 1 (ASF/SF2)**	**-1,7**	0,000047
1436796_at	Matr3	**matrin 3**	**-1,7**	0,000039
1431173_at	Fam53b	**family with sequence similarity 53, member B**	**-1,7**	0,001602
1454311_at			**-1,7**	0,002262
1424203_at	Ncln	**nicalin homolog (zebrafish)**	**-1,7**	0,000002
1458213_at			**-1,7**	0,000040
1442843_at	4933411D12Rik	**RIKEN cDNA 4933411D12 gene**	**-1,7**	0,000370
1433742_at	Kank1	**KN motif and ankyrin repeat domains 1**	**-1,7**	0,000023
1430134_a_at	Yars2	**tyrosyl-tRNA synthetase 2 (mitochondrial)**	**-1,7**	0,000295

1446510_at			**-1,7**	0,000197
1428543_at	Ppat	**phosphoribosyl pyrophosphate amidotransferase**	**-1,7**	0,008199
1442111_at	D430033H22Rik	**RIKEN cDNA D430033H22 gene**	**-1,7**	0,000009
1448126_at	Fam60a	**family with sequence similarity 60, member A**	**-1,7**	0,002786
1428854_at	Tmed8	**transmembrane emp24 domain containing 8**	**-1,7**	0,001523
1418032_at	Itfg2	**integrin alpha FG-GAP repeat containing 2**	**-1,7**	0,000020
1418397_at	Zfp275	**zinc finger protein 275**	**-1,7**	0,000184
1446504_at			**-1,7**	0,000063
1423488_at	Mmd	**monocyte to macrophage differentiation-associated**	**-1,7**	0,000643
1457173_at			**-1,8**	0,002016
1442464_at	Fbxl20	**F-box and leucine-rich repeat protein 20**	**-1,8**	0,000040
1448630_a_at	Sdhc	**succinate dehydrogenase complex, subunit C, integral membrane protein**	**-1,8**	0,000001
1458156_at	E230012J19Rik	**RIKEN cDNA E230012J19 gene**	**-1,8**	0,009952
1423750_a_at	Sf1	**splicing factor 1**	**-1,8**	0,000000
1460579_at	Dnpep	**aspartyl aminopeptidase**	**-1,8**	0,001726
1416774_at	Wee1	**WEE 1 homolog 1 (S. pombe)**	**-1,8**	0,000739
1425587_a_at	Ptprj	**protein tyrosine phosphatase, receptor type, J**	**-1,8**	0,000558
1457468_at			**-1,8**	0,000428
1434034_at	Cerk	**ceramide kinase**	**-1,8**	0,000005
1423832_at	Prkag2	**protein kinase, AMP-activated, gamma 2 non-catalytic subunit**	**-1,8**	0,000982
1416140_a_at	Dhx30	**DEAH (Asp-Glu-Ala-His) box polypeptide 30**	**-1,8**	0,000004
1424407_s_at	Cbx6	**chromobox homolog 6**	**-1,8**	0,010261
1424665_at	Gpatch8	**G patch domain containing 8**	**-1,8**	0,000116
1417062_at	Armc10	**armadillo repeat containing 10**	**-1,8**	0,001520
1430808_at	Tbc1d5	**TBC1 domain family, member 5**	**-1,8**	0,000006
1417686_at	Lgals12	**lectin, galactose binding, soluble 12**	**-1,8**	0,000105
1427049_s_at	Smo	**smoothened homolog (Drosophila)**	**-1,8**	0,002885
1428560_at	Xpo5	**exportin 5**	**-1,8**	0,000296
1455030_at	Ptprj	**protein tyrosine phosphatase, receptor type, J**	**-1,8**	0,000244
1434618_at	Crebzf	**CREB/ATF bZIP transcription factor**	**-1,8**	0,001334
1425273_s_at	Emp2	**epithelial membrane protein 2**	**-1,8**	0,000817
1434741_at	Rreb1	**ras responsive element binding protein 1**	**-1,8**	0,000022
1448124_at	Gusb	**glucuronidase, beta**	**-1,8**	0,007636
1422576_at	Atxn10	**ataxin 10**	**-1,8**	0,001026
1428708_x_at	Ptms	**parathymosin**	**-1,8**	0,000000
1420650_at	Zfhx3	**zinc finger homeobox 3**	**-1,8**	0,001803
1443721_x_at	Sbno2	**strawberry notch homolog 2 (Drosophila)**	**-1,8**	0,000067
1430485_at	Trpc2	**transient receptor potential cation channel, subfamily C, member 2**	**-1,8**	0,008250
1460016_at	Tmem164	**transmembrane protein 164**	**-1,8**	0,000029
1439553_s_at	Nutf2	**nuclear transport factor 2**	**-1,8**	0,001274
1442358_at	AA409587	**expressed sequence AA409587**	**-1,8**	0,001122

1459150_at			**-1,8**	0,002729
1430163_at	Rab43	**RAB43, member RAS oncogene family**	**-1,8**	0,000088
1435934_at	Ndufab1	**NADH dehydrogenase (ubiquinone) 1, alpha/beta subcomplex, 1**	**-1,8**	0,002395
1444520_at	Fryl	**furry homolog-like (Drosophila)**	**-1,8**	0,000002
1447109_at			**-1,8**	0,002409
1421972_s_at	Hcfc1	**host cell factor C1**	**-1,8**	0,000007
1439705_at			**-1,8**	0,000038
1447944_at	Zkscan1	**zinc finger with KRAB and SCAN domains 1**	**-1,8**	0,000271
1442603_at			**-1,8**	0,000696
1441445_at	Per3	**period homolog 3 (Drosophila)**	**-1,8**	0,001139
1435087_at	Zfp362	**zinc finger protein 362**	**-1,8**	0,000023
1454686_at	6430706D22Rik	**RIKEN cDNA 6430706D22 gene**	**-1,8**	0,000001
1448698_at	Ccnd1	**cyclin D1**	**-1,8**	0,000144
1448796_s_at	Tbrg4	**transforming growth factor beta regulated gene 4**	**-1,8**	0,000009
1426863_at	Rbmx	**RNA binding motif protein, X chromosome**	**-1,8**	0,007141
1444973_at			**-1,8**	0,001317
1458111_at	Fam20b	**family with sequence similarity 20, member B**	**-1,8**	0,000017
1441411_at	Lims1	**LIM and senescent cell antigen-like domains 1**	**-1,8**	0,000340
1450843_a_at	Serpinh1	**serine (or cysteine) peptidase inhibitor, clade H, member 1**	**-1,8**	0,000266
1441140_at			**-1,8**	0,001100
1458069_at			**-1,8**	0,000596
1438244_at	Nfib	**nuclear factor I/B**	**-1,8**	0,000130
1426956_a_at	Trp53bp1	**transformation related protein 53 binding protein 1**	**-1,8**	0,002049
1440965_at	Pigl	**phosphatidylinositol glycan anchor biosynthesis, class L**	**-1,8**	0,001863
1427251_at	Atp2a2	**ATPase, Ca++ transporting, cardiac muscle, slow twitch 2**	**-1,8**	0,000007
1423924_s_at	Tspan14	**tetraspanin 14**	**-1,8**	0,000052
1452251_at	Nbea	**neurobeachin**	**-1,8**	0,001815
1430700_a_at	Pla2g7	**phospholipase A2, group VII (platelet-activating factor acetylhydrolase, plasma)**	**-1,8**	0,012075
1426555_at	Scpep1	**serine carboxypeptidase 1**	**-1,8**	0,000002
1451364_at	Polr3gl	**polymerase (RNA) III (DNA directed) polypeptide G like**	**-1,8**	0,000002
1434448_at	Txlna	**taxilin alpha**	**-1,8**	0,000001
1447150_at	Mycbp2	**MYC binding protein 2**	**-1,8**	0,002113
1422769_at	Syncrip	**synaptotagmin binding, cytoplasmic RNA interacting protein**	**-1,8**	0,001346
1440513_at	C80258	**expressed sequence C80258**	**-1,8**	0,001075
1417176_at	Csnk1e	**casein kinase 1, epsilon**	**-1,8**	0,002135
1455854_a_at	Ssh1	**slingshot homolog 1 (Drosophila)**	**-1,8**	0,000101
1422064_a_at	Zbtb20	**zinc finger and BTB domain containing 20**	**-1,8**	0,000003
1452057_at	Actr1b	**ARP1 actin-related protein 1 homolog B, centractin beta (yeast)**	**-1,8**	0,000004
1438839_a_at	Ywhae	**tyrosine 3-monooxygenase/tryptophan 5-monooxygenase activation protein, epsilon polypeptide**	**-1,8**	0,000000
1417623_at	Slc12a2	**solute carrier family 12, member 2**	**-1,8**	0,009263
1421018_at	1110018J18Rik	**RIKEN cDNA 1110018J18 gene**	**-1,8**	0,000070

1438437_a_at	4933439C10Rik	**RIKEN cDNA 4933439C10 gene**	**-1,8**	0,001174
1446719_at	Atad2b	**ATPase family, AAA domain containing 2B**	**-1,8**	0,000050
1454120_a_at	Pcgf6	**polycomb group ring finger 6**	**-1,8**	0,009071
1458299_s_at	Nfkbie	**nuclear factor of kappa light polypeptide gene enhancer in B-cells inhibitor, epsilon**	**-1,8**	0,000033
1459003_at	Fhl1	**four and a half LIM domains 1**	**-1,8**	0,000324
1423141_at	Lipa	**lysosomal acid lipase A**	**-1,8**	0,006750
1442556_at			**-1,8**	0,000503
1416203_at	Aqp1	**aquaporin 1**	**-1,8**	0,001984
1426801_at	sep-08	**septin 8**	**-1,8**	0,000002
1453740_a_at	Ccnl2	**cyclin L2**	**-1,8**	0,001109
1416514_a_at	Fscn1	**fascin homolog 1, actin bundling protein (Strongylocentrotus purpuratus)**	**-1,8**	0,013679
1439090_at	Tbc1d23	**TBC1 domain family, member 23**	**-1,8**	0,000114
1448767_s_at	Gjb1	**gap junction protein, beta 1**	**-1,8**	0,000008
1457292_at	Taf1d	**TATA box binding protein (Tbp)-associated factor, RNA polymerase I, D**	**-1,8**	0,014550
1442470_at			**-1,8**	0,000096
1436366_at	Ppp1r15b	**protein phosphatase 1, regulatory (inhibitor) subunit 15b**	**-1,8**	0,000455
1417975_at	Kpna4	**karyopherin (importin) alpha 4**	**-1,8**	0,000001
1449271_a_at	Hebp2	**heme binding protein 2**	**-1,8**	0,015176
1442811_at	Rgmb	**RGM domain family, member B**	**-1,8**	0,001520
1440416_at			**-1,8**	0,000015
1445900_at			**-1,8**	0,000075
1440660_at			**-1,8**	0,003431
1417030_at	Tmem206	**transmembrane protein 206**	**-1,8**	0,004206
1418499_a_at	Kcne3	**potassium voltage-gated channel, Isk-related subfamily, gene 3**	**-1,8**	0,012274
1439041_at	Slc39a10	**solute carrier family 39 (zinc transporter), member 10**	**-1,8**	0,002271
1429719_at	Foxp4	**forkhead box P4**	**-1,8**	0,000000
1440038_at	B430007K19Rik	**RIKEN cDNA B430007K19 gene**	**-1,8**	0,002023
1420816_at	Ywhag	**tyrosine 3-monooxygenase/tryptophan 5-monooxygenase activation protein, gamma polypeptide**	**-1,8**	0,000000
1416818_at	Parva	**parvin, alpha**	**-1,8**	0,000126
1459315_at			**-1,8**	0,000082
1416661_at	Eif3a	**eukaryotic translation initiation factor 3, subunit A**	**-1,8**	0,000022
1458385_at	Hspa4l	**heat shock protein 4 like**	**-1,8**	0,000148
1436907_at	Nav1	**neuron navigator 1**	**-1,8**	0,004198
1424062_at	Ube2d1	**ubiquitin-conjugating enzyme E2D 1, UBC4/5 homolog (yeast)**	**-1,8**	0,000084
1439580_at	B930053N05Rik	**RIKEN cDNA B930053N05 gene**	**-1,8**	0,003301
1455218_at	6330503K22Rik	**RIKEN cDNA 6330503K22 gene**	**-1,8**	0,004536
1442107_at	Flnb	**filamin, beta**	**-1,8**	0,003276
1424772_at	H2afj	**H2A histone family, member J**	**-1,8**	0,000004
1456043_at	Usp22	**ubiquitin specific peptidase 22**	**-1,8**	0,000064
1458708_at			**-1,8**	0,000623
1449041_a_at	Trip6	**thyroid hormone receptor interactor 6**	**-1,8**	0,000495

1427152_at	Qser1	**glutamine and serine rich 1**	**-1,8**	0,000910
1448056_at	Huwe1	**HECT, UBA and WWE domain containing 1**	**-1,8**	0,000048
1456553_at			**-1,8**	0,002069
1460032_at			**-1,8**	0,000112
1455101_at	Phactr2	**phosphatase and actin regulator 2**	**-1,8**	0,000009
1460549_a_at	Cdc23	**CDC23 (cell division cycle 23, yeast, homolog)**	**-1,8**	0,000011
1458414_at	D2Ertd93e	**DNA segment, Chr 2, ERATO Doi 93, expressed**	**-1,8**	0,000070
1455655_a_at	Tardbp	**TAR DNA binding protein**	**-1,8**	0,002629
1424332_at	Rab40c	**Rab40c, member RAS oncogene family**	**-1,8**	0,001473
1457214_at			**-1,8**	0,000312
1435343_at	Dock10	**dedicator of cytokinesis 10**	**-1,8**	0,013942
1460403_at	Psip1	**PC4 and SFRS1 interacting protein 1**	**-1,8**	0,006355
1430039_at	Cdkal1	**CDK5 regulatory subunit associated protein 1-like 1**	**-1,8**	0,000014
1450983_at	Akap8	**A kinase (PRKA) anchor protein 8**	**-1,8**	0,000091
1428430_at	Rgmb	**RGM domain family, member B**	**-1,8**	0,000002
1443258_at	Foxp1	**forkhead box P1**	**-1,8**	0,000013
1452708_a_at	Luc7l	**Luc7 homolog (S. cerevisiae)-like**	**-1,8**	0,005856
1440465_at			**-1,8**	0,000039
1452482_at	Erbb3	**v-erb-b2 erythroblastic leukemia viral oncogene homolog 3 (avian)**	**-1,8**	0,003235
1419654_at	Tle3	**transducin-like enhancer of split 3, homolog of Drosophila E(spl)**	**-1,8**	0,000004
1426692_at	Ccdc97	**coiled-coil domain containing 97**	**-1,8**	0,000002
1432344_a_at	Aplp2	**amyloid beta (A4) precursor-like protein 2**	**-1,8**	0,000214
1454636_at	Cbx5	**chromobox homolog 5 (Drosophila HP1a)**	**-1,8**	0,000211
1426849_at	Sec24b	**Sec24 related gene family, member B (S. cerevisiae)**	**-1,8**	0,000356
1453319_at	Ccar1	**cell division cycle and apoptosis regulator 1**	**-1,8**	0,006277
1416792_at	Ppm1g	**protein phosphatase 1G (formerly 2C), magnesium-dependent, gamma isoform**	**-1,8**	0,000001
1425461_at	Fbxw11	**F-box and WD-40 domain protein 11**	**-1,8**	0,000003
1450781_at	Hmga2	**high mobility group AT-hook 2**	**-1,8**	0,000065
1440139_at			**-1,8**	0,003256
1447615_at			**-1,8**	0,000864
1443522_s_at	Phip	**pleckstrin homology domain interacting protein**	**-1,8**	0,001687
1460342_s_at	Mprip	**myosin phosphatase Rho interacting protein**	**-1,8**	0,000120
1445618_at			**-1,8**	0,003776
1448012_at	C76336	**expressed sequence C76336**	**-1,8**	0,000018
1427956_at	Pcgf1	**polycomb group ring finger 1**	**-1,8**	0,000006
1439802_at	Stk35	**serine/threonine kinase 35**	**-1,8**	0,000006
1433776_at	Lhfp	**lipoma HMGIC fusion partner**	**-1,8**	0,004187
1446892_at			**-1,8**	0,002123
1441894_s_at	Grasp	**GRP1 (general receptor for phosphoinositides 1)-associated scaffold protein**	**-1,8**	0,000073
1427844_a_at	Cebpb	**CCAAT/enhancer binding protein (C/EBP), beta**	**-1,8**	0,004515
1428732_at	1700008J07Rik	**RIKEN cDNA 1700008J07 gene**	**-1,8**	0,000003

1440586_at	B430203I24Rik	**RIKEN cDNA B430203I24 gene**	**-1,8**	0,000060
1455084_x_at	Shmt2	**serine hydroxymethyltransferase 2 (mitochondrial)**	**-1,8**	0,000303
1437775_at	Dlst	**dihydrolipoamide S-succinyltransferase (E2 component of 2-oxo-glutarate complex)**	**-1,8**	0,000077
1427414_at	Prkar2a	**protein kinase, cAMP dependent regulatory, type II alpha**	**-1,8**	0,000015
1435460_at	Prkg2	**protein kinase, cGMP-dependent, type II**	**-1,8**	0,013502
1434922_at	Phf12	**PHD finger protein 12**	**-1,8**	0,000026
1436508_at	Mlec	**malectin**	**-1,8**	0,000009
1448780_at	Slc12a2	**solute carrier family 12, member 2**	**-1,8**	0,003922
1454881_s_at	Upk3b	**uroplakin 3B**	**-1,8**	0,005580
1458703_at			**-1,8**	0,000194
1455874_at	Tmem179b	**transmembrane protein 179B**	**-1,8**	0,002335
1416959_at	Nr1d2	**nuclear receptor subfamily 1, group D, member 2**	**-1,8**	0,000328
1435349_at	Nrp2	**neuropilin 2**	**-1,8**	0,000558
1417293_at	Hs6st1	**heparan sulfate 6-O-sulfotransferase 1**	**-1,8**	0,000002
1450382_at	Nf2	**neurofibromatosis 2**	**-1,8**	0,000001
1449554_at	Tle3	**transducin-like enhancer of split 3, homolog of Drosophila E(spl)**	**-1,8**	0,000018
1449268_at	Gfpt1	**glutamine fructose-6-phosphate transaminase 1**	**-1,8**	0,000014
1440490_at			**-1,8**	0,000001
1427397_at	2810046L04Rik	**RIKEN cDNA 2810046L04 gene**	**-1,8**	0,000014
1443153_at			**-1,8**	0,000031
1444764_at	A130022J21Rik	**RIKEN cDNA A130022J21 gene**	**-1,8**	0,000023
1435905_at	D3Ertd300e	**DNA segment, Chr 3, ERATO Doi 300, expressed**	**-1,8**	0,000025
1439344_at			**-1,8**	0,000359
1435889_at	Mark2	**MAP/microtubule affinity-regulating kinase 2**	**-1,8**	0,000222
1417057_a_at	Ppid	**peptidylprolyl isomerase D (cyclophilin D)**	**-1,8**	0,000674
1446299_at			**-1,8**	0,000745
1435122_x_at	Dnmt1	**DNA methyltransferase (cytosine-5) 1**	**-1,8**	0,009088
1428065_at	Slc44a2	**solute carrier family 44, member 2**	**-1,8**	0,000125
1438208_at	Taok2	**TAO kinase 2**	**-1,8**	0,000008
1422681_at	Ctr9	**Ctr9, Paf1/RNA polymerase II complex component, homolog (S. cerevisiae)**	**-1,8**	0,000015
1417476_at	Fbxw5	**F-box and WD-40 domain protein 5**	**-1,8**	0,000011
1447034_at			**-1,8**	0,002261
1426381_at	Pprc1	**peroxisome proliferative activated receptor, gamma, coactivator-related 1**	**-1,8**	0,001866
1460259_s_at	Clca2	**chloride channel calcium activated 2**	**-1,8**	0,000009
1444785_at			**-1,8**	0,000094
1435338_at	Cdk6	**cyclin-dependent kinase 6**	**-1,8**	0,000631
1438888_at	Gmcl1	**germ cell-less homolog 1 (Drosophila)**	**-1,8**	0,000255
1424048_a_at	Cyb5r1	**cytochrome b5 reductase 1**	**-1,8**	0,000032
1452237_at	Agfg1	**ArfGAP with FG repeats 1**	**-1,8**	0,002759
1436524_at	4833438C02Rik	**RIKEN cDNA 4833438C02 gene**	**-1,8**	0,000007
1454106_a_at	Cxxc1	**CXXC finger 1 (PHD domain)**	**-1,8**	0,000002

1424556_at	Pycr1	**pyrroline-5-carboxylate reductase 1**	**-1,8**	0,002415
1431530_a_at	Tspan5	**tetraspanin 5**	**-1,8**	0,000135
1439572_at			**-1,8**	0,001152
1422474_at	Pde4b	**phosphodiesterase 4B, cAMP specific**	**-1,8**	0,000361
1425993_a_at	Hsph1	**heat shock 105kDa/110kDa protein 1**	**-1,8**	0,006020
1457339_at			**-1,8**	0,001687
1419838_s_at	Plk4	**polo-like kinase 4 (Drosophila)**	**-1,8**	0,013632
1426117_a_at	Slc19a2	**solute carrier family 19 (thiamine transporter), member 2**	**-1,8**	0,000009
1419248_at	Rgs2	**regulator of G-protein signaling 2**	**-1,8**	0,000197
1442171_at	Prpf40a	**PRP40 pre-mRNA processing factor 40 homolog A (yeast)**	**-1,8**	0,000252
1460113_at	B930093H17Rik	**RIKEN cDNA B930093H17 gene**	**-1,8**	0,000005
1439931_at	Gsk3b	**glycogen synthase kinase 3 beta**	**-1,8**	0,000012
1428850_x_at	Cd99	**CD99 antigen**	**-1,8**	0,000007
1439305_at			**-1,8**	0,000026
1451956_a_at	Sigmar1	**sigma non-opioid intracellular receptor 1**	**-1,8**	0,000007
1433751_at	Slc39a10	**solute carrier family 39 (zinc transporter), member 10**	**-1,8**	0,010990
1424375_s_at	Gimap4	**GTPase, IMAP family member 4**	**-1,8**	0,009208
1437283_at	Tnpo2	**transportin 2 (importin 3, karyopherin beta 2b)**	**-1,8**	0,000000
1438916_x_at	6720401G13Rik	**RIKEN cDNA 6720401G13 gene**	**-1,8**	0,005139
1418727_at	Nup155	**nucleoporin 155**	**-1,8**	0,009575
1439773_at	Ly6e	**lymphocyte antigen 6 complex, locus E**	**-1,8**	0,004133
1423430_at	Mybbp1a	**MYB binding protein (P160) 1a**	**-1,8**	0,000042
1441482_at	Map2k6	**mitogen-activated protein kinase kinase 6**	**-1,8**	0,012957
1443115_at			**-1,8**	0,001503
1444231_at			**-1,8**	0,000892
1454817_at	Utp18	**UTP18, small subunit (SSU) processome component, homolog (yeast)**	**-1,8**	0,004522
1438802_at	Foxp1	**forkhead box P1**	**-1,8**	0,000580
1424760_a_at	Smyd2	**SET and MYND domain containing 2**	**-1,8**	0,000319
1437667_a_at	Bach2	**BTB and CNC homology 2**	**-1,8**	0,000086
1452905_at	Meg3	**maternally expressed 3**	**-1,8**	0,001167
1451104_a_at	Snrnp70	**small nuclear ribonucleoprotein 70 (U1)**	**-1,8**	0,001109
1416295_a_at	Il2rg	**interleukin 2 receptor, gamma chain**	**-1,8**	0,005165
1420505_a_at	Stxbp1	**syntaxin binding protein 1**	**-1,8**	0,000097
1451115_at	Pias3	**protein inhibitor of activated STAT 3**	**-1,8**	0,000003
1434031_at	Zfp692	**zinc finger protein 692**	**-1,8**	0,000115
1432372_a_at	Spr	**sepiapterin reductase**	**-1,8**	0,000029
1419185_a_at	Mlxipl	**MLX interacting protein-like**	**-1,8**	0,000007
1431031_at	Arid4b	**AT rich interactive domain 4B (RBP1-like)**	**-1,8**	0,000311
1444150_at	Epb4.1	**erythrocyte protein band 4.1**	**-1,8**	0,000135
1441145_at	Phf21a	**PHD finger protein 21A**	**-1,8**	0,002293
1430920_at	Trmt11	**tRNA methyltransferase 11 homolog (S. cerevisiae)**	**-1,8**	0,000019

1442538_at			-1,8	0,000296
1455269_a_at	Coro1a	coronin, actin binding protein 1A	-1,8	0,009756
1456758_at	9930017N22Rik	RIKEN cDNA 9930017N22 gene	-1,8	0,000014
1428281_at	Trub1	TruB pseudouridine (psi) synthase homolog 1 (E. coli)	-1,8	0,000051
1458648_at	AU042950	expressed sequence AU042950	-1,8	0,000049
1456659_at	LOC552902	hypothetical LOC552902	-1,8	0,001805
1457047_at			-1,8	0,000981
1424544_at	Nrbp2	nuclear receptor binding protein 2	-1,9	0,000183
1443755_at			-1,9	0,000039
1423831_at	Prkag2	protein kinase, AMP-activated, gamma 2 non-catalytic subunit	-1,9	0,000013
1448881_at	Hp	haptoglobin	-1,9	0,005092
1434296_at	BC049349	cDNA sequence BC049349	-1,9	0,000012
1447096_at			-1,9	0,001918
1442309_at			-1,9	0,000034
1423723_s_at	Tardbp	TAR DNA binding protein	-1,9	0,004280
1451799_at	Ccdc25	coiled-coil domain containing 25	-1,9	0,000296
1458426_at			-1,9	0,000289
1438751_at	Slc30a10	solute carrier family 30, member 10	-1,9	0,001175
1448096_at	Ogfod1	2-oxoglutarate and iron-dependent oxygenase domain containing 1	-1,9	0,000826
1455521_at	Klf12	Kruppel-like factor 12	-1,9	0,002764
1448646_at	Wdr12	WD repeat domain 12	-1,9	0,002206
1424040_at	Mtap7d1	microtubule-associated protein 7 domain containing 1	-1,9	0,000015
1446474_at			-1,9	0,000007
1459701_x_at			-1,9	0,000019
1443489_at			-1,9	0,000070
1431118_at	6720427H10Rik	RIKEN cDNA 6720427H10 gene	-1,9	0,000009
1434847_at	Cnnm4	cyclin M4	-1,9	0,000627
1416184_s_at	Hmga1	high mobility group AT-hook 1	-1,9	0,000003
1457746_at			-1,9	0,000005
1452154_at	Iars	isoleucine-tRNA synthetase	-1,9	0,000848
1448346_at	Cfl1	cofilin 1, non-muscle	-1,9	0,000002
1457913_at	5730601F06Rik	RIKEN cDNA 5730601F06 gene	-1,9	0,011078
1427708_a_at	Nf2	neurofibromatosis 2	-1,9	0,000663
1432108_at	Pcgf6	polycomb group ring finger 6	-1,9	0,001312
1443167_at			-1,9	0,000982
1440799_s_at	Farp2	FERM, RhoGEF and pleckstrin domain protein 2	-1,9	0,000384
1416484_at	Ttc3	tetratricopeptide repeat domain 3	-1,9	0,000505
1445867_at	AL023008	expressed sequence AL023008	-1,9	0,000084
1415836_at	Aldh18a1	aldehyde dehydrogenase 18 family, member A1	-1,9	0,000002
1433806_x_at	Calr	calreticulin	-1,9	0,000004
1424927_at	Glipr1	GLI pathogenesis-related 1 (glioma)	-1,9	0,012266

1447929_at	Ssh3	**slingshot homolog 3 (Drosophila)**	**-1,9**	0,000000
1427978_at	4732418C07Rik	**RIKEN cDNA 4732418C07 gene**	**-1,9**	0,000000
1460096_at			**-1,9**	0,000042
1417655_a_at	Ars2	**arsenate resistance protein 2**	**-1,9**	0,000022
1456328_at	Bank1	**B-cell scaffold protein with ankyrin repeats 1**	**-1,9**	0,007419
1426533_at	Nop56	**NOP56 ribonucleoprotein homolog (yeast)**	**-1,9**	0,004610
1457356_at			**-1,9**	0,000244
1444372_at			**-1,9**	0,000092
1440464_at	Elavl1	**ELAV (embryonic lethal, abnormal vision, Drosophila)-like 1 (Hu antigen R)**	**-1,9**	0,001259
1443624_at			**-1,9**	0,000344
1418167_at	Tcfap4	**transcription factor AP4**	**-1,9**	0,000001
1423161_s_at	Spred1	**sprouty protein with EVH-1 domain 1, related sequence**	**-1,9**	0,000092
1442239_at			**-1,9**	0,000125
1456514_at			**-1,9**	0,000117
1436729_at	Afap1	**actin filament associated protein 1**	**-1,9**	0,000979
1447122_at	Psmd1	**proteasome (prosome, macropain) 26S subunit, non-ATPase, 1**	**-1,9**	0,000228
1436456_at	Slc38a9	**solute carrier family 38, member 9**	**-1,9**	0,000630
1456916_at	Nsd1	**nuclear receptor-binding SET-domain protein 1**	**-1,9**	0,000736
1444506_at			**-1,9**	0,001276
1423143_at	Gtpbp4	**GTP binding protein 4**	**-1,9**	0,001747
1430388_a_at	Sulf2	**sulfatase 2**	**-1,9**	0,000006
1425634_a_at	Tnk1	**tyrosine kinase, non-receptor, 1**	**-1,9**	0,000024
1416871_at	Adam8	**a disintegrin and metallopeptidase domain 8**	**-1,9**	0,015271
1448019_at	Taok3	**TAO kinase 3**	**-1,9**	0,000254
1437917_at	D530037H12Rik	**RIKEN cDNA D530037H12 gene**	**-1,9**	0,000418
1448221_at	Bat1a	**HLA-B-associated transcript 1A**	**-1,9**	0,000071
1428207_at	Bcl7a	**B-cell CLL/lymphoma 7A**	**-1,9**	0,005081
1435381_at	2610110G12Rik	**RIKEN cDNA 2610110G12 gene**	**-1,9**	0,000605
1421958_at	L1cam	**L1 cell adhesion molecule**	**-1,9**	0,000799
1443554_at	Ssbp3	**single-stranded DNA binding protein 3**	**-1,9**	0,003256
1442473_at			**-1,9**	0,001107
1420881_at	Acd	**adrenocortical dysplasia**	**-1,9**	0,000001
1458309_at			**-1,9**	0,000012
1434118_at	Mul1	**mitochondrial ubiquitin ligase activator of NFKB 1**	**-1,9**	0,000019
1433491_at	Epb4.1l2	**erythrocyte protein band 4.1-like 2**	**-1,9**	0,000923
1418070_at	Cdyl	**chromodomain protein, Y chromosome-like**	**-1,9**	0,000010
1457302_at			**-1,9**	0,002339
1442632_at			**-1,9**	0,001868
1450854_at	Pa2g4	**proliferation-associated 2G4**	**-1,9**	0,000402
1446001_at			**-1,9**	0,003211
1458618_at			**-1,9**	0,000144

1434908_at	Scaf1	**SR-related CTD-associated factor 1**	**-1,9**	0,000014
1417381_at	C1qa	**complement component 1, q subcomponent, alpha polypeptide**	**-1,9**	0,009906
1428772_at	Xpot	**exportin, tRNA (nuclear export receptor for tRNAs)**	**-1,9**	0,001310
1419032_at	Papola	**poly (A) polymerase alpha**	**-1,9**	0,000144
1441558_at	D230044B12Rik	**RIKEN cDNA D230044B12 gene**	**-1,9**	0,000351
1416814_at	Tia1	**cytotoxic granule-associated RNA binding protein 1**	**-1,9**	0,008406
1454665_at	Irf2bp2	**interferon regulatory factor 2 binding protein 2**	**-1,9**	0,000985
1446861_at	Gns	**glucosamine (N-acetyl)-6-sulfatase**	**-1,9**	0,000035
1450704_at	Ihh	**Indian hedgehog**	**-1,9**	0,000011
1450839_at	D0H4S114	**DNA segment, human D4S114**	**-1,9**	0,003908
1434191_at	Tmem195	**transmembrane protein 195**	**-1,9**	0,004079
1457072_at	Bcl11a	**B-cell CLL/lymphoma 11A (zinc finger protein)**	**-1,9**	0,010050
1457369_at			**-1,9**	0,000000
1445340_at	Mycbp2	**MYC binding protein 2**	**-1,9**	0,004028
1427322_at	Brwd1	**bromodomain and WD repeat domain containing 1**	**-1,9**	0,000486
1415798_at	Ddr1	**discoidin domain receptor family, member 1**	**-1,9**	0,000017
1419655_at	Tle3	**transducin-like enhancer of split 3, homolog of Drosophila E(spl)**	**-1,9**	0,000009
1421026_at	Gna12	**guanine nucleotide binding protein, alpha 12**	**-1,9**	0,000201
1427883_a_at	Col3a1	**collagen, type III, alpha 1**	**-1,9**	0,010875
1457593_at	2610202C22Rik	**RIKEN cDNA 2610202C22 gene**	**-1,9**	0,000018
1442916_at			**-1,9**	0,000798
1416614_at	Eid1	**EP300 interacting inhibitor of differentiation 1**	**-1,9**	0,000005
1452690_at	Khsrp	**KH-type splicing regulatory protein**	**-1,9**	0,000003
1449944_a_at	Sec61a2	**Sec61, alpha subunit 2 (S. cerevisiae)**	**-1,9**	0,000067
1452560_a_at	Nfya	**nuclear transcription factor-Y alpha**	**-1,9**	0,000009
1453399_at	Ccnt2	**cyclin T2**	**-1,9**	0,000011
1431993_a_at	Rnf38	**ring finger protein 38**	**-1,9**	0,000008
1448375_at	Tm9sf3	**transmembrane 9 superfamily member 3**	**-1,9**	0,000002
1448117_at	Kitl	**kit ligand**	**-1,9**	0,000121
1449202_at	Sema4g	**sema domain, immunoglobulin domain (Ig), transmembrane domain (TM) and short cytoplasmic domain, (**	**-1,9**	0,000001
1425233_at	2210407C18Rik	**RIKEN cDNA 2210407C18 gene**	**-1,9**	0,010084
1452336_at	Zfp395	**zinc finger protein 395**	**-1,9**	0,000002
1452377_at	Mll1	**myeloid/lymphoid or mixed-lineage leukemia 1**	**-1,9**	0,000293
1450116_at	D3Ertd300e	**DNA segment, Chr 3, ERATO Doi 300, expressed**	**-1,9**	0,000131
1418893_at	Pbx2	**pre B-cell leukemia transcription factor 2**	**-1,9**	0,000001
1442150_at			**-1,9**	0,000058
1450757_at	Cdh11	**cadherin 11**	**-1,9**	0,001662
1448617_at	Cd53	**CD53 antigen**	**-1,9**	0,013069
1437500_at	Noc3l	**nucleolar complex associated 3 homolog (S. cerevisiae)**	**-1,9**	0,000027
1454254_s_at	1600029D21Rik	**RIKEN cDNA 1600029D21 gene**	**-1,9**	0,003318
1437188_at	Gabbr1	**gamma-aminobutyric acid (GABA-B) receptor, 1**	**-1,9**	0,000008

1445201_at	Zfp53	**zinc finger protein 53**	**-1,9**	0,000905
1420984_at	Pctp	**phosphatidylcholine transfer protein**	**-1,9**	0,000029
1426501_a_at	Tifa	**TRAF-interacting protein with forkhead-associated domain**	**-1,9**	0,000568
1460205_at	Dcakd	**dephospho-CoA kinase domain containing**	**-1,9**	0,000010
1418202_a_at	Wiz	**widely-interspaced zinc finger motifs**	**-1,9**	0,000001
1450986_at	Nop58	**NOP58 ribonucleoprotein homolog (yeast)**	**-1,9**	0,008738
1456170_x_at	Calr	**calreticulin**	**-1,9**	0,000000
1438058_s_at	Ptov1	**prostate tumor over expressed gene 1**	**-1,9**	0,000000
1426458_at	Slmap	**sarcolemma associated protein**	**-1,9**	0,000007
1441684_at	Ttc3	**tetratricopeptide repeat domain 3**	**-1,9**	0,003045
1420834_at	Vamp2	**vesicle-associated membrane protein 2**	**-1,9**	0,000003
1422321_a_at	Sf1	**splicing factor 1**	**-1,9**	0,000001
1427099_at	Maz	**MYC-associated zinc finger protein (purine-binding transcription factor)**	**-1,9**	0,000001
1458233_at	Fryl	**furry homolog-like (Drosophila)**	**-1,9**	0,001041
1420924_at	Timp2	**tissue inhibitor of metalloproteinase 2**	**-1,9**	0,000909
1422440_at	Cdk4	**cyclin-dependent kinase 4**	**-1,9**	0,008642
1455831_at	Fus	**fusion, derived from t(12;16) malignant liposarcoma (human)**	**-1,9**	0,000554
1456715_at			**-1,9**	0,003593
1422850_at	Pabpn1	**poly(A) binding protein, nuclear 1**	**-1,9**	0,001545
1430356_at	2210402A03Rik	**RIKEN cDNA 2210402A03 gene**	**-1,9**	0,000007
1439545_at			**-1,9**	0,000020
1420824_at	Sema4d	**sema domain, immunoglobulin domain (Ig), transmembrane domain (TM) and short cytoplasmic domain, (**	**-1,9**	0,000011
1419276_at	Enpp1	**ectonucleotide pyrophosphatase/phosphodiesterase 1**	**-1,9**	0,000011
1426963_at	Pacs2	**phosphofurin acidic cluster sorting protein 2**	**-1,9**	0,000653
1444716_at	Mettl2	**methyltransferase like 2**	**-1,9**	0,000724
1455778_at	Zfp192	**zinc finger protein 192**	**-1,9**	0,001502
1420479_a_at	Nap1l1	**nucleosome assembly protein 1-like 1**	**-1,9**	0,009219
1459442_at			**-1,9**	0,000001
1459091_at			**-1,9**	0,000955
1435744_at	6720401G13Rik	**RIKEN cDNA 6720401G13 gene**	**-1,9**	0,003917
1453311_at	2310008B10Rik	**RIKEN cDNA 2310008B10 gene**	**-1,9**	0,000679
1420619_a_at	Aes	**amino-terminal enhancer of split**	**-1,9**	0,000000
1456526_at	C130034I24Rik	**RIKEN cDNA C130034I24 gene**	**-1,9**	0,000126
1415983_at	Lcp1	**lymphocyte cytosolic protein 1**	**-1,9**	0,000005
1435520_at	Msi2	**Musashi homolog 2 (Drosophila)**	**-1,9**	0,000005
1451229_at	Hdac11	**histone deacetylase 11**	**-1,9**	0,000021
1448127_at	Rrm1	**ribonucleotide reductase M1**	**-1,9**	0,010715
1437000_at	Dgkq	**diacylglycerol kinase, theta**	**-1,9**	0,000009
1417015_at	Rassf3	**Ras association (RalGDS/AF-6) domain family member 3**	**-1,9**	0,000015
1437078_at	Vps52	**vacuolar protein sorting 52 (yeast)**	**-1,9**	0,000108
1454142_a_at	Pwp1	**PWP1 homolog (S. cerevisiae)**	**-1,9**	0,000047

1429246_a_at	Anxa6	**annexin A6**	**-1,9**	0,012762
1449401_at	C1qc	**complement component 1, q subcomponent, C chain**	**-1,9**	0,008703
1426538_a_at	Trp53	**transformation related protein 53**	**-1,9**	0,000001
1440381_at	2410085M17Rik	**RIKEN cDNA 2410085M17 gene**	**-1,9**	0,000408
1421355_at	Tgm3	**transglutaminase 3, E polypeptide**	**-1,9**	0,000069
1423162_s_at	Spred1	**sprouty protein with EVH-1 domain 1, related sequence**	**-2,0**	0,000001
1453414_at	Ypel2	**yippee-like 2 (Drosophila)**	**-2,0**	0,000281
1453988_a_at	Ide	**insulin degrading enzyme**	**-2,0**	0,000005
1446598_at			**-2,0**	0,000166
1442761_at			**-2,0**	0,000273
1424794_at	Rnf186	**ring finger protein 186**	**-2,0**	0,000291
1421070_at	D3Ertd300e	**DNA segment, Chr 3, ERATO Doi 300, expressed**	**-2,0**	0,000299
1431362_a_at	Smoc2	**SPARC related modular calcium binding 2**	**-2,0**	0,008301
1449120_a_at	Pcm1	**pericentriolar material 1**	**-2,0**	0,000002
1430177_at	Ube2b	**ubiquitin-conjugating enzyme E2B, RAD6 homology (S. cerevisiae)**	**-2,0**	0,000571
1454268_a_at	Cyba	**cytochrome b-245, alpha polypeptide**	**-2,0**	0,000417
1445387_at	Senp6	**SUMO/sentrin specific peptidase 6**	**-2,0**	0,000036
1424281_at	Ubap2	**ubiquitin-associated protein 2**	**-2,0**	0,000000
1457793_a_at	Whsc1l1	**Wolf-Hirschhorn syndrome candidate 1-like 1 (human)**	**-2,0**	0,000001
1449029_at	Mknk2	**MAP kinase-interacting serine/threonine kinase 2**	**-2,0**	0,000000
1451034_at	Zfp36l2	**zinc finger protein 36, C3H type-like 2**	**-2,0**	0,000004
1447402_at			**-2,0**	0,000011
1442484_at	D9Ertd306e	**DNA segment, Chr 9, ERATO Doi 306, expressed**	**-2,0**	0,000468
1450914_at	Ppp1r14b	**protein phosphatase 1, regulatory (inhibitor) subunit 14B**	**-2,0**	0,001316
1441956_s_at	Cux1	**cut-like homeobox 1**	**-2,0**	0,000677
1453074_at	Dusp23	**dual specificity phosphatase 23**	**-2,0**	0,000259
1436902_x_at	Tmsb10	**thymosin, beta 10**	**-2,0**	0,000001
1456904_at			**-2,0**	0,000145
1428869_at	Nolc1	**nucleolar and coiled-body phosphoprotein 1**	**-2,0**	0,000527
1443534_at			**-2,0**	0,000078
1434804_at	Exoc6b	**exocyst complex component 6B**	**-2,0**	0,000004
1456223_at			**-2,0**	0,000095
1429477_at	Ncaph2	**non-SMC condensin II complex, subunit H2**	**-2,0**	0,000704
1452226_at	Rcc2	**regulator of chromosome condensation 2**	**-2,0**	0,004644
1423757_x_at	Igfbp4	**insulin-like growth factor binding protein 4**	**-2,0**	0,013663
1416572_at	Mmp14	**matrix metallopeptidase 14 (membrane-inserted)**	**-2,0**	0,000266
1434166_at	9330151L19Rik	**RIKEN cDNA 9330151L19 gene**	**-2,0**	0,000520
1416362_a_at	Fkbp4	**FK506 binding protein 4**	**-2,0**	0,000051
1457848_at			**-2,0**	0,000002
1423746_at	Txndc5	**thioredoxin domain containing 5**	**-2,0**	0,000001
1457944_at			**-2,0**	0,000534

1449005_at	Slc16a3	**solute carrier family 16 (monocarboxylic acid transporters), member 3**	**-2,0**	0,000306
1416998_at	Rrs1	**RRS1 ribosome biogenesis regulator homolog (S. cerevisiae)**	**-2,0**	0,000101
1446621_at			**-2,0**	0,000093
1448748_at	Plek	**pleckstrin**	**-2,0**	0,012564
1424259_at	Lmf1	**lipase maturation factor 1**	**-2,0**	0,000011
1436427_at	Prpf4b	**PRP4 pre-mRNA processing factor 4 homolog B (yeast)**	**-2,0**	0,002367
1415977_at	Isyna1	**myo-inositol 1-phosphate synthase A1**	**-2,0**	0,000027
1416365_at	Hsp90ab1	**heat shock protein 90 alpha (cytosolic), class B member 1**	**-2,0**	0,000015
1417175_at	Csnk1e	**casein kinase 1, epsilon**	**-2,0**	0,000770
1439537_at			**-2,0**	0,000090
1429332_at	4632427E13Rik	**RIKEN cDNA 4632427E13 gene**	**-2,0**	0,000006
1459433_at	C130051F05Rik	**RIKEN cDNA C130051F05 gene**	**-2,0**	0,000139
1450377_at	Thbs1	**thrombospondin 1**	**-2,0**	0,000056
1424938_at	Steap1	**six transmembrane epithelial antigen of the prostate 1**	**-2,0**	0,004091
1435981_at	Nav2	**neuron navigator 2**	**-2,0**	0,000167
1430418_at	Tmem57	**transmembrane protein 57**	**-2,0**	0,000288
1458972_at	9330112F22Rik	**RIKEN cDNA 9330112F22 gene**	**-2,0**	0,000036
1421267_a_at	Cited2	**Cbp/p300-interacting transactivator, with Glu/Asp-rich carboxy-terminal domain, 2**	**-2,0**	0,000001
1439619_at	Tcf12	**transcription factor 12**	**-2,0**	0,002775
1452139_at	Slc35c1	**solute carrier family 35, member C1**	**-2,0**	0,000002
1446692_at			**-2,0**	0,000013
1417219_s_at	Tmsb10	**thymosin, beta 10**	**-2,0**	0,000000
1460116_s_at	Spred1	**sprouty protein with EVH-1 domain 1, related sequence**	**-2,0**	0,000065
1442418_at	B930096F20Rik	**RIKEN cDNA B930096F20 gene**	**-2,0**	0,000141
1434077_at	Wdr37	**WD repeat domain 37**	**-2,0**	0,000003
1448842_at	Cdo1	**cysteine dioxygenase 1, cytosolic**	**-2,0**	0,012853
1460210_at	Pkd1	**polycystic kidney disease 1 homolog**	**-2,0**	0,000000
1435162_at	Prkg2	**protein kinase, cGMP-dependent, type II**	**-2,0**	0,010413
1454247_a_at	Gpa33	**glycoprotein A33 (transmembrane)**	**-2,0**	0,000001
1446508_at			**-2,0**	0,000001
1419257_at	Tcea1	**transcription elongation factor A (SII) 1**	**-2,0**	0,000074
1443332_at			**-2,0**	0,004935
1460565_at	Slc41a1	**solute carrier family 41, member 1**	**-2,0**	0,000295
1440294_at			**-2,0**	0,000071
1434784_s_at	Tmem106c	**transmembrane protein 106C**	**-2,0**	0,000001
1451199_at	Qtrtd1	**queuine tRNA-ribosyltransferase domain containing 1**	**-2,0**	0,002877
1419522_at	Zmynd19	**zinc finger, MYND domain containing 19**	**-2,0**	0,000040
1429870_at	Tnik	**TRAF2 and NCK interacting kinase**	**-2,0**	0,006291
1436919_at	Trp53i11	**transformation related protein 53 inducible protein 11**	**-2,0**	0,000011
1422972_s_at	Kat2a	**K(lysine) acetyltransferase 2A**	**-2,0**	0,000001
1442056_at			**-2,0**	0,001717

1417658_at	Tbrg4	**transforming growth factor beta regulated gene 4**	**-2,0**	0,000001
1420653_at	Tgfb1	**transforming growth factor, beta 1**	**-2,0**	0,000026
1440615_at	Dusp16	**dual specificity phosphatase 16**	**-2,0**	0,000020
1431096_at	Ints8	**integrator complex subunit 8**	**-2,0**	0,000175
1454090_at	Pdss1	**prenyl (solanesyl) diphosphate synthase, subunit 1**	**-2,0**	0,000000
1420833_at	Vamp2	**vesicle-associated membrane protein 2**	**-2,0**	0,000016
1419168_at	Mapk6	**mitogen-activated protein kinase 6**	**-2,0**	0,000000
1444042_at	Trip11	**thyroid hormone receptor interactor 11**	**-2,0**	0,000003
1423325_at	Pnn	**pinin**	**-2,0**	0,000193
1449080_at	Hdac2	**histone deacetylase 2**	**-2,0**	0,002875
1457188_at			**-2,0**	0,000012
1417814_at	Pla2g5	**phospholipase A2, group V**	**-2,0**	0,000185
1428557_a_at	Ormdl1	**ORM1-like 1 (S. cerevisiae)**	**-2,0**	0,000437
1452427_s_at	Ptplad1	**protein tyrosine phosphatase-like A domain containing 1**	**-2,0**	0,000129
1438686_at	Eif4g1	**eukaryotic translation initiation factor 4, gamma 1**	**-2,0**	0,000001
1431061_s_at	Peli1	**pellino 1**	**-2,0**	0,000000
1458065_at			**-2,0**	0,000239
1455548_at	Dlgap4	**discs, large homolog-associated protein 4 (Drosophila)**	**-2,0**	0,000000
1421224_a_at	Hnf1b	**HNF1 homeobox B**	**-2,0**	0,000000
1450747_at	Keap1	**kelch-like ECH-associated protein 1**	**-2,0**	0,000032
1436871_at	Sfrs7	**splicing factor, arginine/serine-rich 7**	**-2,0**	0,003940
1429088_at	Lbh	**limb-bud and heart**	**-2,0**	0,000001
1460218_at	Cd52	**CD52 antigen**	**-2,0**	0,003816
1424198_at	Dlg5	**discs, large homolog 5 (Drosophila)**	**-2,0**	0,000010
1448590_at	Col6a1	**collagen, type VI, alpha 1**	**-2,0**	0,001071
1439123_at			**-2,0**	0,000202
1433586_at	Rgmb	**RGM domain family, member B**	**-2,0**	0,000005
1436318_at	Tardbp	**TAR DNA binding protein**	**-2,0**	0,000556
1443846_x_at			**-2,0**	0,000015
1436232_a_at	Gabpb1	**GA repeat binding protein, beta 1**	**-2,0**	0,000000
1444445_at	C77648	**expressed sequence C77648**	**-2,0**	0,000086
1421811_at	Thbs1	**thrombospondin 1**	**-2,0**	0,006431
1445827_at	Prkcbp1	**protein kinase C binding protein 1**	**-2,0**	0,000206
1445178_at	Sh3rf1	**SH3 domain containing ring finger 1**	**-2,0**	0,002888
1456063_at	Fam120c	**family with sequence similarity 120, member C**	**-2,0**	0,000102
1423087_a_at	Tomm6	**translocase of outer mitochondrial membrane 6 homolog (yeast)**	**-2,0**	0,000000
1451331_at	Ppp1r1b	**protein phosphatase 1, regulatory (inhibitor) subunit 1B**	**-2,0**	0,000001
1417253_at	Frg1	**FSHD region gene 1**	**-2,0**	0,000000
1448603_at	Srpk2	**serine/arginine-rich protein specific kinase 2**	**-2,0**	0,000001
1424668_a_at	Cux1	**cut-like homeobox 1**	**-2,0**	0,000000
1423121_at	Ide	**insulin degrading enzyme**	**-2,0**	0,000001

1443355_at			**-2,0**	0,000636
1422249_s_at	Zfa	**zinc finger protein, autosomal**	**-2,0**	0,000612
1428142_at	Etv5	**ets variant gene 5**	**-2,0**	0,010199
1423004_at	Vipr1	**vasoactive intestinal peptide receptor 1**	**-2,0**	0,000153
1440040_at			**-2,0**	0,003431
1441359_at	Zcchc11	**zinc finger, CCHC domain containing 11**	**-2,0**	0,000012
1441505_at			**-2,1**	0,000049
1427408_a_at	Thrap3	**thyroid hormone receptor associated protein 3**	**-2,1**	0,000000
1441125_at	Setd5	**SET domain containing 5**	**-2,1**	0,000039
1436321_at	B3gnt7	**UDP-GlcNAc:betaGal beta-1,3-N-acetylglucosaminyltransferase 7**	**-2,1**	0,000292
1434644_at	Tbl1x	**transducin (beta)-like 1 X-linked**	**-2,1**	0,000006
1457262_at	Smg1	**SMG1 homolog, phosphatidylinositol 3-kinase-related kinase (C. elegans)**	**-2,1**	0,000000
1417232_at	Cldn2	**claudin 2**	**-2,1**	0,000321
1444333_at			**-2,1**	0,000100
1420699_at	Clec7a	**C-type lectin domain family 7, member a**	**-2,1**	0,002664
1457812_at	Trp53bp1	**transformation related protein 53 binding protein 1**	**-2,1**	0,005441
1453234_at	1300002K09Rik	**RIKEN cDNA 1300002K09 gene**	**-2,1**	0,000033
1441475_at			**-2,1**	0,000131
1451884_a_at	Lsm2	**LSM2 homolog, U6 small nuclear RNA associated (S. cerevisiae)**	**-2,1**	0,000000
1422528_a_at	Zfp36l1	**zinc finger protein 36, C3H type-like 1**	**-2,1**	0,000000
1445341_at			**-2,1**	0,000227
1423357_at	2610209A20Rik	**RIKEN cDNA 2610209A20 gene**	**-2,1**	0,001263
1441272_at	Matr3	**matrin 3**	**-2,1**	0,000029
1428069_at	Cdca7	**cell division cycle associated 7**	**-2,1**	0,002715
1446182_at			**-2,1**	0,000103
1449845_a_at	Ephb4	**Eph receptor B4**	**-2,1**	0,000000
1456955_at			**-2,1**	0,000029
1431235_at	Matr3	**matrin 3**	**-2,1**	0,000000
1427048_at	Smo	**smoothened homolog (Drosophila)**	**-2,1**	0,000069
1440372_at			**-2,1**	0,000021
1438700_at	Fnbp4	**formin binding protein 4**	**-2,1**	0,000193
1415871_at	Tgfbi	**transforming growth factor, beta induced**	**-2,1**	0,000038
1420762_a_at	Ybx2	**Y box protein 2**	**-2,1**	0,000000
1422705_at	Pmepa1	**prostate transmembrane protein, androgen induced 1**	**-2,1**	0,006721
1424081_at	Pcgf6	**polycomb group ring finger 6**	**-2,1**	0,000316
1442224_at			**-2,1**	0,000022
1446143_at			**-2,1**	0,000002
1436515_at	Bach2	**BTB and CNC homology 2**	**-2,1**	0,000072
1440123_at			**-2,1**	0,000007
1418824_at	Arf6	**ADP-ribosylation factor 6**	**-2,1**	0,000007
1422854_at	Shc1	**src homology 2 domain-containing transforming protein C1**	**-2,1**	0,000003

1421118_a_at	Gpr56	**G protein-coupled receptor 56**	**-2,1**	0,000000
1434554_at	Trim37	**tripartite motif-containing 37**	**-2,1**	0,001400
1437798_at	6720422M22Rik	**RIKEN cDNA 6720422M22 gene**	**-2,1**	0,003899
1452899_at	Rian	**RNA imprinted and accumulated in nucleus**	**-2,1**	0,000419
1443444_at			**-2,1**	0,000012
1440254_at	100041277	**predicted gene, 100041277**	**-2,1**	0,001202
1449551_at	Myo1c	**myosin IC**	**-2,1**	0,000000
1431830_at	Zfp329	**zinc finger protein 329**	**-2,1**	0,000160
1456547_at			**-2,1**	0,000049
1420946_at	Atrx	**alpha thalassemia/mental retardation syndrome X-linked homolog (human)**	**-2,1**	0,000899
1438069_a_at	Rbm5	**RNA binding motif protein 5**	**-2,1**	0,000356
1433485_x_at	Gpr56	**G protein-coupled receptor 56**	**-2,1**	0,000000
1418835_at	Phlda1	**pleckstrin homology-like domain, family A, member 1**	**-2,1**	0,008178
1450089_a_at	Srprb	**signal recognition particle receptor, B subunit**	**-2,1**	0,000000
1430837_a_at	Mbd1	**methyl-CpG binding domain protein 1**	**-2,1**	0,000000
1425711_a_at	Akt1	**thymoma viral proto-oncogene 1**	**-2,1**	0,000000
1417632_at	Atp6v0a1	**ATPase, H+ transporting, lysosomal V0 subunit A1**	**-2,1**	0,000001
1444890_at			**-2,1**	0,000464
1427739_a_at	Trp53	**transformation related protein 53**	**-2,1**	0,000001
1418830_at	Cd79a	**CD79A antigen (immunoglobulin-associated alpha)**	**-2,1**	0,004599
1452811_at	Atic	**5-aminoimidazole-4-carboxamide ribonucleotide formyltransferase/IMP cyclohydrolase**	**-2,1**	0,000000
1431175_at	1810019D21Rik	**RIKEN cDNA 1810019D21 gene**	**-2,1**	0,000467
1436736_x_at	D0H4S114	**DNA segment, human D4S114**	**-2,1**	0,007208
1428319_at	Pdlim7	**PDZ and LIM domain 7**	**-2,1**	0,002488
1420975_at	Baz1b	**bromodomain adjacent to zinc finger domain, 1B**	**-2,1**	0,000001
1443158_at			**-2,1**	0,000017
1425276_at	Fbrs	**fibrosin**	**-2,1**	0,000000
1442336_at			**-2,2**	0,000000
1442316_x_at	Trp53bp1	**transformation related protein 53 binding protein 1**	**-2,2**	0,000155
1420893_a_at	Tgfbr1	**transforming growth factor, beta receptor I**	**-2,2**	0,000027
1460279_a_at	Gtf2i	**general transcription factor II I**	**-2,2**	0,000027
1428513_at	Calcoco1	**calcium binding and coiled coil domain 1**	**-2,2**	0,000000
1435477_s_at	Fcgr2b	**Fc receptor, IgG, low affinity IIb**	**-2,2**	0,004341
1446618_at	LOC432971	**hypothetical gene supported by AK038224**	**-2,2**	0,000009
1437431_at	Cux1	**cut-like homeobox 1**	**-2,2**	0,000057
1439948_at	BC046401	**cDNA sequence BC046401**	**-2,2**	0,000626
1443509_at			**-2,2**	0,000058
1450743_s_at	Syncrip	**synaptotagmin binding, cytoplasmic RNA interacting protein**	**-2,2**	0,000197
1434852_at	Pla2g2f	**phospholipase A2, group IIF**	**-2,2**	0,000005
1442700_at	Pde4b	**phosphodiesterase 4B, cAMP specific**	**-2,2**	0,001242
1456849_at			**-2,2**	0,000025

1438245_at	Nfib	**nuclear factor I/B**	**-2,2**	0,000039
1420747_at	Ppnr	**per-pentamer repeat gene**	**-2,2**	0,000198
1428909_at	A130040M12Rik	**RIKEN cDNA A130040M12 gene**	**-2,2**	0,000066
1439950_at	Dync1h1	**dynein cytoplasmic 1 heavy chain 1**	**-2,2**	0,000006
1438402_at	Fam171a1	**family with sequence similarity 171, member A1**	**-2,2**	0,000010
1427037_at	Eif4g1	**eukaryotic translation initiation factor 4, gamma 1**	**-2,2**	0,000000
1415922_s_at	Marcksl1	**MARCKS-like 1**	**-2,2**	0,008574
1455878_at	2700023E23Rik	**RIKEN cDNA 2700023E23 gene**	**-2,2**	0,004043
1448692_at	Ubqln4	**ubiquilin 4**	**-2,2**	0,000000
1448916_at	Mafg	**v-maf musculoaponeurotic fibrosarcoma oncogene family, protein G (avian)**	**-2,2**	0,000145
1439136_at			**-2,2**	0,000090
1456632_at	Bcl11a	**B-cell CLL/lymphoma 11A (zinc finger protein)**	**-2,2**	0,006576
1415824_at	Scd2	**stearoyl-Coenzyme A desaturase 2**	**-2,2**	0,000024
1445866_at	Mast4	**microtubule associated serine/threonine kinase family member 4**	**-2,2**	0,000004
1417326_a_at	Anapc11	**anaphase promoting complex subunit 11**	**-2,2**	0,000000
1426596_a_at	Smn1	**survival motor neuron 1**	**-2,2**	0,000000
1431206_at	5730601F06Rik	**RIKEN cDNA 5730601F06 gene**	**-2,2**	0,000424
1421354_at	Prkg2	**protein kinase, cGMP-dependent, type II**	**-2,2**	0,007489
1436967_at	Ankrd11	**ankyrin repeat domain 11**	**-2,2**	0,000029
1452021_a_at	Hes6	**hairy and enhancer of split 6 (Drosophila)**	**-2,2**	0,003228
1450379_at	Msn	**moesin**	**-2,2**	0,000014
1425359_at	Mall	**mal, T-cell differentiation protein-like**	**-2,2**	0,001021
1433587_at	Rgmb	**RGM domain family, member B**	**-2,2**	0,000030
1441373_at			**-2,2**	0,000512
1460672_at	2410002F23Rik	**RIKEN cDNA 2410002F23 gene**	**-2,2**	0,000592
1435608_at	Znrf3	**zinc and ring finger 3**	**-2,2**	0,012041
1459316_at			**-2,2**	0,000035
1448018_at			**-2,2**	0,001874
1445200_at			**-2,2**	0,000725
1453623_a_at	Rad23a	**RAD23a homolog (S. cerevisiae)**	**-2,2**	0,000000
1416106_at	Kti12	**KTI12 homolog, chromatin associated (S. cerevisiae)**	**-2,2**	0,000015
1425698_a_at	Crebzf	**CREB/ATF bZIP transcription factor**	**-2,2**	0,000002
1436869_at	Shh	**sonic hedgehog**	**-2,2**	0,001881
1458358_at	Pank2	**pantothenate kinase 2 (Hallervorden-Spatz syndrome)**	**-2,2**	0,000007
1441481_at	Mfap3l	**microfibrillar-associated protein 3-like**	**-2,2**	0,014794
1434316_at	Chsy1	**chondroitin sulfate synthase 1**	**-2,2**	0,001377
1447408_at			**-2,2**	0,000136
1422846_at	Rbp2	**retinol binding protein 2, cellular**	**-2,2**	0,000001
1442535_at			**-2,2**	0,000091
1428462_at	Ppp2r5e	**protein phosphatase 2, regulatory subunit B (B56), epsilon isoform**	**-2,3**	0,000002
1440841_at			**-2,3**	0,000062

1438164_x_at	Flot2	**flotillin 2**	**-2,3**	0,000035
1446550_at	Gspt1	**G1 to S phase transition 1**	**-2,3**	0,000319
1444260_at			**-2,3**	0,001108
1451169_at	Nomo1	**nodal modulator 1**	**-2,3**	0,000000
1440621_at			**-2,3**	0,000039
1438559_x_at	Slc44a2	**solute carrier family 44, member 2**	**-2,3**	0,000107
1458018_at			**-2,3**	0,000025
1439477_at	5430406J06Rik	**RIKEN cDNA 5430406J06 gene**	**-2,3**	0,000002
1419480_at	Sell	**selectin, lymphocyte**	**-2,3**	0,000182
1442992_at	130004C03	**hypothetical LOC403343**	**-2,3**	0,000326
1455060_at	G3bp1	**Ras-GTPase-activating protein SH3-domain binding protein 1**	**-2,3**	0,000008
1427110_at	Raver1	**ribonucleoprotein, PTB-binding 1**	**-2,3**	0,000004
1427797_s_at			**-2,3**	0,000005
1437486_at	Gprc5a	**G protein-coupled receptor, family C, group 5, member A**	**-2,3**	0,000007
1416200_at	Il33	**interleukin 33**	**-2,3**	0,006724
1418587_at	Traf3	**TNF receptor-associated factor 3**	**-2,3**	0,000000
1422559_at	Ube2n	**ubiquitin-conjugating enzyme E2N**	**-2,3**	0,000000
1456993_at	D2Ertd640e	**DNA segment, Chr 2, ERATO Doi 640, expressed**	**-2,3**	0,000016
1460302_at	Thbs1	**thrombospondin 1**	**-2,3**	0,000810
1429192_at	Ski	**ski sarcoma viral oncogene homolog (avian)**	**-2,3**	0,000000
1424667_a_at	Cux1	**cut-like homeobox 1**	**-2,3**	0,000000
1428694_at	5033413D16Rik	**RIKEN cDNA 5033413D16 gene**	**-2,3**	0,001549
1444140_at			**-2,3**	0,000180
1421709_a_at	Fmo5	**flavin containing monooxygenase 5**	**-2,3**	0,003287
1421164_a_at	Arhgef1	**Rho guanine nucleotide exchange factor (GEF) 1**	**-2,3**	0,000009
1440196_at			**-2,3**	0,000182
1442071_at	Abce1	**ATP-binding cassette, sub-family E (OABP), member 1**	**-2,3**	0,001526
1417460_at	Ifitm2	**interferon induced transmembrane protein 2**	**-2,3**	0,000999
1420948_s_at	Atrx	**alpha thalassemia/mental retardation syndrome X-linked homolog (human)**	**-2,3**	0,000397
1450834_at	Fut4	**fucosyltransferase 4**	**-2,3**	0,000000
1417426_at	Srgn	**serglycin**	**-2,3**	0,006102
1424117_at	BC056474	**cDNA sequence BC056474**	**-2,3**	0,000000
1442067_at			**-2,3**	0,000176
1423129_at	Shoc2	**soc-2 (suppressor of clear) homolog (C. elegans)**	**-2,3**	0,000000
1421149_a_at	Atn1	**atrophin 1**	**-2,3**	0,000000
1422439_a_at	Cdk4	**cyclin-dependent kinase 4**	**-2,3**	0,007011
1442903_at	4732423E21Rik	**RIKEN cDNA 4732423E21 gene**	**-2,4**	0,001925
1440773_at			**-2,4**	0,000003
1427903_at	Phpt1	**phosphohistidine phosphatase 1**	**-2,4**	0,000009
1427008_at	Rnf43	**ring finger protein 43**	**-2,4**	0,009681
1436222_at	Gas5	**growth arrest specific 5**	**-2,4**	0,009639

1450792_at	Tyrobp	**TYRO protein tyrosine kinase binding protein**	**-2,4**	0,000007
1417063_at	C1qb	**complement component 1, q subcomponent, beta polypeptide**	**-2,4**	0,007557
1437733_at	Eif4ebp2	**eukaryotic translation initiation factor 4E binding protein 2**	**-2,4**	0,000000
1428777_at	Spred1	**sprouty protein with EVH-1 domain 1, related sequence**	**-2,4**	0,000000
1423566_a_at	Hsph1	**heat shock 105kDa/110kDa protein 1**	**-2,4**	0,000379
1448797_at	Elk3	**ELK3, member of ETS oncogene family**	**-2,4**	0,011654
1448160_at	Lcp1	**lymphocyte cytosolic protein 1**	**-2,4**	0,000003
1418591_at	Dnaja4	**DnaJ (Hsp40) homolog, subfamily A, member 4**	**-2,4**	0,000196
1433756_at	S100pbp	**S100P binding protein**	**-2,4**	0,000046
1448766_at	Gjb1	**gap junction protein, beta 1**	**-2,4**	0,000001
1423760_at	Cd44	**CD44 antigen**	**-2,4**	0,007276
1441615_at	Cbfa2t2	**core-binding factor, runt domain, alpha subunit 2, translocated to, 2 (human)**	**-2,4**	0,000085
1458676_at	Nktr	**natural killer tumor recognition sequence**	**-2,4**	0,000001
1457383_at			**-2,4**	0,000000
1449956_at	Prkce	**protein kinase C, epsilon**	**-2,4**	0,000241
1420394_s_at	Lilrb4	**leukocyte immunoglobulin-like receptor, subfamily B, member 4**	**-2,4**	0,009649
1429169_at	Rbm3	**RNA binding motif protein 3**	**-2,4**	0,015359
1417821_at	D17H6S56E-5	**DNA segment, Chr 17, human D6S56E 5**	**-2,4**	0,005630
1420643_at	Lfng	**LFNG O-fucosylpeptide 3-beta-N-acetylglucosaminyltransferase**	**-2,4**	0,000009
1415935_at	Smoc2	**SPARC related modular calcium binding 2**	**-2,4**	0,008087
1447147_at			**-2,4**	0,003647
1452911_at	Spred1	**sprouty protein with EVH-1 domain 1, related sequence**	**-2,4**	0,000002
1437400_at	Nedd4l	**neural precursor cell expressed, developmentally down-regulated gene 4-like**	**-2,5**	0,000001
1452841_at	Pgm2l1	**phosphoglucomutase 2-like 1**	**-2,5**	0,011822
1421789_s_at	Arf3	**ADP-ribosylation factor 3**	**-2,5**	0,000033
1432646_a_at	2900097C17Rik	**RIKEN cDNA 2900097C17 gene**	**-2,5**	0,000022
1454904_at	Mtm1	**X-linked myotubular myopathy gene 1**	**-2,5**	0,001372
1422135_at	Zfp146	**zinc finger protein 146**	**-2,5**	0,000165
1429555_at	Cldnd1	**claudin domain containing 1**	**-2,5**	0,000004
1443095_at			**-2,5**	0,000059
1448710_at	Cxcr4	**chemokine (C-X-C motif) receptor 4**	**-2,5**	0,000002
1424179_at	Plekhj1	**pleckstrin homology domain containing, family J member 1**	**-2,5**	0,000000
1455904_at	Gas5	**growth arrest specific 5**	**-2,5**	0,008137
1442675_at			**-2,5**	0,002387
1436541_at	2310008H09Rik	**RIKEN cDNA 2310008H09 gene**	**-2,5**	0,000012
1418468_at	Anxa11	**annexin A11**	**-2,5**	0,000001
1437052_s_at	Slc2a3	**solute carrier family 2 (facilitated glucose transporter), member 3**	**-2,5**	0,004548
1417470_at	Apobec3	**apolipoprotein B mRNA editing enzyme, catalytic polypeptide 3**	**-2,5**	0,000168
1443027_at			**-2,5**	0,000386
1417228_at	Capn1	**calpain 1**	**-2,5**	0,000001
1459942_at			**-2,5**	0,000005

1448482_at	Slc39a8	**solute carrier family 39 (metal ion transporter), member 8**	**-2,5**	0,000037
1448749_at	Plek	**pleckstrin**	**-2,5**	0,000826
1445390_at	Pawr	**PRKC, apoptosis, WT1, regulator**	**-2,5**	0,000000
1455246_at	Smarcc1	**SWI/SNF related, matrix associated, actin dependent regulator of chromatin, subfamily c, member 1**	**-2,5**	0,000405
1437627_at	Mex3d	**mex3 homolog D (C. elegans)**	**-2,5**	0,000235
1428010_at	Timm9	**translocase of inner mitochondrial membrane 9 homolog (yeast)**	**-2,5**	0,000112
1452235_at	Man1b1	**mannosidase, alpha, class 1B, member 1**	**-2,6**	0,000000
1435655_at	Rpl12	**ribosomal protein L12**	**-2,6**	0,000424
1446130_at			**-2,6**	0,000266
1418524_at	Pcm1	**pericentriolar material 1**	**-2,6**	0,000002
1435152_at	Leng8	**leukocyte receptor cluster (LRC) member 8**	**-2,6**	0,000004
1416246_a_at	Coro1a	**coronin, actin binding protein 1A**	**-2,6**	0,000506
1459443_at			**-2,6**	0,000430
1457781_at	Kcnq1ot1	**KCNQ1 overlapping transcript 1**	**-2,6**	0,000585
1437325_x_at	Aldh18a1	**aldehyde dehydrogenase 18 family, member A1**	**-2,6**	0,000006
1431385_a_at	Mbtps1	**membrane-bound transcription factor peptidase, site 1**	**-2,6**	0,000002
1427747_a_at	Lcn2	**lipocalin 2**	**-2,6**	0,000740
1425716_s_at	Bak1	**BCL2-antagonist/killer 1**	**-2,6**	0,000003
1423754_at	Ifitm3	**interferon induced transmembrane protein 3**	**-2,6**	0,011114
1441797_at			**-2,6**	0,000021
1452483_a_at	Cd44	**CD44 antigen**	**-2,6**	0,000409
1448029_at	Tbx3	**T-box 3**	**-2,7**	0,002106
1455195_at	Rps24	**ribosomal protein S24**	**-2,7**	0,001936
1434357_a_at	Kpnb1	**karyopherin (importin) beta 1**	**-2,7**	0,000000
1443837_x_at	Bcl2	**B-cell leukemia/lymphoma 2**	**-2,7**	0,000362
1455740_at	Hnrnpa1	**heterogeneous nuclear ribonucleoprotein A1**	**-2,7**	0,000306
1421262_at	Lipg	**lipase, endothelial**	**-2,7**	0,000006
1419728_at	Cxcl5	**chemokine (C-X-C motif) ligand 5**	**-2,7**	0,005692
1430332_a_at	Gusb	**glucuronidase, beta**	**-2,7**	0,000122
1460650_at	Atp6v0a1	**ATPase, H+ transporting, lysosomal V0 subunit A1**	**-2,7**	0,000007
1434376_at	Cd44	**CD44 antigen**	**-2,7**	0,005388
1457084_at	Nlrp9b	**NLR family, pyrin domain containing 9B**	**-2,7**	0,000001
1419398_a_at	Reep5	**receptor accessory protein 5**	**-2,8**	0,000000
1418015_at	Pum2	**pumilio 2 (Drosophila)**	**-2,8**	0,000000
1452163_at	Ets1	**E26 avian leukemia oncogene 1, 5' domain**	**-2,8**	0,003713
1443341_at			**-2,8**	0,000000
1416568_a_at	Acin1	**apoptotic chromatin condensation inducer 1**	**-2,8**	0,000000
1427979_at	4732418C07Rik	**RIKEN cDNA 4732418C07 gene**	**-2,8**	0,000000
1436713_s_at	Meg3	**maternally expressed 3**	**-2,8**	0,000003
1418570_at	Ncstn	**nicastrin**	**-2,8**	0,000000
1441480_at	7120451J01Rik	**RIKEN cDNA 7120451J01 gene**	**-2,8**	0,000000

1458161_at	Kcnq1ot1	**KCNQ1 overlapping transcript 1**	**-2,8**	0,000088
1437405_a_at	Igfbp4	**insulin-like growth factor binding protein 4**	**-2,9**	0,008826
1450903_at	Rad23b	**RAD23b homolog (S. cerevisiae)**	**-2,9**	0,000000
1422853_at	Shc1	**src homology 2 domain-containing transforming protein C1**	**-2,9**	0,000000
1426208_x_at	Plagl1	**pleiomorphic adenoma gene-like 1**	**-2,9**	0,010471
1439068_at	Erap1	**endoplasmic reticulum aminopeptidase 1**	**-2,9**	0,000000
1439740_s_at	Uck2	**uridine-cytidine kinase 2**	**-2,9**	0,000006
1417440_at	Arid1a	**AT rich interactive domain 1A (SWI-like)**	**-3,0**	0,000000
1431214_at	LOC67527	**murine leukemia retrovirus**	**-3,1**	0,000000
1427385_s_at	Actn1	**actinin, alpha 1**	**-3,1**	0,000000
1445111_at			**-3,1**	0,000004
1423756_s_at	Igfbp4	**insulin-like growth factor binding protein 4**	**-3,1**	0,008142
1419313_at	Ccnt1	**cyclin T1**	**-3,2**	0,000000
1449370_at	Sox4	**SRY-box containing gene 4**	**-3,2**	0,003259
1417229_at	Capn1	**calpain 1**	**-3,2**	0,000000
1423060_at	Pa2g4	**proliferation-associated 2G4**	**-3,2**	0,000000
1450188_s_at	Lipg	**lipase, endothelial**	**-3,2**	0,000000
1437443_at	EG620382	**predicted gene, EG620382**	**-3,2**	0,000000
1460423_x_at	Igkv1-117	**immunoglobulin kappa chain variable 1-117**	**-3,2**	0,000431
1435664_at	Zfp397	**zinc finger protein 397**	**-3,3**	0,000011
1416239_at	Ass1	**argininosuccinate synthetase 1**	**-3,3**	0,000000
1415854_at	Kitl	**kit ligand**	**-3,3**	0,000000
1449184_at	Pglyrp1	**peptidoglycan recognition protein 1**	**-3,4**	0,000000
1446595_at	Itsn2	**intersectin 2**	**-3,5**	0,000000
1424254_at	Ifitm1	**interferon induced transmembrane protein 1**	**-3,5**	0,011034
1452415_at	Actn1	**actinin, alpha 1**	**-3,6**	0,000000
1416521_at	Sepw1	**selenoprotein W, muscle 1**	**-3,6**	0,000000
1431213_a_at	LOC67527	**murine leukemia retrovirus**	**-3,7**	0,000000
1459556_at			**-3,8**	0,000000
1419157_at	Sox4	**SRY-box containing gene 4**	**-3,8**	0,002579
1437479_x_at	Tbx3	**T-box 3**	**-3,9**	0,002979
1444258_at			**-3,9**	0,000000
1436780_at	Ogt	**O-linked N-acetylglucosamine (GlcNAc) transferase (UDP-N-acetylglucosamine:polypeptide-N-acetylgluco**	**-4,0**	0,000000
1449552_at	Zfr	**zinc finger RNA binding protein**	**-4,1**	0,000000
1458617_at	Prkcbp1	**protein kinase C binding protein 1**	**-4,5**	0,000003
1426936_at	LOC215866	**hypothetical protein LOC215866**	**-4,7**	0,000698
1442626_at			**-4,8**	0,000000
1418078_at	Psme3	**proteaseome (prosome, macropain) 28 subunit, 3**	**-5,3**	0,000000

List of differentially expressed genes assessed using the limma package from Bioconductor (Fold change>1.5 and adjusted p-value<0.05)
